# 2022 International Conference on Life, Health and Modern Medicine

**DOI:** 10.1097/MD.0000000000032897

**Published:** 2023-02-22

## LHMM001 AUTOMATED IDENTIFICATION AND QUANTIFICATION OF TRAUMATIC BRAIN INJURY FROM CT SCANS: ARE WE THERE YET?

Atsuhiro Hibi^a^, Majid Jaberipour^b^, Michael D. Cusimano^a,c^, Alexander Bilbily^b,d^, Rahul G. Krishnan^e,f^, Richard I. Aviv^g^, Pascal N. Tyrrell^a,b,h^

^*a*^ *Institute of Medical Science, University of Toronto, Toronto, Ontario, Canada,* ^*b*^ *Department of Medical Imaging, University of Toronto, Toronto, Ontario, Canada,* ^*c*^ *Division of Neurosurgery, St Michael’s Hospital, University of Toronto, Toronto, Canada,* ^*d*^ *Sunnybrook Health Sciences Centre, Toronto, Canada,* ^*e*^ *Department of Computer Science, University of Toronto, Toronto, Ontario, Canada,* ^*f*^ *Department of Laboratory Medicine & Pathobiology, University of Toronto, Toronto, Ontario, Canada,* ^*g*^ *Department of Radiology, Radiation Oncology and Medical Physics, University of Ottawa, Ottawa, Ontario, Canada,* ^*h*^ *Department of Statistical Sciences, University of Toronto, Toronto, Ontario, Canada*

**Objectives:** The purpose of this study was to conduct a systematic review for understanding the availability and limitations of artificial intelligence (AI) approaches that could automatically identify and quantify CT findings in traumatic brain injury (TBI).

**Methods:** Systematic review, in accordance with PRISMA 2020 and SPIRIT-AI extension guidelines, with a search of four databases (Medline, Embase, IEEE Xplore, and Web of Science) was performed to find AI studies that automated the clinical tasks for identifying and quantifying CT findings of TBI-related abnormalities.

**Results:** A total of 531 unique publications were reviewed, which resulted in 66 articles that met our inclusion criteria. The following components for identification and quantification regarding TBI were covered and automated by existing AI studies: identification of TBI-related abnormalities; classification of intracranial hemorrhage types; slice-, pixel-, and voxel-level localization of hemorrhage; measurement of midline shift; and measurement of hematoma volume. Automated identification of obliterated basal cisterns was not investigated in the existing AI studies. Most of the AI algorithms were based on deep neural networks that were trained on two- or three-dimensional CT imaging datasets.

**Conclusions:** We identified several TBI-related CT findings that can be automatically identified and quantified with AI. A combination of these techniques may provide useful tools to enhance reproducibility of TBI identification and quantification by supporting radiologists and clinicians in their TBI assessments and reducing subjective human factors.

**Acknowledgements**

This work was supported by a research grant from Nippon Steel Corporation (Fund Number 509533).

## LHMM002 ELECTRONIC PUBLIC HEALTH EMERGENCY MANAGEMENT (EPHEM): AN INNOVATIVE APPROACH FROM THE WHO EASTERN MEDITERRANEAN REGIONAL OFFICE

Ali Abdullah^a^, Osman El Mahal^a^, Huda Haidar Anan^a^, Samar Hammoud^a^, Dalia Samhouri^a^

^*a*^ *Country Health Emergency Preparedness and International Health Regulations (CPI), World Health Organization Health Emergency Programme (WHE), World Health Organisation Regional Office for the Eastern Mediterranean (WHO EMRO), Cairo, Egypt.*

**Background:** Countries continue to establish and rely on Public Health Emergency Operation Centres (PHEOCs) to facilitate a well-coordinated, multi sectorial, all hazard public health emergency management process. An information management system is a core component of a functioning PHEOC. To that end, World Health Organization Eastern Mediterranean Regional Office (WHO EMRO) developed the electronic Public Health Emergency Management (ePHEM). In Phase 1, the Online Signals Module, within ePHEM, was piloted in the WHO regional office and at country level and proved its practicality and functionality where it enhanced Watch and Alert Modes operations within the PHEOC.

**Methods:** A system analysis was conducted in order to identify the business requirements for the software through consultations with relevant technical experts in WHO and member states. A bi-regional meeting including both EMRO and African Regional Office (AFRO) with 34 countries participating resulted in building consensus and drafting the software’s business and functional requirements. The system prototype was developed (named ePHEM phase 1), modified and piloted in two settings, one at the regional office to support signal detection and management and one at PHEOC setting in Sudan to support the overall watch and alert functions of the PHEOC.

**Results:** Through piloting ePHEM Phase 1, the signal management has improved significantly. Staff for the first time were able to remotely manage signals while working from home. Scope of information, signal management, triage process, verification process, division of labor, documentation, accountability, validation, communication, access to information, user experience, monitoring and evaluation and data analysis has all seen improvements using ePHEM. ePHEM Phase 2 is ready for piloting in countries, awaiting conclusion of Phase 1 pilots.

**Conclusions:** Piloting ePHEM Phase 1 demonstrated the benefits of an online solution, enabling EMRO to remotely realize all Watch-Mode and Alert-Mode functions. Employing the software resulted in cleaner data, particularly when utilized by multiple users in addition to enforcing Standard Operational Procedures. Automated Dashboards assisted in decision making process. Available access to information improved overall awareness. A fitting tailored IT solution plays a key role in alertness, rapid decision making and response.

## LHMM003 COMMON TYPES OF UNREASONABLE MEDICAL ORDERS FOR INTRAVENOUS MEDICATION AND CONSTRUCTION OF INTERVENTION SYSTEM

Xuan Liang^a^

^*a*^ *The 940th Hospital of Joint Logistic Support Force of Chinese People’s Liberation Army, Lanzhou, Gansu, 730000, China*

**Background:** This study aims to enhance pharmacists’ ability to review intravenous drug prescriptions or medical orders, standardize clinical intravenous medication, maximally avoid the safety risks in unreasonable intravenous drug prescriptions or medical orders, standardize drug allocation and delivery process, and improve the rationality in intravenous medication.

**Subjects and Methods:** The unreasonable medical orders for intravenous medication in clinical practice were analyzed statistically, including drug concentration, dose, compatibility, inappropriate solvent, etc. Based on the workflow of “eight reviews and eight verifications,” the prescription was reviewed from 10 aspects of “drug concentration, dose, incompatibility, solvent, drug user, medication method, potential danger, whether skin test is needed, same drug with different drug name and drug name writing standard,” and verified from 10 aspects of “medical order, dispensing, drug division, warehouse entry, compatibility, warehouse exit, packaging, delivery, ward reception, and patient medication” to build the intervention system of “ten reviews and ten verifications.” Effort is needed to build information and feedback platform for intravenous clinical medication, strengthen the training of pharmacists’ professional ability to improve pharmacists’ intervention level and ability in clinical intravenous medication.

**Results:** The level and quality of rational intravenous drug use in hospitals are significantly improved, and unreasonable medical orders and irrational prescriptions before drug use are maintained a level of less than 0.015% in recent 5 years.

**Conclusions:** The “ten reviews and ten verifications” intervention system for intravenous medication maximally avoids irrational drug prescription or unreasonable medical orders, ensures the patient safety in intravenous medication, and provides referential experience for the safety of intravenous medication in hospitals.

## LHMM004 EFFECT OF PHYSICAL EXERCISE ON DEPRESSION OF COLLEGE STUDENTS

Dandan Chen^a^, Wen-Lung Chang^a^, Yang Wang^b^, Sha Pu Lu Bi^c^, Tzu-Yuan Chang^d^, Yue Wang^a^, Yao Liang^e^

^*a*^ *School of Education Science, Nanning Normal University, Nanning, China,* ^*b*^ *Wuzhou University, Wuzhou, China,* ^*c*^ *School of Physical & Health, Nanning Normal University, Nanning, China,* ^*d*^ *George Herbert Walker School of Business & Technology, Webster University, St. Louis, USA,* ^*e*^ *Guangxi University of Foreign Languages, Nanning, ChinaDandan Chen and Wen-Lung Chang contributed equally to this research.*

**Background:** This study through psychological tests on college students in Central China, studying and exploring the physiological and psychological mechanisms by which physical exercise activities interfere with depression, it is possible to effectively explore and reflect the fitness and heart health function of physical exercise activities, and provide a theoretical basis for effectively reducing negative emotions such as depression through physical exercise activities. From a practical point of view, persistent depression causes great harm to the individual’s physical and psychological aspects. If not effectively solved for a long time, it will incur a great impact on mental health. Therefore, it is quite necessary to actively explore ways to effectively reduce depression.

**Subjects and Methods:** 400 students from a university in central China and selected by random sampling to fill out the depression self-rating scale for psychological testing; and then 20 students showing significant depression were selected from the 400 students, who underwent experiment of interfering with depression and other negative emotions via continuous physical exercise activities. 20 college students were randomly divided into experimental group and control group while ensuring that gender ratio was roughly the same in the two groups. Researchers selected 20 college students with obvious depressive symptoms, divided them into two groups, the experimental group and the control group, and adopted running-based physical exercise for an 8-Researchersek test. Before and after the experiment, use “Depression Self-Rating Scale (SDS) to test the experimental group and the control group, and record the test data.

**Results:** First, college students have relatively high depression in overall. Second, there are gender differences in depression levels among college students, and female college students have slightly higher depression than male college students. Third, there are grade differences in the depression level of college students, and higher grade students have slightly higher depression than lower grade students. Fourth, continuous physical exercise activities can effectively reduce college students’ depression level, which play a good role in regulating the depression and other negative emotions of college students.

**Conclusions:** Researchers need strengthen the mental health education among junior and senior students. College students of different ages will develop different contradictions and pressures. Schools should conduct mental health education according to the contradiction and pressure characteristics of students at different stages, strengthen mental health education for juniors and seniors, give more lectures in this area, encourage them to face up to the development of bad emotions and reasonably vent such emotions. Finally, the test data shows that continuous physical activity can effectively alleviate college students’ depression tendency, create a positive effect in improving the body’s self-confidence and maintaining a good psychological state, which thus has good psychological benefits.

**Acknowledgments**

The authors acknowledge the 2020 Guangxi Graduate Education Innovation Plan Project “Research on the implementation status and Countermeasures of the Postgraduate Tutor System in Guangxi Universities in the new era” (JGY2020122).

## LHMM005 EFFECT OF ORAL PROSTHETIC NEGATIVE PRESSURE TECHNIQUE ON THE BONDING STRENGTH OF TRANSPARENT ZIRCONIA CERAMIC AND RESIN CEMENT

Zhao Wei^a^, Pei Wang^a^, Lin Ren^a^, Xue Yu^a^, Xuejuan Ma^a^, Yongping Ma^a^

^*a*^ *The NO.2 Hospital of Baoding, HeBei Baoding 071000, China*

**Objective:** To study the influence of negative pressure on the bonding strength of transparent zirconia ceramics and resin cement.

**Methods:** 36 polished transparent zirconia specimens were randomly divided into 4 groups (n=9): control group (C) without surface treatment; sandblasting group (SB), 50 μ mAl2O3Unisand blasting for 20s; negative pressure group (SU), the specimen is treated with 0.04MPa negative pressure; sand blasting + negative pressure group (SSU), the specimen is treated with negative pressure treatment after coating treatment agent. One randomly selected specimen was used for scanning electron microscope (SEM) for surface observation, and the other specimens were used for shear strength test by universal mechanical experiment machine. The fracture mode was observed under a stereopscope microscope, After the shear test, the fracture modes were observed under the body-type microscope (20). The specimen fracture modes were divided into three types. I Cohesin ring: fracture interface occurs in resin layer or porcelain layer; bonding failure (interface failure): fracture interface occurs in the bonding interface of transparent zirconia and valve; mixing failure: both bonding and mixed damage. And the results were statistically analyzed, data were analyzed using SPSS 26.0 statistical software, SBS data analysis of variance with normality test using K-S test and one-way ANOVA test and group comparisons were performed using Dunnett’S T3. Break patterns data using Fisher’s exact probability test. All the test levels were α=0.05.

**Results:** SEM results showed that the adhesive surface of transparent zirconia changed significantly in the sandblasting group, the negative pressure group was not different from the control group, and the distribution of the specimen surface adhesive after negative pressure treatment was more uniform. The SB (18.54 ± 0.88) and SSU group (20.74 ± 2.00) were relative to the SU group (p <0.05), and the SU group of SSU group was significantly stronger than the SB group, which was statistically significant (p <0.05), except that the SSU group had the most mixed damage.

**Conclusion:** Sandblasting treatment can effectively improve the bonding strength between transparent zirconia and resin adhesive. Although the negative pressure technology alone cannot improve the bonding effect, the bonding strength can be significantly improved when combined with sandblasting.

## LHMM006 EFFECTS OF TREADMILL EXERCISE ON EXPRESSION OF ERK1/2, CREB AND BDNF IN HIPPOCAMPUS AND MEMORY ABILITY OF PARKINSON’S DISEASE MODEL MICE

Zhijie Wang^a^, Yan Fu^a^, Kang Zhang^b^

^*a*^ *Institute of Physical Education, Southwest Minzu University, Chengdu, Sichuan 610225, China,* ^*b*^ *Institute of Physical Education, Sichuan Normal University, Chengdu, Sichuan 610066, China.*

**Background:** Parkinson’s disease (PD) is a neurodegenerative disease. We explored the effects of aerobic exercise intervention on learning and memory ability of PD model mice and revealed the significance of ERK1/2, CREB and BDNF in the prevention and improvement of learning and memory ability of PD mice by aerobic exercise.

**Subjects and Methods:** In the present study, female C57BL/ 6 mice aged 8 weeks were randomly assigned to 6 groups, each with 12 mice. After 7 weeks of treadmill exercise, mice’s learning and memory abilities were assessed using the Morris water maze test, and the expression of ERK1/2, CREB and BDNF in the hippocampus was detected using Western and RT-PCR.

**Results:** The number of mice crossing the original platform was lowest in the PD group, which in PDE group was s gnificantly higher than that in PD group (P < 0.05). At the same time, the swimming distance of original platform quadrant of mice in PD group was significantly shorter than that in SC group, and the swimming distance of original platform quadrant of mice in PDE group was significantly higher than that in PD group (P<0.05). The expression of ERK1/2 protein in PD group was significantly lower than that in SC group (P<0.05); Compared with PD group, the expression levels of ERK1/2 and CREB in PDE group were significantly increased (P<0.01). The expression of BDNF protein in hippocampal neurons of PD group was lower than that of SC group, but the difference was not significant, and the decrease degree was about 22.73%. The expression of total BDNF protein in hippocampus of PDE group was significantly increased (P<0.01).The mRNA expressions of ERK1/2 and CREB in PDE group were significantly higher than those in PD group (P<0.05). The mRNA expression of BDNF in PD group was significantly lower than that in SC group (P<0.01), and the relative expression was 0.72 ± 0.03 times that in SC group. Compared with PD group, the expression of BDNF mRNA in PDE group was significantly up-regulated (P<0.01).

**Conclusions:** The PD model mice have learning and memory impairment, as well as reduced expression of ERK1/2, CREB and BDNF protein and genes. Aerobic exercise intervention for 7 weeks can prevent and slow down the impairment of learning and memory ability in PD mice by improving ERK1/2, CREB and BDNF protein and genes expression.

**Acknowledgments**

This work was supported by a project grant from a Basic Scientific Research Project of Central Universities, Southwest Minzu Universities (Grant NO.2020NYB31).

## LHMM007 ANALYSIS OF THE MEDICATION RULES OF TRADITIONAL CHINESE MEDICINE IN THE TREATMENT OF CHILDREN’S ABNORMAL BLIMK BASED ON DATA MINING

Yajuan Li^a^, Huijuan Gao^a^, Miaomiao Wang^a^, Bingfan Miao^a^, Lijuan Sun^b^

^*a*^ *HanDan AiYan Ophthalmology Hospital, Hebei 056008, China,* ^*b*^ *AiYan Ophthalmology Hospital Management(Beijing)Co., Ltd. Beijing 100010, China.*

**Objective:** To explore the rule of traditional Chinese medicine (TCM) in the treatment of children’s abnormal blink based on Data Mining.

**Methods:** Relevant papers published from the establishment of the database of CNKI, Wanfang, VIP, and Chinese Biomedical Literature Database to December 1, 2022, were retrieved. The title, author, sample size, drugs contained in the prescription, and efficacy information of the paper are entered into WPS Excel to establish a database.WPS Excel carried out descriptive statistics on the frequency of prescription drugs and the Nature and Flavor of the medicine. SPSS Statistics was used for systematic cluster analysis; The Apriori algorithm in IBM SPSS Modeler 18.0 performed association analysis and made a network diagram.

**Results:** (1) General situation: 48 articles were finally included, including 66 Chinese medicine prescriptions, involving 111 kinds of TCM, with a cumulative frequency of 697. Among them, 22 kinds of TCM are used more than 10 times, 432 times in total, which was defined as high-frequency TCM. The top three with the highest frequency were Baked Licorice (46 times, 69.70%), Poria Cocos(40 times, 60.61%), and White Peony(37 times, 56.06%). (2) Classification of the efficacy of high-frequency TCM: There are 8 types of efficacy involved, with the most tonic drugs (150 times, accounting for 34.72%), followed by calming liver wind drugs (71 times, accounting for 16.44%), relieving exterior syndrome drugs and digestive drugs (67 times, accounting for 15.51%). (3)The drug properties were mainly bland(8 times, 36.36%), the flavors were mainly sweet (16 times, 72.73%), pertained meridians were mostly liver and spleen (13 times each, accounting for 23.64%).(4) Cluster analysis of high-frequency Chinese medicines: Six types of combinations were obtained, Poria Cocos-Rhizoma Atractylodis Macrocephala-Radix Pseudostellariae; Malt-Hawthorn- Medicated Leaven- Picrorhiza- Rhizoma Atractylodis- Endothelium Corneum Gigeriae Galli; Saposhnikovia Divaricata- Cicada Slough; White Peony- Angelica Sinensis; Gastrodia Elata-Bombyx Batryticatus- Scorpio- Uncaria Hook- Baked Licorice; Ophiopogon Japonicus- Mulberry Leaves-Chrysanthemum. (5) Correlation analysis of high-frequency Chinese medicines: five types of drug pairs were obtained, including invigorating the spleen to eliminate dampness, softening the liver and nourishing the spleen, calming liver wind, invigorating stomach and digestion, and dredging wind and relieving spasm.

**Conclusion:** TCM treatment of children’s abnormal blink focuses on the liver and spleen, focusing on strengthening the spleen and eliminating accumulation, calming liver wind, dispelling wind and stopping convulsions.

## LHMM008 STUDY ON SPORTS ABILITY AND PHYSICAL HEALTH DEVELOPMENT OF RURAL CHILDREN IN DIANJIANG COUNTY

Qixia Jia^a^, Pengxin Gu^a^, Yongyong Wang^a^

^*a*^ *Department of Sports, Chongqing Jiaotong University, Chongqing 400074, Chongqing, China*

**Background:** At present, with the continuous development of society, more and more attention is paid to public health. Healthy lifestyles include good hygiene habits, regular sleep habits, balanced nutritional intake, scientific and reasonable physical exercise, etc. Similarly, many scholars attach great importance to children’s physical and mental health, and the development of children’s physical health and athletic ability has received more and more attention. To understand the physical exercise ability and physical health status of rural children in different source areas, we selected rural children in the Southwest Mountain Area (Dianjiang County) for this study.

**Subjects and Methods:** In this study, we selected 300 boys and 300 girls, with an average age of 10 years. The Test index include height and weight, vital capacity, 50-meter run, Sit-and-reach, 1 min rope skipping, 50m×8 round trip. The test results were compared with the average of the same age group in the “2014 National Physical constitution Fitness Supervision Bulletin.”

**Results:** The results showed that the average height and weight of the subjects were significantly lower than the national average. The average height of boys was 5.3cm shorter and their weight was 2kg lighter, while the average height of girls was 3.5cm shorter and their weight was 0.7kg lighter; The respiratory function and exercise ability were significantly better than the national average. The average vital capacity of boys was 556.9ml higher and that of girls was 721.9ml higher; Boys’ 50-meter run is 0.3s faster than the national average, and girls’ 50-meter run is 0.7s faster than the national average; The boy’s Sit-and-reach is 4.8 cm better than the national average level; The endurance project 50m×8 round trip running of boys was 7.6 seconds faster than the national average, and girls was 10.8 seconds faster than the national average.

**Conclusions:** Influenced by regional economic development, diet and nutrition, the development of height and weight of the subjects was significantly lower than the national average. Children in mountain areas will do a lot of sports in their daily life, which is very helpful for the development of respiratory function and sport ability.

**Acknowledgments**

This work was supported by Rural Sports Development Research Center of Fujian College Humanity and Social Sciences Research Base (Minnan normal University) (20NTKT010).

## LHMM009 IDENTIFICATION MODEL AND VALIDATION EVALUATION OF DIABETIC RETINOPATHY BASED ON DEEP RESIDUAL NEURAL NETWORK

Enguang Ding^a^, Enliang Ding^a^, Lijuan Sun^b^, Shuoyu Li^b^, Qilong Zhao^a^

^*a*^ *HanDan AiYan Ophthalmology Hospital, Handan, China,* ^*b*^ *AiYan Ophthalmology Hospital Management (Beijing) Co., Ltd. Beijing, China*

**Objective:** To construct the identification model of diabetic retinopathy (DR) through the deep residual neural network (ResNet-34) to diagnose and grade DR.

**Methods:** The 3662 DR pictures published by the fourth Asia Pacific Remote Ophthalmology Society (APOTS) were taken as data samples. The samples were divided into normal, mild, moderate, severe, and proliferation according to DR classification. 60% of the data samples were selected as the training set, 20% as the test set, and the remaining 20% as the validation set. First, conduct data balance, then build ResNet34, input the data into them to train the classification identification model; then the data samples are divided into two categories, namely, DR, to obtain the disease identification model; randomly select 205 fundus images in the validation set and compare the two models with the ophthalmologist to calculate the identification time and accuracy.

**Result:** The identification sensitivity (recall rate) of the result classification and disease identification model was 96.94% and 99.80%, the accuracy rate was 98.29% and 99.79%, the accuracy rate was 96.35 and 99.78%, and the f1 value was 96.63% and 99.79%. The hierarchical identification model was 0.07s, and the total service time used was 15.35s; The single reading time of the binary identification model was 0.06s, Total use time of 12.29s; The diagnostic accuracy of primary, middle and senior titles in ophthalmology was 92.70%, 94.40%, and 95.60%, respectively, The average single film reading time is 3.96s,2.68s,3.45s, The total time used is 13.53m,9.14m,11.77m; In the second taxonomy, The accuracy rate of primary, middle and senior professional titles in ophthalmology was 97.00%,100%,100%, The average single time is 1.6s,1.3s,1.2s, Total service time of 5.5mim, 4.4min, 4.1min.

**Conclusion:** The DR recognition model constructed by ResNet34 has obvious advantages in recognition performance and time, which can be used for screening, diagnosis, and treatment of DR.

**Acknowledgements**

“Clinical Study on the Correlation between Serum and Intraocular VEGF and Inflammatory Factors in Patients with diabetes Retinopathy” Health Commission of Hebei Province (20200416)

## LHMM010 DIETARY SUPPLEMENTATION WITH PRODUCTS OF CITRUS RETICULATA “CHACHI” FOR IMPROVING FECAL MICROBIOME OF WEANED PIGLETS

Huafeng Lin^a,b^, Gang Li^c^, Haizhen Wang^d^, Lili Li^a^, Xin Yuan^e^, Lei Shi^a^, Xiangwen Peng^b^, Yanlei Chang^a^, Jie Yang^f^, Yuqi Liu^a^, Hanyue Gong^a^

^*a*^ *Institute of Food Safety and Nutrition, Jinan University, Guangzhou 510632, China,* ^*b*^ *Changsha Hospital for Maternal and Child Health Care of Hunan Normal University, Changsha 410007, China,* ^*c*^ *Institute of Biomedicine and Department of Cell Biology, Jinan University, Guangzhou 510632, China,* ^*d*^ *Department of Life Science, Lv Liang University, Lvliang 033000, China,* ^*e*^ *The First Affiliated Hospital of Hunan University of Traditional Chinese Medicine, Changsha 410021, China,* ^*f*^ *Key Laboratory of Functional Protein Research of Guangdong Higher Education Institutes, Institute of Life and Health Engineering, College of Life Science and Technology, Jinan University, Guangzhou 510632, China.HL, GL, HW contributed equally to this work.*

**Background:** Nutritional interventions play a critical role in modifying the intestinal microbiome of the host animals. This study aimed at elucidating the physiological effects of the dietary supplemented with two types of products of Citrus reticulata “Chachi” on the fecal microflora of weaned piglets.

**Subjects and Methods:** A total of 72 piglets with uniform sizes were randomly assigned to four dietary treatment groups, viz., negative control group (NCG), fermented Citrus reticulata “Chachi” pulp group (FCRPG), Citri Reticulatae Pericarpium group (CRPG), and positive control group (PCG) in a 21-day feeding trial. After the raising experiment, fresh feces of the piglets were systematically analyzed using the multi-omics technologies.

**Results:** Metagenomics method equipped with high-throughput compositional characterization indicated that these two additives and compound antibiotics influenced both the architecture and diversity of the fecal microbiome. The metabolite analysis demonstrated that FCRPG exhibits a significant effect on the fecal short-chain fatty acids (SCFAs) among the four treatment groups. Results of the functional proteomics approaches revealed that FCRPG exhibited the highest butyrate metabolic level, while CRPG exhibited the highest flavone and flavonol biosynthesis level in the feces. In addition, the combined antibiotics were effective in adjusting the fecal microbiota profile.

**Conclusions:** Our findings demonstrated that dietary supplementation with FCRP or CRP could modulate the microbial taxa, metabolic, and proteomic alterations in the fecal microbiota of weaned piglets.

**Acknowledgments**

This research was supported in part by grants from the National Key Research and Development Plan (2016YFD0500600), Guangdong Provincial Science and Technology Plan Project (2017B020207004), Youth fund project of Hunan natural science foundation (2019JJ50681) and Changsha biological resources sample bank establishment project (20200365).

## LHMM011 EXPLORATION OF THE VIRTUAL SIMULATION EXPERIMENT OF NUTRITIONAL EVALUATION AND DIETARY GUIDANCE OF HYPERLIPIDEMIA PATIENTS UNDER THE NEW MEDICAL BACKGRONUD

Shan Cao^a^, Manjie Qiao^b^, Lanying Pei^b^

^*a*^ *School of Nursing, Henan University of Chinese Medicine, Zhengzhou 450046, China,* ^*b*^ *School of Medicine, Henan University of Chinese Medicine, Zhengzhou 450046, China.*

**Background:** To use virtual simulation technology to construct “ virtual simulation experiment project of nutritional status evaluation and dietary guidance of integrated traditional Chinese and Western medicine of hyperlipidemia patients “ under the background of new medicine science, with the purpose of creating high-quality virtual simulation experiment teaching resources and cultivating innovative and compound medical talents.

**Subjects and Methods:** This paper expounds how to use virtual simulation software to evaluate the nutritional status of patients with hyperlipidemia and how to use traditional Chinese and Western medicine to provide dietary guidance from the aspects of project construction background, design ideas of experimental scheme and construction of platform framework of core elements, and summarizes the specific operation process of the project experiment, the teaching mode and the evaluation method, the project characteristics and the application feedback. In the aspect of project application evaluation, questionnaire survey was conducted among students who had used the software, and subjective and objective evaluation was conducted on the improvement of nutrition assessment skills, the mastery of integrated Chinese and western medicine knowledge and the effect of project application.

**Results:** A total of 296 people were surveyed, 261 questionnaires were collected, and the response rate was 88.12%. The objective evaluation was carried out in seven aspects, the results showed that the program could effectively make up for the shortcomings of traditional experimental teaching, and the students had a high degree of satisfaction in stimulating learning interest (more than 90%), deepening understanding of nutrition assessment knowledge, improving traditional Chinese and Western medicine diagnosis and dietary guidance skills, cultivating systematic thinking ability of medical profession and dialectical thinking of TCM. In terms of team cooperation and communication skills, the degree of satisfaction was slightly lower (82.76% and 86.21%). The subjective evaluation investigated two parts of the virtual simulation experiment that students thought were the most advanced advantages and the aspects to be improved, and finally concluded that the top three advantages of the project were immersion, convenience, understanding of theoretical knowledge and expansion advantages. The top three disadvantages are the fluency of operation, the realism of picture and the flexibility of module exercise.

**Conclusion:** The development and application of this project can effectively make up for the shortcomings of traditional education mode, give full play to the advantages of high-quality online teaching resources in the period of COVID-19, and provide a strong guarantee for the construction of a lifelong learning system of new medicine, and contribute to the training of high-quality, innovative and compound medical talents in the New Era.

**Acknowledgments**

This work was supported by The first batch of production school cooperation and collaborative education project of the Ministry of Education in 2021 (Grant No. 202101237022); Key Project of Research and Practice of Higher Education Teaching Reform in Henan Province (Grant No. 2021SJGLX179); Henan Higher Education Teaching Reform Research and Practice Project (Grant No. 2021SJGLX175Y); Education Research Project of the Teaching Steering Committee of Integrated Traditional and Western Medicine in Colleges and Universities of the Ministry of Education(Grant No. 2022YB-02).

## LHMM012 THE RECOVERY TIME AFTER SARS-COV-2 VARIANTS INFECTION: A RETROSPECTIVE STUDY

Ping Lu^a^, Xuchang Zhang^b^, Xiangyan Zhang^a^, Yiling Zhang^a^, Hongmei Yao^a^, Cheng Zhang^a^, Bangyan Zhang^a^, Xianwei Ye^a^

^*a*^ *Guizhou Provincial People’s Hospital, Guiyang 550000, China,* ^*b*^ *Second Affiliated Hospital of the Chinese University of Hong Kong, Shenzhen 518071, ChinaPing Lu and Xuchang Zhang contributed equally to this study.*

**Background:** Defining the duration of infectivity of Severe Acute Respiratory Syndrome Coronavirus 2 (SARS-CoV-2) has major implications for public health and infection control practice in healthcare facilities. SARS-CoV-2 appears to be most contagious around the time of symptom onset, and infectivity rapidly decreases thereafter to near-zero after about 10 days in mild-moderately ill patients and 15 days in severely-critically ill and immunocompromised patients. The longest interval associated with replication-competent virus thus far is 20 days from symptom onset. Based on current evidence, recent guidelines indicated the use of Paxlovid and Azvudine for the treatment of COVID-19 patients with mild-moderate disease, not requiring high-flow oxygen.

**Subjects and Methods:** The purpose of this study was to assess symptomatic treatment and the clinical outcomes for COVID-19 patients mainly regarding to the recovery time and rate, thus, helping to identify the optimal management of the disease.

**Methods:** 225 hospitalized patients in Guizhou Provincial People’s Hospital (11 of them were non-vaccinated) during 17th August to 20th September 2022 were diagnosed for COVID-19 using the total RNA extracted from NPSs through the amplification of SARS-CoV-2 ORF1ab and N gene by RT-qPCR. All the patients were confirmed with mild and moderate cases, except 31 among them administrated with Paxlovid and Azvudine tablets, others treated symptomatically.

**Results:** Among the 225 patients in the study, 9.3% (21 cases) were in intensive care units, 25.3% (57 cases) were mild/common cases, 65.4% (147 cases) were asymptomatic patients. There were 9, 11 and 12 patients have the complications such as hypertension, diabetes, coronary heart disease and renal failure in asymptomatic, mild and severe groups, separately. 3, 11 and 12 cases have not vaccinated in asymptomatic, mild and severe groups, separately. Average recovery time (presenting negative RNA test outcome) was 12.36 days (ranges: 3-30 days), there was no significant difference among these three groups regarding the recovering time, whether they were administrated with Paxlovid and Azvudine tablets or not.

**Conclusions:** We considered that the average recovery time of virus is a independent factor, which seemly has not associated with many indicators, such as age, gender, severity of illness and the drug administration.

**Acknowledgments**

This work was supported by the Special Fund for Basic Scientific Research Fee of Central Public Welfare Research Institute of Chinese Academy of Medical Sciences (2019PT320003) and Medical and Health Science and Technology Innovation Project of Chinese Academy of Medical Sciences, Guizhou Group Medical Research Site Construction and Medical and Health Integration Construction Model Research (2020-I2M-2-003).

## LHMM013 RESEARCH ON THE TRAINING MODE OF “REHABILITATION + EDUCATION” COMPOSITE REHABILITATION TRAINERS FOR SPECIAL CHILDREN

Yang Lu^a^, Zhonghui Luo^a^

^*a*^ *Department of special education, Zhejiang Vocational College of special education, Hangzhou, Zhejiang, 310023, China.*

**Background:** Caring for children with disabilities, focusing on the cultivation of education and rehabilitation talents for children with autism spectrum disorders. Aiming at the improvement of the education and rehabilitation level of children with disabilities, through the exploration and research of the training path of special education teachers, Provide high-quality teacher training mode for the education and rehabilitation of special children.

**Subjects and Methods:** Relying on the opportunity of the construction of double high professional groups in special education, A series of in-depth reforms and innovations have been carried out in the fields of talent training programs, teaching material construction, curriculum construction, teaching resource construction, school enterprise cooperation, etc., to improve the quality of professional talent training, from rehabilitation, education and other dimensions, create a new path for the cultivation of special education teachers with the combination of health and education.

**Results:** The training quality of normal school students has been improved year by year. It has formed a talent training mode of “health, education and service” for professional groups, built a curriculum system for professional groups, completed the construction of professional groups of teachers, formed a school enterprise cooperation form of “government, school, enterprise and practice,” and made breakthroughs in curriculum construction, practical teaching base construction, etc, and the cultivation of health education talents for special children aged 0-6 has begun to take shape.

**Conclusions:** The demand for education and rehabilitation teachers for special children is imminent, and the training of compound special education teachers is also the direction that needs attention in the future. Special education teachers with the abilities of rehabilitation, education and comprehensive auxiliary services are the main direction of talent training in the future. In the exploration of professional group construction, based on serving children with special needs, people with disabilities and their families, the connotation of special education is extended and expanded, and the provision of education + rehabilitation + other special services for children with special needs, people with disabilities and their families is taken as the starting point of professional talent training, sorting out special education. The group logic between rehabilitation therapy technology and sign language translation (barrier free service) and the design of professional groups are helpful to explore the new path of talent training mode and implementation of “special education +.”

**Acknowledgments**

This research was supported by a project grant from: The ideological and political teaching team of special education courses organized by the grass-roots teaching organization of Zhejiang Province in 2022, Project initiation time: November 2022.

## LHMM014 PROFESSIONAL IDENTITY OF FRONT-LINE NURSES IN PUBLIC HEALTH EMERGENCIES: BASED ON MENTAL HEALTH EVALUATION

Yu Wang^a^, Jingwen Zhang^a^, Xingmiao Feng^a^, Zhongjun Guan^a,b^, Kai Meng^a^

^*a*^ *School of Public Health, Capital Medical University, BeiJing, China,* ^*b*^ *Beijing Tiantan Hospital, Capital Medical University, BeiJing, China*

**Background:** The coronavirus pneumonia outbreak is highly contagious and classified as a Public Health Emergency of International Concern (PHEIC). According to a self-designed questionnaire to illustrate the professional identity of nurses on mental health evaluation and to research whether the professional identity of nurses who are on the front-line is higher than that of those who are not on the front-line. During the coronavirus pneumonia epidemic, nurses are the indispensable backbone of the front-line of the fight against the epidemic and are recognized by the whole society. Despite the hard work of nursing staff, the nursing profession is not respected by everyone, resulting in a low sense of professional identity among nursing staff. Society should be concentrated on their mental health evaluation. Then, does participation in front-line treatment of coronavirus pneumonia affect the professional identity of nurses?

**Methods:** In this study, the differences in professional identity among nurses in designated and non-designated hospitals were investigated through a survey in two public hospitals in Beijing. Hospital Y had a total of 1,529 employees, including 600 nurses, and 750 beds. And Hospital S had a total of 1,141 employees, including 402 nurses, and 450 beds. A self-designed questionnaire and the Nurses’ Professional Identity Evaluation Scale were used to measure professional identity of 474 nurses. And univariate analysis and multiple linear regression were used to research whether participation in front-line care improved nurses’ professional identity. Also, the researchers analyzed occupational value and occupational respect in occupational identity, and again whether the nursing staff was on the front line or not had no effect on their occupational value and occupational respect.

**Results:** There was no significant effect of participation in the front-line treatment of patients with coronavirus pneumonia on the professional identity of nurses (p>0.05). Of 474 nurses in this study, age and employment types influence the professional identity in univariate analysis. In multiple linear regression, employment types (p<0.05) influence professional identity in all dimensions, and different departments (p<0.01) also influence professional identity.

**Conclusion:** Whether or not to participate in the front-line treatment of the coronavirus pneumonia epidemic did not affect on the nurses’ professional identity. There was an increase in the identity of the nursing profession during the epidemic, with nursing staff feeling proud to be in nursing. Society also needs to focus on the mental health of caregivers, especially during special times.

**Acknowledgements**

Funding Information: Study on the ideas and measures to promote the construction of “Health Shunyi” during the 14th Five-Year Plan, and China Scholarship Council.

## LHMM015 INTRODUCTION OF DOMAIN KNOWLEDGE IN THE PROCESS OF ARTIFICIAL SIMULATION FOR AN ENRICHED RARE DISEASE DATASET

Mauro Mendez^a^, Felipe Castillo^b^, Linda Probyn^c^, Michael Brudno^d^, Alan R. Moody^c^, Pascal N. Tyrrell^a,c,e^

^*a*^ *Institute of Medical Science, University of Toronto, Toronto M5S1A8, Canada,* ^*b*^ *Department of Radiology, Hospital Clínico Pontificia Universidad Católica de Chile, Santiago 340, Chile,* ^*c*^ *Department of Medical Imaging, University of Toronto, Toronto M5T1W7, Canada,* ^*d*^ *Department of Computer Science, University of Toronto, Toronto M5S2E4, Canada,* ^*e*^ *Department of Statistical Sciences, University of Toronto, Toronto M5S3G3, Canada.*

**Background:** Research on rare diseases is often disregarded by the artificial intelligence (AI) field due to the availability of data for training. Hemophilia is a rare inherited bleeding disorder that can be life threatening and affects quality of life. Frequent bleeding in the joints (hemarthrosis) leads to joint pain and ultimately damage. Automated early detection of hemarthrosis using ultrasound can accelerate time to treatment, limit damage to the joints and improve quality of life. Synthetic data has been proposed as a solution for the limited availability of images required for AI training; however, due to the small sample of images, missing domain knowledge in the original dataset is often a serious limitation.

**Methods:** This study proposes a novel artificial generation framework capable of introducing domain knowledge to the synthesis of a rare disease dataset. A two-step generative pipeline was trained using state-of-the-art generative adversarial networks (i.e., StyleGAN2-ADA, FUNIT).

**Results:** By separating the problem in content and context generation, our framework showed satisfactory results in the simulation of recess distension and hemarthrosis ultrasound under the limitations of a rare disease. Synthetic images were evaluated using a blinded human trial, Fréchet Inception Distance (64.79 ± 0.03), and training a convolutional classification model (accuracy 85.47%, sensitivity 74.36%, specificity 96.58%, and an overall improvement of 31% compared to using only real data). Results showed no significant difference between the real and synthetic datasets based on two expert assessments. **Conclusions:** The use of our enriched synthetic images showed an improvement in AI training compared to regular data augmentation. Our framework advances AI research in the study of rare diseases and the prototyping of AI solutions in healthcare.

**Acknowledgement:** This work was supported by Novo Nordisk.

## LHMM016 EFFECT OF ETHANOL EXTRACT OF HIPPOPHAE RHAMNOIDES LEAF ON STREPTOZOTOCIN-INDUCED PLASMA LIPID METABOLISM IN TYPE II DIABETIC MICE

Yang Yang^a^, Haoni Li^a^, Hui Li^a^

^*a*^ *School of Agronomy and Life Sciences, Shanxi Datong University, Datong, 037009, China*

**Background:** As a metabolic disease, diabetes is classified into two types, type I diabetes and type II diabetes, whose main feature is high blood glucose levels. Hippophae rhamnoides Linn., a deciduous shrub with no part wasted, mainly features drought tolerance, salt-alkali tolerance and sand resistance. As a drug, Hippophae rhamnoides Linn. plays an important role in the treatment of various diseases. To study the effect of ethanol extract from Hippophae rhamnoides leaves on blood lipid metabolism in streptozotocin (STZ)-induced type II diabetic mice.

**Subjects and Methods:** The mice were fed with high-sugar and high-lipid feed for one month, then STZ (20 mg/kg) causes diabetes in mice, the mice with higher blood glucose were selected (the mice models with blood glucose ≥9 mmol/L were screened), the mice were grouped, and the ethanol extract from Hippophae rhamnoides leaves (4 mg/kg, 20 mg/kg and 100 mg/kg) was grouped according to the blood glucose interval. **Results:** The ethanol extract of the Hippophae rhamnoides leaves was gavage once a day into the mice, the blood glucose of the mice was recorded, Hippophae rhamnoides leaves had lower biochemical parameter levels, the contents of triacylglycerol, total cholesterol and arteriosclerosis index than diseased mice. However, the ethanol extract from Hippophae rhamnoides leaves had a positive effect on the content of high-density lipoprotein in diabetes mice.

**Conclusions:** The experimentally measured data indicated that the ethanol extract of Hippophae rhamnoides leaves had a blood glucose lowering effect on type II diabetic rats. It is related to the lipid metabolism mechanism in mice, also. Our data provide basic data for future research, and also provide certain theoretical research for the development of health food based on Hippophae rhamnoides leaves.

**Acknowledgments**

This research project supported by Shanxi Scholarship Council of China (HGKY2019090).

## LHMM017 A STUDY ON AT-HOME PHYSICAL EXERCISE FOR HEALTH AMONG PRIMARY ON MAJOR PUBLIC HEALTH EVENT DURING

Wei Yue^a^

^*a*^ *Physical Education School of Jilin Normal University, Jilin Siping 136000,China*

**Background:** A major public health event, which has emerged after severe acute respiratory syndrome-coronavirus (SARS-CoV) and middle east respiratory syndrome-coronavirus (MERS-CoV), causes severe respiratory illness in humans. It poses a serious threat to human life and safety and economic loss

**Subjects and Methods:** By using the methods of literature and interview, this paper makes an investigation and research on the occurrence of large public health events, the emergency measures adopted by primary and secondary schools, the use of offline teaching, students’ sports and healthy life style, put forward to adopt the idea of health, active participation in sports to promote health and other measures.

**Results:** The novel coronavirus is a major issue facing school sports, and schools can therefore only take classes online. In the face of the current pneumonia pandemic control of the novel coronavirus infection, in addition to effective prevention, primary and secondary school students have to keep in a good mood. For students, it is very important to strengthen their physique through physical exercise. Strengthening physical fitness can improve the immune system of the human body. Physical exercise promotes health to resist the invasion of viruses.

**Conclusions:** Primary and secondary school students are at a critical stage of growth and development. It is necessary to strengthen the health promotion in primary and secondary schools and to strengthen the physique of young people in order to promote the healthy growth and all-round development of primary and secondary school students. Ensure adequate physical activity, reduce sedentary and video (watching TV, using computers, cell phones, etc.) time. During the break, leave your seat and do some activities. Every day, accumulate at least 1 hour of moderate intensity and above exercise, cultivate the habit of lifelong exercise.

## LHMM018 HEALTHY DIGITAL TWIN SPACE: THE PATH OF ENVIRONMENTAL DESIGN TO DEAL WITH POPULATION AGING

Zheng Wang^a^, Guobin Wei^b^

^*a*^ *Zhejiang Guangsha Vocational and Technical University of Construction, Jinhua 322100, China,* ^*b*^ *Anhui University of Finance and Economics, Bengbu 233030, China*

**Background:** At present, population aging has become a hot social problem all over the world. From the perspective of the development process of developed countries, this is an inevitable result of the progress of social science and technology and the improvement of living standards. In the future, the aging of the global population will continue to intensify, and will have a compound impact on social development, including: increased social burden, the development of social and cultural welfare undertakings does not adapt to the aging of the population, the weakening of the family pension function, and the outstanding demand of the elderly for medical care and life services. In essence, design is a method to deal with practical needs and solve social problems. It is necessary to pay attention to the application of design in the field of aging problems.

**Subjects and Methods:** The living space of the elderly directly affects the physical and mental health of the elderly. On the one hand, the design of healthy digital twin space for the elderly is to improve the quality of the survival and development of the elderly. On the other hand, it can deal with emergencies, predict and deal with the elderly in time before they need medical treatment, and ensure their health. By analyzing the connection between pension environment design and pension groups, this project tries to deduce the application of digital twin technology in pension environment, explores the construction of healthy digital twin space, provides security for the physiological needs, psychological needs and emotional needs of the elderly, and provides environmental design schemes.

**Results:** Three types of elderly care environment were analyzed in the research process, and it was found that environmental design played a certain role in coping with social problems caused by population aging. How to cope with the material needs of the elderly through the application of new technologies to upgrade the elderly care environment.

**Conclusions:** The design concept of the healthy digital twin space is more perfect than the traditional elderly care environment in terms of elderly care services, and the expected effect is more prominent, which deserves the attention of the academic community.

## LHMM019 REVIEW OF INNOVATIVE RESEARCH ON MENTAL HEALTH EDUCATION FOR UNIVERSITY STUDENTS UNDER THE NORMALIZATION OF EPIDEMICS

Yi Wang^a^

^*a*^ *College of Science & Technology Ningbo University, Ningbo, Zhejiang 315300,China*

**Background:** How to effectively promote college students’ mental health education under the background of the normalization of epidemic is a distinct trend of China’s college students’ psychological education work, and is also a historical necessity related to the realization of the goal of establishing moral education.

**Subjects and Methods:** In order to meet the further growth needs of university students, we need to conduct an innovative analysis of mental health education for university students, which is of great value and significance. Based on the existing theoretical results and practical experiences, this study carefully reviews the literature on mental health education, ideological and political education, working methods of universities and specific contents of university students under the normalization of the epidemic.

**Results:** In recent years, students’ mental health education and curriculum thinking politics have become a hot spot of social concern, and scholars in China have conducted multi-dimensional and multi-angle discussions and studies on the direction of mental health education and ideological and political education of college students, among which the scope of research on mental health education in the field of colleges and universities covers all aspects of college students’ learning and life, and has made milestones. However, after finishing the research, it is found that although the research content is relatively rich, there are still research gaps in many details, mainly for the following reasons: firstly, the more common research method in the field of mental health education is the questionnaire survey method, and the case study method, interview method, discussion analysis method, situational experience method and literature research method are rarely used for inductive analysis; secondly, most of the literature treats the mental health education of college students as a subsidiary part of ideological and political education. Secondly, most of the literature treats the mental health education of college students as a subsidiary content of ideological and political education, but if the two are treated as two independent work contents of colleges and universities, the synergy between them is not obvious.

**Conclusions:** Mental Health Education and Curriculum Civics have become a social concern and have achieved some results, but we need to build on this foundation to further develop mental health education and ideological and political education in our universities.

**Acknowledgments**

This work was to thank my master’s supervisor for his guidance and encouragement in my work and writing.

## LHMM020 A STUDY ON THE EFFECT OF SPORTS GAMES ON CREATIVE THINKING INTERVENTION FOR CHILDREN WITH MILD SPEECH DISABILITIES

Xiuhai Shang^a^

^*a*^ *Department of Physical Education, Changzhou Vocational Institute of Industry Technology, Changzhou, 213000, Jiangsu, China*

**Background:** Sports game is a modern game that takes physical exercise as the basic method to enhance physical fitness, entertain body and mind and cultivate emotion under certain rules. It is a sports activity with obvious educational significance, which promotes the combination of physical development, intellectual development and physical and mental entertainment, and is not only a part of the game, but also closely related to sports. Sports games are used as an intervention to treat the creative thinking of children with speech disabilities, intervene to guide and stimulate the child’s interest, and allow the child to actively receive training in fluency, refinement, originality, resistance to premature closure, and abstraction of the title in a happy and enjoyable game.

**Subjects and Methods:** To explore the effect of sports games on the creative thinking of children with mild speech disabilities, and to provide references for promoting the development of creative thinking of children with speech disabilities. 26 children with mild speech disabilities aged 7-12 years from a special school in Nantong were randomly selected, and they were randomly divided into an intervention group (13) and a control group (13). The intervention group underwent a 12-week sports game intervention, and the control group maintained the original teaching arrangement. Before and after the intervention, the Torrance Creative Thinking Test was used to test the two groups of children. The total scores, fluency scores, anti-premature closure scores, originality scores, progress scores, the difference in the abstract score of the title.

**Results:** Before the intervention, there was no significant difference in the scores of the intervention group and the control group (P>0.05); after the intervention, the scores of the intervention group and the control group were significantly different (P<0.05); the intervention group in the experimental group In the comparison of scores in all aspects before and after intervention, there was no significant difference in diligence and abstractness of title (P>0.05), while the other four aspects had significant differences (P<0.05); in the control group, before and after intervention In the comparison of scores in all aspects, only the diligence and the total score were significantly different (P<0.05), and there was no significant difference in the other four aspects (P>0.05).

**Conclusions:** Sports games can promote brain development and improve the creative thinking of children with mild speech disabilities.

## LHMM021 MEASURES FOR BLOOD STATIONS TO REASONABLY AVOID LIABILITY FOR MEDICAL DAMAGE FROM BLOOD TRANSFUSION INFECTION IN THE ERA OF CIVIL CODE

Xingxiang Xing^a^

^*a*^ *Central Blood Station of Shaoxing City, Zhejiang 312000, China*

**Background:** The process of blood transfusion may lead to infection with pathogens such as human immunodeficiency virus (HIV), hepatitis B virus (HBV), and hepatitis C virus (HCV). The resulting medical damage cannot be completely avoided and often leads to medical disputes. In 2021, the Civil Code (the “People’s Republic of China” is omitted from the front part of the title of the law in this paper) was officially implemented, and relevant regulations and judicial interpretations were issued successively. The civil liability of blood collection and supply institutions such as blood stations was further clarified, and the legislation on blood transfusion entered the era of the Civil Code.

**Subjects and Methods:** The prevention of transfusion-infected medical damages is an important duty of blood stations, which are currently faced with technical deficiencies, inadequate legislation, and remedies that need to be improved. This paper analyzes the legal ecology of blood collection institutions in the civil code era, and concludes that the legislative environment for blood stations has changed in the civil code era, and that there are significant differences in the principles of imputation used by courts in the past to hear lawsuits for medical damages for blood transfusion infections. The paper also analyzes the legal logic of blood stations’ liability for transfusion-infected medical damages, and concludes that blood stations have a duty of care to avoid transfusion-infected medical damages.

**Results:** The legal perspective of blood stations to reasonably avoid medical damage liability for blood transfusion infection is proposed, including studying the law, knowing the practice guidelines; abiding by the law to achieve the correct practice; and being familiar with the current state of legislation and trial process; reasonable use of litigation skills.

**Conclusions:** In the era of the civil code, blood stations should reasonably avoid the responsibility of medical damage from blood transfusion infection. Based on the analysis of the legal ecology and legal logic of transfusion infection medical damage in the era of civil code, this paper proposes countermeasures for blood stations to reasonably avoid the liability of transfusion infection medical damage from a legal perspective, to ensure that blood stations can avoid the occurrence of transfusion infection medical damage to the greatest extent based on the safety of clinical medical blood supply.

## LHMM022 HEALTH RISK ASSESSMENT ON BLAST SIMULATION MODEL OF PETROCHEMICAL ENGINEERING BASED ON THE WORST POINT

Wenxu Yang^a,b^, Nana Zhang^c,d^

^*a*^ *School of Civil Engineering, University Sains Malaysia, Pulau Pinang, Malaysia,* ^*b*^ *Chongqing Dadukou District Department of Housing and Urban Rural Development, Chongqing City, China,* ^*c*^ *School of Communication, University Sains Malaysia, Pulau Pinang, Malaysia,* ^*d*^ *South Sichuan preschool education college, Longchang City, Sichuan province, China*

**Background:** Based on the traditional research method of safety risk of petrochemical industry in China, the concept of safety capacity is not fully embodied: the principle of the most disadvantageous point was not considered in most cases including personnel health. The damage of shock wave can be measured by three characteristic parameters: Peak overpressure, duration and impulse. According to the overpressure criterion, as long as the shock wave overpressure reaches a certain value, it will cause certain damage to personnel with a variety of adverse effects. Therefore, the study was conducted to determine the level of exposure and assess the quantitative health risk of exposure to some typical tank and gas explosion.

**Subjects and Methods:** Use PHAST software to evaluate the worst case for the explosion risk of Yangpu Economic Development Zone petrochemical enterprises in Hainan based on different explosion mode. The main hazard sources in the zone were analyzed after actual investigation, then two typical cases were selected: Huizhi Petrochemical 2000 cubic propane storage tank and 160000m3 CNOOC natural gas. The fire and explosion accident consequence simulation was carried out regarding on the process and damage area of the explosion accident to the surrounding environment under various explosion modes and different leak diameters. Taken as a whole, consolidated action is required to address these challenges and to ensure the protection of human health and prevention of diseases caused by exposure to chemicals.

**Results:** (1) the radius of death and serious injury increased with the increase of leak diameters; (2) the death radius (critical injury radius) decreases with the decrease of the leakage concentration and tends to be linear; (3) at the same leakage concentration, the death radius of ME explosion is larger than that of BST explosion, and the vapor cloud explosion mode is larger than that of BLEVE mode.

**Conclusions:** Starting from the most disadvantageous point, using Phast software to simulate petrochemical explosion, the ME explosion mode should be the first choice under the condition of whole rupture and maximum leakage, so as to fully consider the safety capacity of petrochemical engineering. Considering that the health risk of exposure to selected explosion situation was more than the standard regulated by some internal codes, taking engineering and management measures to control and reduce the risk levels to an acceptable level, including modification of local and general ventilation systems, as one of the main reasons for the respiratory exposure of the workers, is essential for the improvement of the situation.

## LHMM023 TARGETS AND STRATEGIES FOR FREE VACCINES AND THERAPEUTIC MEDICINE AGAINST THE PUBLIC HEALTH EMERGENCY

Fang Chen^a,b^, Hu Liu^c^, Chao Feng^b^

^*a*^ *Department of Economics and Management, Zhejiang Ocean University, Zhoushan, Zhejiang 316022, China,* ^*b*^ *Department of Mechanical and Electrical Engineering, Xinjiang Institute of Technology, Akesu, Xinjiang 843100, China,* ^*c*^ *Department of Naval Architecture and Maritime, Zhejiang Ocean University, Zhoushan, Zhejiang 316022, China*

**Background:** The public health emergency that has spread around the world has had a huge impact on people’s lives, economy and social development. How to quickly and effectively respond to emergency public health crises and reduce losses has become a hot topic for the national governments. Among all the ways and means, vaccines are thought to be the most economical and effective measure to prevent and control the infectious diseases.

**Subjects and Methods:** Firstly, the SIR model is applied to explorers how different vaccine efficacy affect the vaccination rate for herd immunity; Secondly, we assume that a free vaccine and therapeutic medicine strategy is put forward, we want to know the tendency to change government’s financial support or subsides based on the vaccination coverage. Thirdly, we assume the total social welfare or surplus is consist of two parts: the vaccinated’s and the unvaccinated’s, considering the negative network effects which could stimulate the potential people to free ride off the immunity of the vaccinated population. The relationship between the total social welfare/surpluses and vaccination coverage is also preliminarily explored.

**Results:** The vaccination rate for herd immunity(F) varies by the different vaccines of COVID-19, which have been made by Pfizer-BioNTech, Moderna, AstraZeneca-University of Oxford, Johnson & Johnson, Russia’s Sputnik V, Sinovac Biotech, Novavax, Bharat Biotech, usually is greater than 79.58%. When the vaccine efficacy is greater than or equal to 0.756, F is between 76.58%-96.92%. Otherwise, F is more than 1. This means that vaccination alone can not completely eradicate the epidemic; When vaccination rate is below the one for herd-immunity, the government subsides significantly decrease with increasing vaccination rate. Otherwise, the government subsides slightly increases. When vaccination rate is below the one for herd-immunity, the total social surplus increase with the vaccination rate. Otherwise, the total social surplus remains constant.

**Conclusions:** It is crucial for the government to take more effective efforts to improve the population’s confidence, improve vaccination willingness, and reduce vaccine hesitancy. Therefore, some advises of increasing vaccination rate are proposed: lower the cost of vaccine as much as possible, improve the convenience of vaccination, disclosure of vaccine information.

**Acknowledgements:** This work was supported by a project grant from Xinjiang social science funds (Grant No.20BGL088).

## LHMM024 A RECOMMENDATION METHOD OF HYGIENIC RESEARCH FRONTIER BASED ON TIME RELAXATION COEFFICIENT AND NEWS REPORTS

Yan Ma^a^, Lida Zou^b^, Yingkun Han^a^, Lei Ma^a^

^*a*^ *State Grid Shandong Electric Power Research Institute, Jinan 250002, China,* ^*b*^ *School of Computer Science and Technology, Shandong University of Finance and Economics, Jinan 250014, China*

**Background:** With the diverse and interdisciplinary development of different fields, researching topics are expanding rapidly. Tracing the newest hygienic research frontier is becoming more and more difficult. Meantime, data sources are various and heterogeneous. Besides massive academic literatures, news reports are effective supplements for scholars to follow up the research hotspots and frontier trends. This situation induces the information overload and research difficulty.

**Subjects and Methods:** Recommendation method could reduce information overload for scientific workers. Many works focus on academic literatures. With the rapid increase of various data on the Internet, recommendation method based on many kinds of data is required. In the paper, we propose a recommendation method based on news reports to help scientific workers find the time dependent topics related with their concerned hygienic field. Our method falls into four modules including the module of tracing hot news, relevant topic generation module, dependency generation module and the module of recommending frontier trends. We use time relaxation coefficient to introduce time sequence, which could find the targeted information exactly and eliminate the redundant data. The condition of choosing dependent topic word imports the parameter of page views as well to improve the excellent rate.

**Results:** We crawl and download the articles in a period and generate topic words using the proposed algorithm and two benchmark algorithms called Threshold Correlation (TC) algorithm and Greedy Dependency (GD). Experimental results demonstrate that our proposed method can exactly generate the objective topic words and has less redundant information. The recommendation algorithm also has averagely 62.5% in excellent rate and 72.2% in repetitive rate with manual generation algorithm.

**Conclusions:** Our work aims to recommend hygienic research frontier based on news reports for academic scholars. It considers the influence of news reports and time sequence. We introduce the parameter of time relaxation coefficient. When choosing dependent topic word, the parameter of page views is introduced as well. Experiments shows that the proposed recommendation algorithm could reduce the redundant information and improve the researching efficiency of scientific workers, while the benchmark algorithm recommends some redundant topics. Especially when the number of subscribed topics are large, our method performs well in excellent rate.

**Acknowledgments**

This research was supported by a project grant from State Grid (Grant No. 520626220042) and the Scientific Project of State Grid Shandong Electric Power Research Institute (Grant No. ZY-2022-07).

## LHMM025 THE REALIZATION OF THE VALUE OF PSYCHOLOGICAL EDUCATION IN UNIVERSITIES IN THE NEW ERA

Tao Xiao^a,b^, Yongjun Han^a^

^*a*^ *Institute of Industrial Development and Governance Innovation, Zhejiang Guangsha Vocational and Technical University of Construction, Jinhua 322103, Zhejiang, China,* ^*b*^ *School of Education and Humanities, Universiti Tun Abdul Razak, Kuala Lumpur 50400, Kuala Lumpur, Malaysia*

**Background:** To investigate the actual situation of psychological education, to enhance the value of psychological education in universities in the new era and solve the problems existing in the current psychological education in universities, such as the problems of the

integration of ideological and political education in universities,

the developing problems of great morality, the public morality, and the private morality to improve people’s moral standards and civilization and to establish an education system with socialist values with Chinese characteristics.

**Subjects and Methods:** A total of over 1,000 students and their stakeholders from May 2019 to April 2021 in some universities were enrolled in the study by the ways of questionnaire surveys, expert interviews, and individual conversation to master as much as possible the actual situation of psychological education. They were randomized into observation group and control group, each with over 500 cases. Both groups received routine management, including guidance, etc. Through data analysis and theoretical reconstruction, the value realization problem of university students’ psychological education in China is needed to be solved by using multiple educational paths.

**Results:** In the guidance of professional theoretical knowledge and practical experience, the working mode and content of psychological education are also constantly innovating and deepening, and certain effects have been achieved in the process of psychological education. By improving the professional ability and personality charm of psychological education teachers, universities narrow the distance between teachers and students, exert a subtle influence on students, and then realize the value of psychological education to set up a modern education system with special socialist values with Chinese characteristics.

**Conclusions:** Psychological education itself can help universities form a “three complete” education model, improve the mental health of college students, and at the same time, psychological education in universities has a clear and definite goal of education. To carry out psychological education in universities, we must make rational use of modern information technology, further innovate the means of psychological education, realize the psychological education mechanism of “three guarantees” education, and provide rich psychological health guidance for college students to fulfill a modern education system with Chinese socialist values with Chinese characteristics.

## LHMM026 DEVELOPMENT OF CORE COMPETENCE ASSESSMENT SCALE FOR NURSING UNDERGRADUATES BASED ON MENTAL HEALTH QUALITY

Keqing Chen^a,b,c,d^, Xiaoling Lin^e^, Wei Li^a,b,c,d^, Yanjuan Lin^f,g^

^*a*^ *Department of Thoracic Surgery, Union Hospital, Fujian Medical University, Fuzhou, China,* ^*b*^ *Fujian Medical University Union Hospital Department of Surgical Nursing, Fujian, China,* ^*c*^ *Key Laboratory of Gastrointestinal Cancer, Fujian Medical University, Ministry of Education, Fuzhou, China,* ^*d*^ *Fujian Key Laboratory of Tumor Microbiology, Fujian Medical University, Fuzhou, China,* ^*e*^ *Zhongshan Hospital Xiamen University, Xiamen, China,* ^*f*^ *Department of Nursing, Union Hospital, Fujian Medical University, Fuzhou, China,* ^*g*^ *Department of Cardiac Surgery, Union Hospital, Fujian Medical University, Fuzhou, China.KC, XL and WL contributed equally to this work.*

**Background:** People’s life and health problems have always been the focus of people’s attention. In order to provide a scientific evaluation tool for the evaluation of the core competence of undergraduate nursing students. The core competence Scale for nursing undergraduates was developed based on mental health quality by Delphi method, which provided a theoretical basis for clinical improvement of teaching methods.

**Subjects and Methods:** This paper aims to develop a core competence assessment scale based on mental health literacy for nursing undergraduates, and test its reliability and validity, so as to provide a basis for the improvement of clinical teaching methods. The core competence of undergraduate nursing students refers to the basic competence necessary for clinical treatment, health education, communication and other nursing work. Based on the existing core competence scale of nurses, the evaluation index of core competence based on mental health literacy of nursing undergraduates was preliminarily determined through literature review, and two rounds of expert correspondence courses were conducted by Delphi method. The multi-objective decision analysis technology of developing the core competence prediction scale for nursing undergraduates, and conducting reliability and validity tests to form a formal scale, and conducting scientific processing, not only ensures the rationality of the research results, but also ensures its objectivity.

**Results:** The developed scale contained 8 primary indicators such as mental health related traits, mental health related performance, and 29 secondary indicators. The recall rates of the two rounds of expert letter questionnaires were 100% and 91%; the expert authority coefficients were 0.74 and 0.78; the mean importance value of expert consultation entries was greater than 0.35, the standard deviation was less than 0.1 and the coefficient of variation was less than 0.25; the clonbach coefficient of the questionnaire was 0.950; and the questionnaire validity index (KMO) was 0.911 (all P < 0.01).

**Conclusions:** The core competence assessment scale of nursing students compiled in this study shows that experts have good enthusiasm, concentration and coordination through statistical analysis, which can be used as an assessment tool to measure the mental health quality and core competence of nursing students and provide a basis for guiding clinical instructors to cultivate the mental health quality and core competence of nursing students.

**Acknowledgments**

This research was supported by the Teaching Reform Project of Fujian Medical University (J21046).

## LHMM027 RESEARCH ON THE MENTAL HEALTH EDUCATION MECHANISM OF POSTGRADUATES IN NAVIGATION UNIVERSITIES

Tianshu Wang^a^, Mingyu Wang^a^, Jing Zhou^a^, Mingming Sun^a^

^*a*^ *Naval Architecture and Ocean Engineering, Shandong Jiaotong University, Jinan, Shandong, 250357, China.*

**Background:** To explore the mental health problems and existing obstacles of postgraduates, and summarize the experience according to the actual work.

**Subjects and Methods:** A total of 82 navigation postgraduate students (Grade 20–22) from School of Shipping, Shandong Jiaotong University were selected as the research objects. This paper aims at cultivating positive psychological quality and psychological health education. Based on the questionnaire, psychological test and salon talk, the present situation and obstacles of graduate students’ mental health were observed and analyzed. Then, a questionnaire survey and research were conducted with the teacher and the students’ parents to analyze the relationship between graduate student’s mental health and the teacher and family.

**Results:** Postgraduate is one of the most critical periods in one’ s life, and it is also an important period to establish its world outlook, outlook on life and values. This article discovered that the expansion of graduate enrollment, scientific research, employment, family and society have brought heavy psychological pressure on graduate students. Postgraduates with possible psychological problems account for 15% of the survey population, and those with obvious psychological problems account for 8%. Some postgraduates also have such worrying mental problems as mania, inferiority complex and depression. Although many graduate students are affected by psychological confusion and mental health problems, only a few actively seek help with psychological problems. At present, mental health education for graduate students only focuses on the prominent parts, trying to solve one problem after another, while ignoring the importance of mental health education skills in graduate students’ daily life and scientific research work.

**Conclusions:** Therefore, we need to combine the present situation and existing obstacles of postgraduates’ mental health, incorporate mental health education into the graduate training plan, construct a new management education system for graduate students, strengthen postgraduates’ mental health education and provide professional psychological counseling services. Give full play to the educational function of campus culture and practical activities, give full play to the educational function of culture, effectively improve the quality of postgraduate mental health education, and promote the all-round development of graduate students in colleges and universities.

**Acknowledgments**

This research was supported by Shandong Jiaotong University Graduate Education Teaching Reform Research Project “Research on the Construction of ‘U-S-F’ Training Management Mechanism for Professional Degree Postgraduates from the Perspective of Integration of Industry and Education” (JYY 202223); “Ship and Marine Engineering,” a demonstration base for the joint training of production and education integrated postgraduate students in Shandong Province; Research Project of Teaching Reform in Shandong Jiaotong University “Teaching Reform and Exploration of ‘Mechanical and Electrical Products Innovative Design’ Course Based on Results under the Background of New Engineering” (2022YB71); Doctor Start-up Fund of Shandong Jiaotong University “Research on Shipping Economy under the Double Carbon Target” (BS2021030); Shandong Province Transportation Science and Technology Project(2021B115); Shandong Jiaotong University School Fund (Z2019036).

## LHMM028 A STUDY ON THE IMPACT OF TOTAL FACTOR PRODUCTIVITY OF ENVIRONMENTAL SERVICE FIRMS ON ENVIRONMENTAL AND HEALTH DEVELOPMENT IN CHINA: BASED ON NON-EXPECTATION MINDS SUPER-EFFICIENT METAFRONTIERMALMQUIST MODEL

Yijun Zhou^a^, Meixian Peng^b^, Yi Luo^c^

^*a*^ *School of Finance and Economics, Hunan University of Technology and Business, Changsha, 410205, China,* ^*b*^ *Chongqing Jiulongpo Hospital of Traditional Chinese Medicine, Chongqing, China,* ^*c*^ *Attending Physician, The First Affiliated Hospital of Chongqing Medical University, Branch 1, Chongqing, China*

**Background:** A good ecological environment is the basis of human survival and health. The degree of environmental health directly influences the degree of mental health of an individual. Environmental health has been shown to have an extremely close relationship with mental health. With the development of industrialization and urbanization, air, water and sudden environmental pollution have become important risk factors affecting the health of residents, and the relationship between environment and health has become a hot issue.

**Subjects and Methods:** The cultivation of environmental service enterprises has risen to a national strategic level, and the scientific measurement of total factor productivity of environmental service enterprises can accurately grasp their evolutionary trends, driving factors and optimization paths between environment and health, which has certain theoretical value and practical significance. Based on the data of 253 environmental service enterprises in 14 categories from 2010 to 2020, this paper measures the total factor productivity of 253 environmental service enterprises in 14 categories using the non-expectation MinDS super-efficiency-MetaFrontier-Malmquist model, and decomposes the total factor productivity of environmental service enterprises by Malmquist index.

**Results:** The results of the study show that the total factor productivity of environmental service enterprises in China is generally not high and their efficiency does not reach DEA effective; among the 14 major categories of environmental service enterprises, construction environmental service enterprises have the highest total factor productivity efficiency value, while the lowest efficiency values are in real estate and electricity and heat production and supply environmental service enterprises.

**Conclusions:** Among the environmental service firms in the ecological protection and environmental management category, which is the focus of this paper in particular, 69% of the firms in this category have a technical efficiency greater than 1, and only 46% of the environmental service firms in this category have a technical progress efficiency greater than 1. This further suggests that environmental service firms have a significant positive relationship to environmental and health development in China.

**Acknowledgements**: The successful writing of this paper is thanks to the support of my supervisor and fellow students, siblings and juniors.

## LHMM029 EFFECT OF NEGATIVE PRESSURE TECHNIQUE IN ORAL RESTORATION ON THE BONDING STRENGTH BETWEEN ZIRCONIA CERAMIC AND METAL ORTHODONTIC BRACKETS

Zhao Wei^a^, Pei Wang^a^, Lin Ren^a^, Xue Yu^a^, Xuejuan Ma^a^, Yongping Ma^a^

^*a*^ *The NO.2 Hospital of Baoding, Baoding 071000, HeBei, China*

**Objective:** To study the influence of negative pressure technology on the bonding strength between zirconia ceramic and metal orthodontic brackets.

**Method:** 48 zirconia ceramic specimens were randomly divided into 6 groups (n=8): Group A directly used TransbondTMXT bonded the metal bracket with the zirconia specimen, and group B used Transbond after sandblasting of the zirconia specimenTMXT was bonded and the Group C sandblasted specimens were coated with TransbondTMXT gave 0.04MPa negative pressure treatment after the bonding of the bracket, Group D bonded the specimens using Clearfil SAC, Group E specimens for tank bonding with Clearfil SAC, Group F sand blasting treated specimen coating Clearfil SAC given 0.04MPa negative pressure treatment for adhesion of the bracket each group of randomly selected 1 specimen using the scanning electron microscope (SEM) to observe the specimen surface morphology of all specimens for bracket adhesion after shear strength test (SBS), The binder residual index (ARI) was observed by body type microscope. Statistical analysis was performed using the SPSS 26.0 software, Experimental data of shear adhesive strength were tested for normality and homogeneity of variance, Statistical analysis was then performed on the experimental data and ARI test data using Kruskal-Wallis H, Multiple comparison between groups using Bonferroni.

**Result:** SEM shows a clear rough surface, The zirconia surface treated with negative pressure, More uniform adhesive distribution The SBS results showed that the SBS values in Group F (8.28 ± 3.08) and Group C (6.62 ± 1.85) were significantly higher than the other groups, And showed a statistically significant difference (P <0.05), And Group B and Group C showed significantly higher SBS values compared with Group A, And a statistical difference (P <0.05), Group E showed significantly higher SBS values in group F compared to group D, And there is a statistical difference (P <0.05) group E (6.54 ± 2.47) and group F (8.28 ± 3.08) but not statistically significant (P> 0.05) the highest ARI index F group but no significant difference between the groups (P> 0.05).

**Conclusion:** negative pressure technology can significantly improve the bonding strength between zirconia ceramics and metal orthodontic brackets, better combined with sandblasting and MDP.

## LHMM030 BARRIERS AND FACILITATORS IN INTRODUCING ARTIFICIAL INTELLIGENCE INNOVATIONS INTO RADIOLOGY PRACTICE: A SYSTEMATIC SCOPING REVIEW

Fatma A. Eltawil^a^, Emily Boulos^a^, Afsaneh Amirabadi^b^, Pascal N. Tyrrell^a,c,d^

^*a*^ *Department of Medical Imaging, University of Toronto, Toronto, Ontario, Canada,* ^*b*^ *Diagnostic Imaging Department, The Hospital for Sick Children, Toronto, Ontario, Canada,* ^*c*^ *Department of Statistical Sciences, University of Toronto, Toronto, Ontario, Canada,* ^*d*^ *Institute of Medical Science, University of Toronto, Toronto, Ontario, Canada.*

**Background:** The incorporation of Artificial Intelligence/ Machine learning into radiology practice is inevitable. This systematic scoping review was conducted to determine the barriers and facilitators for the introduction of artificial intelligence/machine learning (AI/ML) enabled innovations into radiology practice.

**Subjects and Methods:** A systematic search was performed using Ovid Medline and Embase. Keywords were used to generate refined queries with inclusion criteria for computer-aided diagnosis, artificial intelligence, and barriers and facilitators. Two reviewers assessed the articles, with a third reviewer for disagreement. The risk of bias was mitigated by including both quantitative and qualitative studies. An electronic search from January 2000 to 2022 identified 489 studies. Only 6 articles were found to fulfill the inclusion criteria: qualitative studies (n = 3), survey studies (n= 2), RCT (n = 1).

**Results:** Among the most common barriers to AI implementation into radiology practice were: lack of acceptance and trust of the radiologists; lack of awareness, knowledge, and familiarity with the technology; and perceived threat to the professional autonomy of radiologists. The most important identified AI implementation facilitators were the following: high expectations of AI’s potential added value, potential to decrease errors in diagnosis, and potential to improve the quality of patient care.

**Conclusions:** This scoping review found that there are very few studies designed specifically to identify barriers and facilitators to the introduction of AI to radiology practice. Providing further research evidence on barriers and facilitators to the introduction of AI will allow for improved assessment and adoption of these technologies into radiology practice in the future.

## LHMM031 RESEARCH ON THE MECHANISM AND PATH OF PROMOTING MASS SPORTS IN DAILY LIFE BY THE DEVELOPMENT OF FITNESS YOUNG IN NORTHERN SHAANXI FROM THE PERSPECTIVE OF EMOTION REGULATION

Mingyou Gao^a^, Chao Wang^b^, Sidan Xu^c^, Dandan Yang^a^, Yongqiang Du^d^, Shuai Zhu^a^

^*a*^*.Yulin University, Yulin, Shan’ xi Province, 719000, China,* ^*b.*^ *Yulin Vocational and Technical College, Yulin 719000, China,* ^*c.*^ *Shaanxi Preschool Normal College, Xi ’an 710100, China,* ^*d.*^*Yulin High-tech Zone No. 1 Middle School, Yulin 719000, China.*

**Background:** By using regression analysis, structural equation model and literature method, this paper analyzes the relationship between transformation ability and transformation performance of fitness Yangko development in northern Shaanxi, which is helpful to the activation and transformation of mass sports students. On the basis of analyzing the theory and logic of the development process of fitness Yangko in northern Shaanxi, this paper discusses the mechanism and path promoting mass sports life. It is considered that the application of various combined movements and a large amount of body language in the fitness Yangko can vent bad emotions in time through conscious movements, expressions and body language, and make people feel high and healthy in a relaxed and beautiful melody. Such as relieving anxiety, releasing stress, stabilizing mood, psychological balance, relieving depression, and enjoying body and mind. The main conclusions are as follows: in the new era, the dynamic mechanism of Yangko fitness to help people’s sports life should strengthen the coordination mechanism led by the government, the incentive mechanism of suppliers, the guarantee mechanism of resource integration and the result-oriented supervision mechanism, so as to realize the same direction to help people’s sports life. On the path of action, it is suggested that the government deepen its support and preference for fitness Yangko in policy, that the society create a good environment and help people live a sports life, that schools provide popularization and improvement of fitness Yangko courses, and that enterprises provide services and supplies for fitness Yangko performances and activities to achieve mutual benefit and win-win.

This research is based on the new era of fitness Yangko in northern Shaanxi to help the development of mass sports in life, which is in line with the requirements of the national strategy of fitness for all. It is a specific action to widely carry out fitness for all activities, accelerate the construction of a sports power, and form a new style of mass sports that is upward and good for the whole society. In the research, based on the analysis of the theory and development process logic of the fitness Yangko in northern Shaanxi to help the mass sports in life, the mechanism and action path of the fitness Yangko in northern Shaanxi to help the mass sports in life were explored.

**Subjects and Methods:** Regression analysis, structural equation model and literature method, this paper analyzes the transformation ability and transformation performance of the development of fitness Yangko in northern Shaanxi, which helps to transform mass sports into life. It is proposed to put forward hypothesis and verify it from six aspects: building fitness Yangko facilities, organizing fitness Yangko activities, holding fitness Yangko competitions, providing fitness Yangko guidance, and telling good fitness Yangko stories.

**Results:** The dynamic mechanism of fitness Yangko in northern Shaanxi in the new era to help people to live a life of sports should strengthen the overall planning mechanism led by the government, the incentive mechanism of the main supplier, the guarantee mechanism of resource integration, and the effect oriented supervision mechanism, and implement the same direction to achieve fitness Yangko to help people to live a life of sports. In terms of the action path, we should accurately grasp the multi subject participants, and suggest that the government should deepen its support and preference in policy.

**Conclusions:** The community should create a favorable environment for fitness Yangko to help people to live a life of sports, schools should provide the popularization and improvement of fitness Yangko courses, and enterprises should provide services and supplies needed for fitness Yangko performances and activities to achieve mutual benefit and win-win results.

**Acknowledgements**

This work was supported by the Shaanxi Social Science Fund project in 2021 (project number: 2021Q007); and Project of Shaanxi Yulin Science and Technology Bureau in 2021 (Project No.: CXY-2021-99-01).

## LHMM032 STUDY ON PHYSICAL DAMAGE ASSESSMENT OF ROADWAY WALL AND PSYCHOLOGICAL INJURY BY GAS EXPLOSION

Zhenzhen Jia^a^, Qing Ye^a^, Yangzhou Teng^a^

^*a*^ *School of Resource, Environment and Safety Engineering, Hunan University of Science and Technology, Xiangtan 411201, China.*

**Background:** Gas explosion is a serious disaster accident in coal mine, which brings psychological and physical damage of miners. At present, there are few methods to assess the physical damage of roadway wall and psychological injury of miner during gas explosion and its propagation process.

**Subjects and Methods:** In order to study the damage caused by gas explosion and obtain the damage evaluation method, in this paper, LS-DYNA numerical simulation software is used to establish the mathematical model and physical model of damage of the roadway wall, some reasonable assumptions are made for the physical model. At same time, the model is properly divided by Euler grid for gas (air), and Lagrange grid for roadway wall. The unit length is controlled to be 0.05m, so the model is divided into 171700 units. The corresponding explosion conditions are set. Finally, numerical simulations of the dynamic response under gas explosion impact load are carried out; the overpressure and impulse during gas explosion propagation with different explosion intensities are measured.

**Results:** Damage degrees of roadway wall under different gas explosion intensities are divided into the mild damage field, moderate damage field, severe damage field, serious damage field and collapse damage field by taking bearing angles 20, 40, 60 and 80 as nodes. The overpressure-impulse damage criterion based on bearing angles is adopted to evaluate the damage degrees of roadway wall element. The boundary points of different damage degrees are obtained through numerical simulation, and the boundary points are connected to plot the P-I curve of roadway wall under the gas explosion impact. Finally, the formula for fitting the P-I curve of roadway wall is obtained.

**Conclusions:** The research results show that the established P-I curve and the damage degree of roadway wall can reflect the dynamic response of roadway wall. The damage assessment is basically reasonable and can be used for the evaluation of underground disaster by gas explosion. Also according to human injury criteria of the overpressure and impulse of shock wave, we can determine the injury degree of miners and damage range by gas explosions. The research results can provide a reference for the damage evaluation of roadway wall caused by gas explosion.

**Acknowledgments**

This work is financially supported by the National Natural Science Foundation Project of China (52174177, 52174178) and project supported by Scientific Research Fund of Hunan Provincial Education Department (20B240). Those supports are greatly acknowledged.

## LHMM033 ADOLESCENT PHYSICAL LITERACY QUESTIONNAIRE FOR CHINESE ADOLESCENT: VALIDITY AND RELIABILITY STUDY

Qian Song^a^; Xuebin Wang^b^

^*a*^ *School of Marxism Studies, Shanghai University of Sport, Shanghai 200438, China,* ^*b*^ *Department of Physical Education, Shanghai Jiao Tong University, Shanghai 200240, China.*

**Background:** Physical literacy is instrumental to developing lifelong physical activity habits. Over the last few years, countries from all over the world have become increasingly concerned about the development of physical literacy. A scientific evaluation system is essential to promote the development of physical literacy among youth. The Mahmoud Sheikh Iranian Adolescent Physical Literacy Questionnaire provides a great measure of adolescents’ physical literacy because of its simple operation, convenience, and effectiveness. This study aimed to examine the validity and reliability of the Iranian Adolescent Physical Literacy Questionnaire among Chinese children aged 11 to 15.

**Subjects and Methods:** The criterion sampling method was one of the purposeful sampling methods used in the study. There were a total 611 primary school students (135 boys, 52.2%;147 girls, 47.8%, mean age 13) in grades 5 to 8 who participated in the research. A forward and reverse translation method is used to determine language validity. Test results showed a highly positive correlation between the scales of two language forms (r =.98, p<.01). The construct validity was tested using AFA and CFA. To determine the relationship between factors, Pearson Correlation Analysis was used. In order to determine item discrimination, item test correlation was calculated as well as a 27% comparison between lower and upper groups. Analyses of convergence and divergence of validity were conducted. The unconstrained, measurement weights, structural covariance, and measurement residuals were used to measure the in-variance of questionnaires in physical literacy assessments for men and women. A reliability analysis was conducted using Cronbach’s Alpha and test-retest analysis. A Cronbach’s Alpha internal consistency coefficient of 0.98 was obtained for the overall questionnaire. The test-retest reliability (R1) of the overall questionnaire and each dimension is between 0.95 and 0.97, and there is a significant correlation.

**Results:** As a result of EFA, a structure with 4 factors and 21 items was determined. The results of the reliability analysis indicate that the measurement tool can be measured reliably.

**Conclusions:** Adolescent Physical Literacy Questionnaire can be used to assess Chinese adolescents’ physical literacy. Specialists working in the field of physical education and sports can provide the necessary support with appropriate intervention programs in line with the determinants of the physical literacy of adolescents.

**Acknowledgments**

This work was supported by a project grant from National Social Science Fund Youth Projects (Grant No.22CTY011), Shanghai Social Science Planning Youth Project (Grant No.2020ETY004), Shanghai Education Science Research Project (Second Batch) (Grant No.C2-2020012).

## LHMM034 EFFECTIVENESS OF NETWORK COLLEGE ENGLISH TEACHING ON RELIIEVING STUDENTS’ LEARNING PRESSURE AND PSYCHOLOGICAL ANXIETY: ACASE STUDY OF MIANYANG TEACHERS’ COLLEGE

Xia Zhang^a^

^*a*^ *Mianyang Teachers’ College, Mianyang 621000, Sichuan, China.*

**Background:** As one of the basic and compulsory courses of higher education, college English plays a very important part in students’ academic completion, the cultivation of comprehensive qualities, as well as the participation of international exchanges and cooperation. College English Curriculum Requirements released by Chinese Ministry of Education in 2007 which states, “Colleges and universities should remould the existing unitary teacher-centered pattern of language teaching by introducing computer- and classroom-based teaching modes. The new mode should be built on modern information technology, particularly network technology, so that English language teaching and learning will be, to a certain extent, free from the constraints of time or place and geared towards students’ individualized and autonomous learning.” Following this guideline, most universities employ network teaching means to quicken the pace of College English Teaching towards the scientific age of information and networking. Computer-oriented information technology gradually becomes the pivotal componential part of College English Teaching, which is correspondingly changing the teacher-student relationship.

**Subjects and Methods:** This study makes a comprehensive analysis on effectiveness of network college English management mode from three perspectives of students’ personal profile, personal behavior and teaching factors. Meanwhile, the study selected Mianyang Teachers’ College (MTC), Sichuan Province, China as the case study. The object in this study is the students and teacher. So the researcher sent the questionnaires to the English teachers of MTC and asked them to help to distribute the questionnaire to their students who are the fresh students of 11 schools of MTC. The researcher and her colleagues distributed 430 questionnaires and collected 428 valid ones.

**Results:** This study mainly uses quantitative method, through the questionnaire survey, it analyzes both teachers’ and students’ behavior and teaching factors, it tests three hypothesis.

**Conclusions:** Finally, it concludes MTC should construct this teaching management mode: “network teaching in class and network autonomous learning after class”: that is to make full of advantages of internet and build a good platform for students’ self- learning, personalized learning and collaborative learning, so as to improve effectiveness of network college English teaching management mode, also by this means can relieve students’ learning pressure and psychological anxiety.

## LHMM035 THE ESTABLISHMENT OF THE INDICATOR SYSTEM OF PUBLIC SAFETY GOVERNANCE IN URBAN COMMUNITIES AND ITS EFFECT ON ALLEVIATING RESIDENT’S ANXIETY AND INSECURITY

Yan Wang^a,b^, Fanchang Meng^a^, Yifan Liu^b^

^*a*^ *School of Public Policy & Management, China University of Mining and Technology, Xuzhou 221116, China,* ^*b*^ *School of Sociology, Beijing Normal University, Beijing 100875, China.*

**Background:** Against the backdrop of economic transition, social transformation and the rapid development of new-type urbanization, all kinds of public safety problems mount and concentrate on urban communities, causing anxiety and insecurity among community residents.

**Subjects and Methods:** In the context of the modernization of state governance and on the basis of the principles of “systematic, legal, comprehensive and original,” an evaluation indicator system of public safety governance in urban communities is preliminarily established in such eleven fields as child safety, elderly safety, home safety, safety in public space, school safety, sports safety, road traffic safety, water safety, workplace safety, hospital safety and university safety.

**Results:** Public safety governance in urban communities is an essential part of state governance and an important means to achieve public safety. Governance can directly influence the sense of safety and psychological health of community residents. Based on the establishment of the indicator system of public safety governance in urban communities, the theoretical framework of “resources-capacity-collaborative governance” is proposed and the coupling of these three is a necessary condition for good governance.

**Conclusions:** A good match among resources, capacity and collaborative governance is important to the influence mechanism and measurement of the level of public safety in urban communities. By this good match, targeted suggestions can be made to solve existing problems in community safety governance, resource utilization can be optimized, the level of public safety governance in urban communities can be improved, and a feeling of anxiety and insecurity among residents can be alleviated.

## LHMM036 FIRST PRINCIPLES STUDY OF OXYGEN VACANCIES DEFECTS IN RUTILE TIO2 AND APPLICATION IN PRECISION MEDICAL EQUIPMENT WITH BEHAVIORAL PSYCHOLOGY

Wei Niu^a^, Yingnan Dong^a^, Jian Tang^a^, Gang Wang^b^

^*a*^ *School of New Energy, Shenyang Institute of Engineering, Shenyang, 110136, China,* ^*b*^ *School of Electric Power, Shenyang Institute of Engineering, Shenyang, 110136, China*

**Background:** The application of high-quality rutile single crystals in precision medical equipment can greatly improve the accuracy of detection instruments, effectively improve the level of medical technology in many fields such as predisease inspection and health maintenance, and shorten the treatment time of patients. In this paper, a first principle model of oxygen vacancy defect in rutile TiO2 with behavioral and psychological effects is established and studied. In this paper, the fundamental reproducing number R0 is obtained by using the next generation generation matrix method, and the existence and stability of the equilibrium point are discussed.

**Subjects and Methods:** In this study, three experiments were conducted to explore the effects of psychological patterns and self correlation on the first sex principle. Experiment 1 used money and human eyeliner to start different psychological models of individuals, and examined the psychological behavior of the first principle in the market model and the public model through cockfighting games. In experiment 2, ERP technology was used to investigate the influence of psychological patterns on the synthetic behavior of first principles. Experiment 3 examined whether the First Principle had different behaviors in different synthetic ways under different psychological models. Behavioral psychology is one of the most influential psychological schools in the first half of the 20th century. Behavioral psychology was founded by American psychologist Watson. Rutile single crystals have a high refractive index, birefringence, and good optical transmittance in the visible-infrared band, which are widely used in precision medical equipment. Due to the high melting point and high chemical reactivity, rutile TiO2 tends to decompose to form a non stoichiometric compound TiO2-x at high temperatures in molten states, which leads to the existence of oxygen vacancies defects in rutile crystal. Oxygen vacancy defects significantly affect the optical and electrical properties of rutile. Rutile single crystals with large oxygen vacancies can greatly reduce the precision of medical equipment. The plane-wave ultrasoft pseudopotential method was used to study the energy band structure, electronic-state density distribution, and optical properties of rutile TiO2-x (x=0, 0.0625, 0.083, and 0.125) with different oxygen vacancy concentration in the work.

**Results:** The results show that the synthesis rate in common mode is significantly higher than that in control group and stress field mode. The P3 amplitude induced in the decision-making stage under the public mode is smaller than that of the market mode and the control group. In the result evaluation stage, under the public mode, there is no difference in the amplitude of FRN induced by receiving attack feedback and receiving cooperative feedback. Under the market mode and control group conditions, the amplitude of FRN induced by receiving aggressive feedback is significantly greater than that induced by receiving cooperative feedback. And the P300 amplitude induced in the public mode is significantly smaller than that induced in the market mode. In the result evaluation stage, the P300 amplitude induced by the synthesis result is greater than the loss result. When R0<1, the first principle model equilibrium point of the psychological effect on oxygen vacancy defects in rutile TiO2 is globally asymptotically stable. When R0>1, there is a unique equilibrium point of psychological effects and it is locally asymptotically stable. The unit cell volume of rutile TiO2-x increased as oxygen vacancy concentration increased, and three Ti adjacent to the oxygen vacancies moved away from oxygen vacancies. Oxygen vacancies caused the Fermi level of rutile TiO2-x to move up into the conduction band, and the state density on the top of the value band decreased. The state density increased at the bottom of the conduction band, where oxygen vacancy impurity levels formed. The oxygen-vacancy impurity energy level was mainly composed of Ti-3d states and a small amount of O-2p states, and the impurity energy level was located below the Fermi surface and occupied by electrons. The light absorption coefficient of rutile TiO2-x increased gradually with increased oxygen vacancy concentration. Rutile TiO2-x showed a yellow-blue-gray/black changing trend.

**Conclusions:** First principles will be effected by behavioral psychology. First principles study will provide a theoretical basis for rutile applications in precision medical equipment. Oxygen vacancy defects lead to the decline of stability, electrical properties and optical properties of rutile TiO2. The application of high-quality rutile single crystals in precision medical equipment will realize the effective improvement of medical detection and treatment level, provide technical support for disease inspection, and reduce misdiagnosis.

**Acknowledgements:** This work was supported by the central government guides the local government special fund (2022010057-JH6/1001), scientific research fund of Liaoning provincial department of education (JL-2010).

## LHMM037 RESEARCH ON THE DESIGN DIMENSION OF SMART PHONES BASED ON THE PSYCHOLOGICAL CHARACTERISTICS OF THE ELDERLY POPULATION

Zhaopo Shao^a^

^*a*^ *School of Fine Arts, Huanggang Normal University; Huanggang 438000, Hubei Province, China*

**Background:** In the context of the aggravation of aging in the information age, the design strategies for the smart phones based on the psychological characteristics of the elderly population were discussed, and the corresponding design concept and paths were summarized.

**Subjects and Methods:** One hundred elderly people aged 60 to 70 years old in Zhumingshan community of Huanggang City were taken as the objects to study the structural and functional degradation of their vision, hearing, perception, and touch as well as the changes of their life state, which resulted in a series of group psychological characteristics by means of observation, interview, and questionnaire. In particular, relevant data are acquired and analyzed for the elderly’s behavior habits, psychological description, on-site observation and personal experience when using the smartphone user interface, and the level and key problems in the current smartphone user design are comprehensively investigated and quantitatively evaluated and graded, so as to provide reference for the age-appropriate design of the smartphone user interface.In addition, the behaviors of the elderly such as reading novels and articles, socializing, watching short videos and shopping through mobile phone APPs were investigated and studied to master the behavioral characteristics and practical demands of them using smart phones.

**Results:** Smart phones, as an important carrier to support the intelligent old-age care embedded in the pension service system, are not only actively responding to the objective needs of the elderly population, but also improving their quality of life. Correspondingly, the innovative dimensions of smart phone design were proposed, including health care in individual internal dimension, emotion care in family and intergenerational interaction, and service care in social management.

**Conclusions:** As the three innovative design dimensions of smart phones based on the psychological characteristics of the elderly group are of positive significance to help the elderly integrate into the intelligent society and improve the quality of life in their later years, it is worthy of promotion. It is helpful to supplement the integrity of the research framework of “appropriate aging” for smart phone user design, and provide a theoretical basis for solving the problem of effective identification of smart phone user design for the elderly group.

**Acknowledgments**

Supported by the Discipline Construction Project of Huanggang Normal University “Research and Creative Practice of Regional Humanities and Arts”

## LHMM038 WHETHER FISCAL POLICY HAS IMPROVED NATIONAL MENTAL HEALTH BY PROMOTING HEALTH INDUSTRY DEVELOPMENT

Wei Li^a^, Zhiwei Liu^a^, Ke Li^a^

^*a*^ *School of Finance and Economics, Hunan University of Technology and Business, Changsha, Hunan 410205, China.*

**Background:** In recent years, there has been a large increase in China’s investment in primary medical institutions and population health, but more than half of China’s national mental health is in a subhealthy state, and the happiness index is in the middle and lower reaches of the world. The health industry can improve national mental health and physical quality, so it is necessary to improve national mental health and enhance national happiness through the development of health industry. The development of health industry needs the support of fiscal and taxation policies, and in 2014, China increased the support of fiscal and taxation policies for health industry in order to meet the growing and beautiful needs of people, improve national mental health and promote economic development.

**Subjects and Methods:** Based on the key node of strengthening fiscal and tax policies to develop health industry to enhance national mental health in 2014 as a background, analyze the construction of primary medical institutions and financial investment in medical care, population and health in China, make a basic judgment on the development status of China’s health industry, and analyze whether fiscal and tax policies have effectively improved the productivity of listed companies by combining the data of listed companies related to health industry. To infer the influence of fiscal and tax policies on the development of health industry, by studying the correlation between fiscal and tax policies and the development of health industry, in order to provide reference for optimizing fiscal and tax policies and more effectively inferring the development of health industry, including the construction of primary medical institutions and population and health investment, and then improving the national mental health and physical quality.

**Results:** After strengthening the fiscal and tax policies to support the health industry in 2014, the scale of primary medical institutions in China has increased significantly, the growth rate of health insurance has been obvious, and there has been a significant promotion effect on the development of the health industry, and the production efficiency of listed companies related to the health industry has been significantly improved. The fiscal and taxation policies mainly improve the production efficiency by expanding the scale of operation of health industry enterprises and promoting their R&D investment and fixed asset investment.

**Conclusions:** The development of the health industry is closely related to the improvement of national mental health. It is important to implement active fiscal policies to promote the development of the health industry, reduce the tax burden bundle of the health industry, enhance the financial guidance for the health industry, create a favorable environment for the development of the health industry, and promote the development of the health industry as a way to improve national happiness and mental health.

**Acknowledgments**

This research was supported by Study on Effectiveness Evaluation and Policy Optimization of Multi-dimensional Large-scale Tax Reduction to Promote High-quality Development of Manufacturing Industry (2021JJ30194).

## LHMM039 RESEARCH ON THE CONCEPT AND DIMENSION OF HEALTHY ORGANIZATIONS FROM A PUBLIC SECTOR PERSPECTIVE

Jing Hu^a^, Xuechen Li^a^

^*a*^ *Department of Public Security Management, Beijing Police College, Beijing 102202, China.*

**Background:** In recent years, “health” has become the focus of attention of individuals, society and the state. Since the 1990s, enterprises have paid more attention to employees’ health, especially their mental health. Both theory and practice have proved that work pressure has a certain impact on employees’ work attitude and behavior, and employees’ health is crucial to the development of enterprises. After the 21st century, the research on employee health has expanded from the individual level to the organizational level. Healthy organizations are receiving attention and focus from both theoretical and practical circles.

**Subjects and Methods:** Based on the review of the existing research at home and abroad, combined with the characteristics of the public sector in China and the characteristics of the times, the healthy organization was defined from the levels of individual, organization and environment. Through qualitative analysis, NVivo10.0 software was used to perform open coding, spindle coding and core coding on the interview scripts of 20 interviewees to discover the dimensions and specific contents of healthy organizations.

**Results:** The research results show that healthy organization covers three dimensions, namely, employee health, organizational health and social responsibility, among which employee health includes personal mental and physical health, organizational health includes organizational culture, organizational practice and organizational learning, and social responsibility assumes responsibility for the public, society and environment in the process of shaping the external image of the public sector.

**Conclusions:** At present, the research on healthy organization in academic circles is still in its infancy, and the solution of basic problems such as its concept and dimensions is conducive to in-depth study of antecedent variables and outcome variables of healthy organization, and also provides basic suggestions for practitioners to promote the construction of healthy organization. In this study, the healthy organization is defined from the perspective of contingency. On the levels of individual, organization and external environment, the healthy organization refers to a healthy state in which an organization can maintain its continuous growth and development while ensuring the healthy growth of its internal members, and has good interaction with the outside of the organization, covering employee health, organizational health and social responsibility.

**Acknowledgements**

This work was supported by a project grant from key cooperation projects of Beijing Police College (Grant No.2022KXJ15).

## LHMM040 THE LOGICAL DIMENSION AND PRACTICAL PATH OF INTEGRATING MENTAL HEALTH EDUCATION INTO AESTHETIC AND MORAL EDUCATION

Xing Yin^a^

^*a*^ *Department of Youth League Committee, Jiangsu Open University, Nanjing, Jiangsu 210000, China.*

**Background:** In the rapid developing world with increasingly fierce social competition, college students bearing high expectations form society and family suffer great pressure in study, life, emotion and employment, thus developing a variety of psychological problems such as depression, insomnia, anxiety and social phobia. Therefore, psychological problem of college students is worthy of great attention. It is necessary to investigate into the practical path of integrating mental health education into moral and aesthetic education, relieve college students’ psychological pressure, strengthen their psychological quality, and promote their harmonious development in body and mind.

**Subjects and Methods:** Mental health education of contemporary college students is taken as the research object to discuss the logical relationship between mental health education and moral, aesthetic education, seek the common points between the three, and promote the mental health education of college students via moral and aesthetic education.

**Results:** Mental health education, moral education and aesthetic education are all important parts in college education. The three assume the role of conveying truth, goodness and beauty. By integrating mental health education into moral and aesthetic education, it will not only create a new pattern of mental health education, but also consolidate the effect of mental health education.

**Conclusions:** To integrate mental health education into moral and aesthetic education, colleges and universities should first do a good job in top-level design, make overall management and let educators improve their cognition. In this way, it is possible to form the concept of collaborative education and build a grand pattern of collaborative education. Secondly, teachers should take the initiative to think, integrate the content of mental health education into the ideological and moral education curriculum, artistically implement mental health education, give vivid theoretical and practical classes to carry out multi-dimensional mental health education from multiple perspectives.

## LHMM041 EMOTION REGULATION SELF-EFFICACY AND DEPRESSIVE MOOD: THE MEDIATING ROLE OF EMOTION REGULATION STRATEGIES

Mengqi Xiao^a^, Xiaoting Liu^b^, Qingxin Huang^a^

^*a*^ *School of Educational Science and Technology, Guangdong Polytechnic Normal University, Guangzhou, Guangdong 510000, China,* ^*b*^ *School of Psychology, Key Laboratory of Behavioral and Mental Health, Northwest Normal University, Lanzhou, Gansu 730070, China.*

**Background:** With the outbreak of the Newcastle Pneumonia epidemic, the international community has now become increasingly concerned about the mental health of individuals, and the frequency with which youth, as the future of a nation, suffer from psychological problems has caused widespread public concern. Depressive disorders in adolescents and the adverse consequences they lead to have become the third leading cause of death. Among them, depression as expressed by depression is a common negative emotion in adolescents’ lives, and the emotion regulation strategies proposed based on the self-efficacy theory proposed by Bandura et al. can effectively alleviate depression, so this study will explore the causes of depression in adolescents by examining the emotion regulation strategies of adolescents and harm.

**Subjects and Methods:** 825 high school students (aged 14 to 18 years) completed three self-report measures: (1) the Scale of Regulatory Emotional Self-efficacy measured emotion regulation self-efficacy, (2) the Emotion Regulation Questionnaire measured emotion regulation strategies, and (3) the Beck Depression Inventory II assessed depressive mood.

**Results:** (1)Regulation self-efficacy has a negative impact on Beck Depression Inventory; (2)Regulation self-efficacy has a positive impact on Emotion Regulation Questionnaire; (3)Emotion Regulation Questionnaire has a negative impact on Beck Depression Inventory; (4)Emotion Regulation Questionnaire plays a mediating role in the relationship between Regulation self-efficacy and Beck Depression Inventory.

**Conclusions:** Taken together, our finding suggest that the frequency of use of emotion regulation strategies was associated with depressive symptoms in adolescents, and the more frequently adolescents used emotion regulation strategies, emotion regulation strategies significantly mediated the association between depressive mood and perceived self-efficacy in expressing positive affect, people with higher positive emotions levels tended to believe that expressing happiness could lead to positive effects; in the present study these individuals may considered not showing feelings as an inefficient way to manage mood, thus reducing the usage of expression suppression strategies. Emotion regulation strategies also mediated the relationship between depressive mood and perceived self-efficacy in managing negative affect. Therefore, in response to this result, the author believes that more attention should be paid to the psychological problems of adolescents, and through in-depth research on the inner mechanisms of adolescents’ emotion regulation strategies, the formation mechanism of positive emotions in adolescents should be clarified to improve their self-efficacy and alleviate their depression.

## LHMM042 AN IDEOLOGICAL TEACHING DESIGN OF MULTIMODAL CURRICULUM CLUSTER FROM PERSPECTIVES OF METAVERSE AND NEW ENGINEERING AND THE EFFECT ON MENTAL HEALTH

Rui Ding^a^, Rao Ma^a^, Jiahui Liao^a^, Hongbin Dong^b^

^*a*^ *College of Computer and Information Technology, Mudanjiang Normal University, Mudanjiang, Heilongjiang, China,* ^*b*^ *College of Computer Science and Technology, Harbin Engineering University, Harbin, Heilongjiang, China*

**Background:** The development of the educational metaverse is overwhelming. It gives birth to the second evolution of educational technology and greatly promotes the reform of educational modernization. Multimodal is an important direction of the technology re-development, which can strengthen the teaching expression of teachers, promote the transmission of teaching implications, and improve the teaching effect. It is the pioneering point of current teaching research. The new engineering teaching reform has stepped into the “superimposed” transformation stage, which condenses the high-quality teaching experience such as the curriculum cluster, the integration of industry and education, and the platform construction. It also clarifies the new teaching requirements of “technical education,” and derivatives the teaching difficulties such as ideological and political courses. Ideological and political education in professional courses is an important supplementary method to effectively alleviate or solve students’ mental health problems.

**Subjects and Methods:** At present, college students’ main mental health problems mainly arise from professional learning, emotional regulation and interpersonal relationship management. Ideological and political education in professional courses can effectively improve students’ mental health. Engineering courses have many problems, such as shallow ideological and political ideas, monotonous implementation forms, and isolated structures, which seriously affect the infiltration effect of ideological and political ideas. Integrating multimodal teaching and relying on the advantages of curriculum cluster can effectively solve the ideological and political teaching dilemma in new engineering courses and improve students’ mental health. Therefore, based on the dual perspectives of educational metauniverse and new engineering teaching, this paper explores the teaching optimization path of multimodal, curriculum cluster and mental health in the ideological and political teaching of software engineering courses. It alsocarries out the teaching design combined with the existing educational infrastructure and studies its impact on students’ mental health.

**Results:** Further, under the new engineering concept, the “causal diagram method and the design of test case” in “Software Quality assurance and testing” is taken as an example to explain the curriculum cluster teaching structure and the multimodal ideological and political teaching process from the educational metaverse perspective. Multimodal expression effectively improves the ideological and political effect. And the teaching design effectively improves students’ mental health.

**Conclusions:** The multimodal ideological and political teaching design of the science and engineering curriculum cluster based on the dual perspectives of the metaverse and new engineering can enhance the harmonious development of engineering professional knowledge goals and ideology, and promote the students’ mental healthy development. It provides a new idea for exploring the ideological and political teaching design of software engineering curriculum cluster from the perspective of the metaverse and new engineering.

**Acknowledgments**

This work was supported by the Key Foundation of Education Department of Heilongjiang (1355ZD005), Foundation of Mudanjiang Normal University under Grant 21-XJ21042 (Research and practice on the construction of software engineering professional course cluster for engineering education certification), and JPKC-2022011 (Quality Course construction for graduate course: Information Retrieval and Thesis writing).

## LHMM043 THE EFFECT OF WEBTOON ON PSYCHOLOGICAL HEALING UNDER MEDIA CONVERGENCE

Hu Shuhan^a^, Hu Shuling^b^

^*a*^ *School of Visual Communication, Luxun Academy of Fine Arts, Dalian 116650, China,* ^*b*^ *School of Architecture, Luxun Academy of Fine Arts, Shenyang 110004, China*

**Background:** Cartoons have an immeasurable impact on the healing of mental health, leading to the emergence of webtoon in the new media era, and forming a new narrative structure compared with traditional cartoons in the process of breaking through the media space.

**Subjects and Methods:** This paper focuses on the research of the expression form and psychological healing effect of webtoon in the environment of media convergence. Firstly, the relationship between cartoons and psychological healing was introduced, and then the emergence and popularity of the art form webtoon under the transmedia convergence were expounded. Next, the expression forms and creative methods of webtoon were analyzed and studied, and finally its far-reaching influence on psychological healing was summarized.

**Results:** With the development of the times, the expression of webtoons has changed further, and the creators have further optimized the design of storyboard, rhythm, blank and interaction, and paid more attention to the relationship between cartoon visual forms and psychological healing.

**Conclusions:** The artistic form of webtoon not only brings new vitality to the cause of cartoons, but also opens a new research and development direction on the role of psychological healing, with good development prospects.

**Acknowledgments**

The authors acknowledge the Humanities and Social Science – youth; 2020 science fund research project of Liaoning Provincial Department of Education “Research on comic visual design based on Internet mobile reading” (Grant: WQ 202012).

## LHMM044 RESEARCH ON THE APPLICATION OF ARTIFICIAL INTELLIGENCE TECHNOLOGY IN TELEMEDICINE SYSTEM

Binqiang Jing^a^

^*a*^ *Engineering College, Xi’an International University, Xi’an, Shaanxi 710077, China.*

**Background:** Since the beginning of the 21st century, the world has faced great changes unseen in a century. For example, anti-globalization, economic depression, global climate change, and the spread of global public health diseases, etc. However, the continuous progress of science and technology has brought dawn to people, and artificial intelligence technology has gradually entered the public’s field of vision. Especially in remote areas where medical resources are scarce, the application of artificial intelligence technology in telemedicine has important practical value and research significance.

**Subjects and Methods:** Based on the development of medical artificial intelligence and the specific application of artificial intelligence technology in real medical care, this paper uses the AI database update mechanism to update the database. Through the analysis of the user’s condition information, the best diagnosis plan is proposed. Solve the medical needs of users. To achieve the purpose of reducing the workload of medical staff while improving work efficiency, and effectively improve the level of medical services. This paper adopts the method of observation and literature analysis to study this topic.

**Results:** By reviewing the literature and field observation, it is found that the application of domestic artificial intelligence technology in telemedicine consultation and diagnosis services is still in the exploratory stage, and there are only simple conceptual frameworks and theoretical ideas, which need further research and development.

**Conclusions:** The application of high technology in the medical field is never a new discovery and application. However, it is unprecedented that modern medicine and science and technology are so closely integrated to present a new height. The application of high-end science and technology represented by artificial intelligence technology in the modern medical system, especially in telemedicine consultation and diagnosis services, is an inevitable trend, and has important theoretical and practical value.

**Acknowledgments**

This research was supported by a project grant from Special project of Education Department of Shaanxi Province in 2019 (Grant No. 19JK0726).

## LHMM045 TELEMEDICINE: GAPS IN MODERN MEDICINE FOR UNDERSERVED POPULATIONS

Cynthia Williams^a^, Di Shang^b^

^*a*^ *Department of Global Health Management & Informatics, University of Central Florida, Orlando, 32828 USA,* ^*b*^ *Department of Business Analytics, University of North Florida, Jacksonville 32828, USA*

**Objectives:** While technology has enhanced the accessibility of providers to patients, there is a portion of the population that does not benefit from the advances in modern medicine. In the United States, people who are on public health insurance, such as Medicaid, represent low-income individuals who often experience health inequities. In this project, we examine telehealth use among Medicaid recipients and compare this population to other public insurance programs (Medicare and Dual Eligible Medicaid/Medicare). We examine demographic, behavioral, and socioeconomic factors that contribute to inequities in telehealth access.

**Methods:** We examined 52,904 State of Florida (U.S.) Medicaid claims record of persons 21 years and older. The study period was March 2020 to December 2020. To examine the associations of socioeconomic factors with telehealth utilization, we retrieved data from American Community Survey Census and included persons whose income status are like those who qualify for Medicaid. Multiple logistic and linear regression, using R software, were used to examine study objectives.

**Results:** Medicaid patients are 8% less likely to use telehealth, while patients that are Dual Eligible are 7% more likely to use telehealth, as compared to Medicare only patients. Male patients are 22% less likely than females to use telehealth. Patients with diabetes are more likely to use telehealth than persons with chronic obstructive pulmonary disease (-5%) and heart failure (-14%). Patients in rural areas are 43% less likely to use telehealth as compared to patients in urban areas. Among vulnerable communities, the results of the multiple linear regression suggest that education and race were a statistically significant contributor to telehealth use, p-value <0.01.

**Conclusions:** Healthcare providers and professionals should be keenly aware of characteristics that contribute to low telehealth utilization among Medicaid recipients. We make recommendations to mitigate the gap between digital health and healthcare access among Medicaid beneficiaries.

## LHMM046 RESEARCH ON DECOUPLING INDEX OF COVID-19 AND CPI BASED ON G7 COUNTRIES

Ming-yung Chen^a^

^*a*^ *Minnan Normal University Business School, Fujian, China.*

**Background:** COVID-19, the Ukrainian war, the threat of food security and the resurgence of global poverty. Heat waves, droughts and other extreme weather events are not random shocks. They are not perfect storms in the traditional sense, that is, they are not a one-time concentrated outbreak of many bad things. The COVID-19 epidemic and its repeated mutated strains have made these concerns a reality, causing huge losses of life and property around the world. Then came the prospect of stagflation. Inflation intensified and economic growth stagnated for a period of time. Russia’s invasion of Ukraine opened a new chapter in international relations and had an important impact on the global economic order. The group of seven G7, composed of Canada, France, Germany, Italy, Japan, Britain and the United States, imposed a series of economic sanctions on the aggressors, and other countries soon joined the G7. In this study, covid-19 infection diagnosis data and CPI index of G7 countries from January 2021 to July 2022 were selected to analyze the influence of decoupling model.

**Subjects and Methods:** Decoupling means that the response relationship between two or more physical quantities with corresponding relationships does not exist. Since the 1990s, OECD has built an index model of absolute decoupling and relative decoupling based on DPSIR (driving force pressure state response feedback). The concept of decoupling has been widely concerned and recognized. Tapio introduced the elastic coefficient on this basis, and used the elastic decoupling index to describe the six decoupling states in more detail.

**Results:** The empirical result is DI < 1, that is, the development speed of COVID-19 is slower than the growth speed of CPI, and it is in a relatively decoupling stage. In a certain period of time, when the development speed of COVID-19 is less than the growth speed of CPI, it is considered that there is a relatively decoupling stage. The relatively decoupling stage indicates that the current global epidemic deterioration and speed are relatively slow, which is an ideal state.

**Conclusions:** The COVID-19 crisis has brought dramatic changes to people’s lives, but it has also prompted us to think about the possibility of change and innovation. The COVID-19 crisis has extensively affected all aspects of life and prompted people to try to change their consumption, work and other behavior patterns. In addition, the ways and locations of enterprise suppliers and services are different.

## LHMM047 MECHANISM FOR MONITORING AND COPING WITH NEGATIVE EMOTIONS OF HEALTH CARE WORKERS DURING THE NEW CROWN EPIDEMIC

Xidong Song^a^, Hong Wu^a^, Siqi Zhang^b^

^*a*^ *School of Economics and Management, Beijing University of Posts and Telecommunications, Beijing, China,* ^*b*^ *School of Business Administration, Jiangxi University of Finance and Economics, Nanchang, Jiangxi, China.*

**Background:** During the novel coronavirus pneumonia epidemic, health care workers experienced many negative emotions and mental health problems, such as insomnia, anxiety, and depression, due to stress in various areas, but they often did not acknowledge or were not willing to accept treatment for these problems. Health care workers are the main force in responding to and fighting the epidemic, and if their negative emotions are not properly guided and promptly resolved, they will adversely affect the epidemic prevention and control and the physical and mental health of individual health care workers. In this paper, by analyzing the psychological changes of health care workers when an epidemic occurs and the sources and manifestations of negative emotions, we develop corresponding coping mechanisms according to the different pressures faced by health care workers, aiming to alleviate and eliminate their negative emotions and improve their psychological ability to cope with public health emergencies, so that they can respond to emergencies better and more actively and quickly.

**Subjects and Methods:** Health care workers involved in the prevention and control of the epidemic during the New Crown epidemic were selected as the subjects of the study, and the literature research method and survey method were used to conduct the study.

**Results:** Many health care workers are under great stress, causing negative emotions such as insomnia, anxiety, and depression. There are three sources of negative emotions for health care workers: work pressure, family pressure and social pressure.

**Conclusions:** There are four main areas to address the negative emotions of health care workers: ensure that health care workers have enough rest time and rationalize their schedules; enhance the sense of support for health care workers in all aspects during self-isolation; provide appropriate rewards for health care workers who contribute during the outbreak; and official or other media should publish correct and authoritative information and policies about the outbreak.

## LHMM048 AN EMPIRICAL STUDY ON THE INFLUENCE OF SUMMARIZATION STRATEGY TEACHING ON THE READING PERFORMANCE OF JUNIOR THREE STUDENTS UNDER THE COVID-19 EPIDEMIC

Tang Xu^a^, Jiang Jie^b^

^*a*^ *College of Liberal Arts, Chongqing Normal University, Chongqing, China,* ^*b*^ *Panzhihua No.10 Junior Middle School, Panzhihua, Sichuan Province, China*

**Background:** Primary and secondary schools adopted online teaching after the COVID-19 outbreak, but its shortcomings also brought learning and psychological problems to junior three students, suggesting that teachers should teach students certain reading strategies in order to improve their reading ability and ease the pressure caused by the COVID-19 outbreak. This study was designed to verify the effectiveness of summarization strategy teaching in improving the reading performance of junior three students, provide empirical proof for the effectiveness of summarization strategy, and then provide the basis for the selection of Chinese teaching content in junior high schools.

**Subjects and Methods:** In this study, the quasi-experimental research method was employed, and the junior three students in a middle school in Panzhihua city were selected as the research objects, and the design of “unequal group pre-and post-test control group” was adopted. The pre-and post-test were conducted with the Test on Summarization Ability of Articles and Reading comprehension Test, and the data were statistically analyzed with the analysis of variance, paired sample T-test and independent sample T-test, to explore the effects of different experimental interventions on dependent variables.

**Results:** 1. Summarization strategy teaching helped to improve students’ summarization ability. 2. Summarization strategy teaching improved students’ reading performance, which was mainly reflected in the ability of “information extraction and retrieval” and “reflection and evaluation.” 3. Summarization strategy teaching improved student’s ability of distinguishing the authentity of information, which helped students to overcome their panic and anxiety caused by the information of the COVID-19.

**Conclusions:** Suggestions: 1. Summarization strategy should be included in the content of Chinese teaching in junior middle schools; 2. Attention should be paid to the cultivation of students’ metacognitive ability and promoting the development of summarization strategy teaching; 3. The summarization strategy should be used in conjunction with other strategies based on the content of the article; 4. Attention should be paid to teaching methods to improve the teaching effect of summarization strategy. 5. It taught students to distinguish the accuracy of information, guided students to face the COVID-19 with more positive attitudes.

## LHMM049 REALISTIC PROBLEMS AND STRATEGIES FOR THE INTEGRATION OF “SPORTS, MEDICAL AND ELDERLY CARE” IN THE COMMUNITY IN THE CONTEXT OF COVID-19

Cheng Ji^a^, Xin Li^b^, Kaili Zhang^c^

^*a*^ *School of Sports Science, Qufu Normal University, Qufu, Shandong 273165, China,* ^*b*^ *Shcool of Sciences, Xi’an Technological University, Xi’an, Shaanxi 710021, China,* ^*c*^ *School of Sports Science, Nantong University, Nantong, Jiangsu 226019, China.*

**Background:** Since its outbreak in 2019, the COVID-19 epidemic has brought unprecedented disaster to people all over the world. Throughout the history of human development, this is the first virus with a “short transmission cycle, large impact and high difficulty in treatment.” Therefore, in order to promote the ability of Chinese communities to defend against COVID-19 epidemic and enhance people’s physical health, the symbiotic relationship between “community sports service,” “community elderly care service” and “community primary medical service” is studied.

**Subjects and Methods:** Based on Bourdieu’s field theory, the inevitability and contingency of the integration and development of “sports, medical and elderly care” in the community are analyzed. What’s more, the basis for the realization of “sports, medical and elderly care” integration services in the community is researched, which are community “sports,” “medical” and “elderly care” are similar in approach and have the same goal and have industry penetration and pre-construction.

**Results:** The basis for the realization of the integration of “sports,” “medical” and “elderly care” services in the community is that there are similar service approaches, consistent service objectives, mutual industrial penetration and common pre-construction among the three.

The realistic shackles to the implementation of integration services include: community “sports, medical and elderly care” integration thinking forms to the old habits of the dregs; the break of interests and talent delivery chain leads to capital deficiency; the structural differences between the three are evident to form a field barrier.

**Conclusions:** Based on the mentioned problems, the three conclusions that are helpful to address the integration of “sports,” “medical” and “elderly-care” services in the community in the context of COVD-19 are as follows: (1) cultivate the habitual thinking of “sports,” “medical” and “elderly-care” integration and break the old health dregs; (2) create a complete interest chain and talent transfer chain to optimize capital flow efficiency; (3) paving the way for a community atmosphere of great health for the elderly and breaking the barriers of field integration.

## LHMM050 RESEARCH ON THE CENTENNIAL EVOLUTION OF PHYSICAL EDUCATION DISCIPLINE ABILITY IN PRIMARY SCHOOLS IN CHINA FROM THE PERSPECTIVE OF SPORTS MEDICINE

Kaili Zhang^a^, Cheng Ji^b^, Mengying Xue^c^

^*a*^ *School of Sports Science, Nantong University, Nantong, Jiangsu 226019, China,* ^*b*^ *School of Sports Science, Qufu Normal University, Qufu, Shandong 273165, China,* ^*c*^ *Graduate department, Jilin Sport University, Changchun, Jilin 130022, China.*

**Background:** In the past hundred years, sports medicine has been working together with the education concept of physical education health first to develop and promote health, with the content of sports intervention, sports and disease prevention, and sports rehabilitation. To help the development of sports medicine by cultivating students’ physical education ability is not only the manifestation of the physical education function of physical education, but also the manifestation of the return of physical education to the essence of life. The analysis of the reform characteristics of physical education ability in primary schools in the past hundred years can provide research evidence of historical texts and scientific basis for empirical analysis for the development of sports medicine in the new era.

**Subjects and Methods:** Using the method of literature and qualitative text from the basic ability, comprehensive ability, problem solving, cognitive ability, innovation ability of five dimensions to 25 copies of physical education curriculum standard for text analysis, illustrate the sports medicine horizon, four sports subject ability development period in one hundred the evolution logic of inner and outer evolution characteristics.

**Results:** By focusing on the nature, function of sports and sports and the relationship of modern medicine, to clarify the theoretical logic of body d depth fusion, and from the moment the needs of the students’ healthy growth, probing into the development of medical and health care, sports medicine horizon, sports discipline ability to change the information in one hundred: expounds basic ability is becoming more and more standardized; The content of comprehensive ability became richer and richer; The problem solving ability becomes more diversified and balanced; Cognitive ability from inside to outside to balance and diversity.

**Conclusions:** In the past hundred years, the discipline ability of physical education in primary schools in China has changed from independent separation to diversified integration, the co-development of students’ subjectivity and discipline practicality, the sustainable development concept and the value orientation of different historical stages contain four characteristics. In order to give full play to the effect of sports medicine in promoting students’ health, it is necessary to deepen the research on physical education ability from three aspects, namely, the different dimensions of evidence-based physical education ability, the concern for students’ emotional development, and the embodiment of the concept of sustainable development of education, so as to promote the construction of healthy China.

## LHMM051 RELATIONSHIP BETWEEN VACCINATION IN COVID-19 AND THE NUMBER OF PATIENTS AND SERIOUS ILLNESSES IN THE REPUBLIC OF KOREA FROM A FINANCIAL RISK TRANSMISSION PERSPECTIVE: AN ANALYSIS BASED ON VAR MODEL

Tao Wang^a^, Dan Wang^b^, Lina Yu^c^, Joohan Ryoo^a^, Kwi-sik Min^a^, Huiwen Guo^b^

^*a*^ *Graduate School of International Studies, Hanyang University, Seoul 04763, Republic of Korea,* ^*b*^ *School of Public Policy and Administration, Chongqing University, Chongqing, 400044, China,* ^*c*^ *School of Economics, Ocean University of China, Qingdao, 266100, China.TW, DW, LY, JR, KM, HG contributed equally to this work.*

**Background:** As global financial integration continues to deepen and gradually open up, the transmission of fluctuations between countries in the medical field of New Coronary Pneumonia is becoming more pronounced. Korea is an open economy, and the global movement of people and goods has led to the rapid spread of Neoplasms in Korea. The medical field of Neoplasms has become an important part of the Korean healthcare system. In the face of such outbreak control requirements, the NCC vaccine has become an important preventive measure for the Korean government.

**Subjects and Methods:** This paper presents an empirical study of the relationship between the NCC vaccine and the number of patients with NCC and the fluctuating impact of severe disease in the context of this study. In this paper, data on the number of newly crowned patients, the number of newly crowned patients with severe disease and the number of newly crowned vaccine recipients in Korea were selected to construct a vector autoregressive model through comprehensive analysis. Referring to the financial risk transmission model in this paper, the vector autoregressive (VAR) model can be used to describe the volatility transmission of different financial markets. With the help of VAR model, this paper studies the relationship between related variables. The model of the number of NCC patients with NCC vaccination and the model of the number of patients with NCC severe disease with NCC vaccination were used to empirically test the transmission problem related to NCC vaccination on epidemic prevention and control.

**Results:** Impulse response analysis of NCC vaccination on the number of NCC patients and the number of seriously ill patients showed that NCC vaccination can have a knock-on effect on the growth rate of both the number of NCC patients and the number of seriously ill patients. The variance decomposition of the number of new coronary patients and the number of seriously ill patients by vaccination showed that the new coronary vaccine had a significant effect on the growth rate of both the number of new coronary patients and the number of seriously ill patients.

**Conclusions:** Based on the above analysis, there is an equilibrium relationship between neo-crown vaccination and the fluctuating transmission of neo-crown and severe disease in the long term. The NCC vaccine program adopted in the Republic of Korea has been effective in controlling the spread of NCC virus and reducing the number of patients with severe disease.

**Acknowledgments**

Financial transmission, vaccine in COVID-19, COVID-19, VAR model

## LHMM052 MEDICAL SECURITY, ELDERLY CARE MODE PREFERENCES AND HEALTH PROMOTION AMONG THE ELDERLY FROM THE PERSPECTIVE OF COVID-19

Huiyong Song^a^, Min Deng^a^

^*a*^ *School of Health Economics and Management, Nanjing University of Chinese Medicine, Nanjing 210023, China*

**Background:** To analyze the elderly’s care mode preferences and its influencing factors quantitatively under the background of normalized prevention and control of COVID-19, and to explore the new development direction of elderly care service mode.

**Subjects and Methods:** This survey adopted the multi-stage stratified cluster random sampling method, mainly focusing on the family health inquiry survey. The survey objects were the actual members of the selected households. The researchers used tablet computers to carry out the face-to-face surveys at the objects’ homes. The statistical object was people aged 60 and above. Excluding invalid samples, the final sample number was 3,307. The factors of medical security, social support and mental health status were considered. Based on the data of the health service survey, chi square test and multi classification logistic regression model were used for empirical analysis.

**Results:** Medical security factors had a strong association with the elderly care mode preferences: the elderly who participated in serious illness insurance were more inclined to home-based elderly care mode; age, degree of education, employment status and household income also had a significant impact on the choice of elderly care mode: the young old aged 60-69 were more likely to choose home or community elderly care; The higher the educational level of the elderly, the higher the tendency to choose institutional care mode; Compared with retirees, employed people had a higher tendency to choose home-based care; The elderly with an annual household income of more than 100000 yuan preferred institutional care mode rather than home-based elderly care mode; The elderly with chronic diseases tend to choose institutional care mode than home-based elderly care mode.

**Conclusions:** Local governments should increase investment in medical security for the elderly and provide targeted medical assistance and economic support to special groups; In the face of the COVID-19, the traditional elderly care service model was difficult to fully meet the elderly with diversified needs. Under the COVID-19, the advantages of the integrated medical care service mode are highlighted; With the help of “Internet +,” promote the deep integration of intelligent elderly care and community and home-based elderly care, promote the combination of medical care and elderly care and the deep integration of various elderly care service modes.

**Acknowledgments**

This work was supported by a project grant from the Natural Science Foundation of China (Grant No.71804076).

## LHMM053 RESEARCH ON EMPOWERMENT TRAINING OF COMMUNITY HEALTH WORKERS IN PUBLIC HEALTH EMERGENCIES SUCH AS COVID-19

Lingshan Li^a,b^, Bo Su^a,b^, Zihan Yang^a^

^*a*^ *Department of Management, Hubei University of Chinese Traditional Medicine, Wuhan, Hubei 430070, China,* ^*b*^ *Research Center for the Development of Traditional Chinese Medicine, Key Research Institute of Humanities and Social Sciences of Hubei Province, Wuhan, Hubei 430070, China.*

**Background:** The occurrence and development of COVID-19 had brought huge negative impacts to society. Community health workers had made outstanding contributions in responding to the epidemic. The emergency response ability of community health workers plays an important role in the establishment and improvement of emergency management mechanism of public health emergencies.

**Subjects and Methods:** Through statistical analysis of the relevant data of community health workers in recent ten years, this paper makes an in-depth analysis of the responsibilities and role orientation of community health workers combined with the experience of coping with the COVID-19. The research data are mainly from the China Health Statistical Yearbook. Through the horizontal and vertical comparison of the data, this paper analyzes the role of community health workers in routine situations and in public health emergencies, in order to clarify the responsibilities of community health workers, and put forward countermeasures for improving the emergency management mechanism of community health service institutions.

**Results:** Through comparative analysis, it is found that community health workers have significant problems in dealing with public health emergencies, such as unreasonable academic structure, unreasonable professional title structure, unreasonable knowledge structure, the loss of emergency management talents. In order to solve these problems, community health workers need to receive corresponding empowerment training to improve their personal skills, so as to better respond to public health emergencies. This kind of empowerment training should be carried out gradually from the aspects of academic education, continuing education and skill improvement. Through cooperation with colleges and universities, the efficiency of training should be improved, and the ability of community health workers should be fundamentally improved.

**Conclusions:** Under public health emergencies, community health workers are the most important gatekeepers, and their work is directly related to the safety and health of every resident. Improving the working ability and knowledge reserve of community health workers in response to public health emergencies is the basis for improving the efficiency of emergency management. Only by carrying out empowerment training for community health workers can we be fully prepared and provide a solid guarantee for the safety of community residents.

## LHMM054 HEALTHCARE INDUSTRY DEVELOPMENT IN THE POST-EPIDEMIC ERA: A CASE STUDY FOR PANASONIC CHINA

Yourong Yao^a^, Leying Yao^a^, Mingjun Xu^b^, Feng Tang^b^

^*a*^ *Faculty of Social Sciences, University of Macau, Macau, China,* ^*b*^ *Faculty of Business Administration, University of Macau, Macau, China*

**Background:** With the outbreak of COVID-19, there has been a significant increase in the need for the healthcare industry and health products worldwide. The epidemic has triggered a great deal of attention to the health industry in China, and it has become especially important to better align the healthcare industry. Due to the changing demands of the healthcare industry in the post-epidemic period and the fluctuating market circumstances. In order to address the growth of the healthcare industry in the post-epidemic age, we analyzed the history of Panasonic’s healthcare industry in China, summed up its experience, and made recommendations for the future.

**Subjects and Methods:** This study analyses the current market situation of Panasonic’s healthcare industry in China and explores its market share and development in various industries in the health sector in recent years. It also explores the market potential of Panasonic’s healthcare industry in China, using the SCP model to further explore the market potential of Panasonic’s healthcare industry in China and provides a targeted outlook and recommendations for its future development strategy, in light of Panasonic’s current strategic layout in the Chinese market, the changing market trends in the post-epidemic era and the changes in the Chinese government and market policies.

**Results:** A comparison of the data reveals that Panasonic’s healthcare industry’s overall competitiveness in the post-epidemic era of the Chinese market has been considerably hampered by its premium positioning and limited audience reach. Panasonic has made strategic investments in three primary areas to solve this situation: product positioning, local development, and supporting personnel training. In order to further grow the healthcare sector, Panasonic is also collaborating with a number of insurance firms to deepen their collaboration by utilizing their respective capabilities in the healthcare industry, smart services, investment and finance companies, and insurance businesses. In the post-epidemic period, Panasonic may also utilize its air filtration systems to provide effective solutions to the air quality issues that are a widespread public concern.

**Conclusions:** With the advent of the post-epidemic era, the Chinese market is increasingly demanding clean technology and healthy ecology. Panasonic can build on its strong foundation in the health and elderly business to provide further “non-contact” solutions for the post-epidemic era. In addition, further positive signals from China to foreign businesses have provided Panasonic with succeeding sections options and possibilities. Panasonic can take advantage of the new chances presented by Sino-Japanese cooperation to achieve further growth.

**Acknowledgements**

This work was partially supported by Internal Funding (UM startup grant): Financial Statement Presentation and Information Usefulness.

## LHMM055 REFLECTION ON NEGATIVE METAPHORS FROM THE PERSPECTIVE OF PSYCHOANALYSIS IN COVID-19 REPORTS IN LIGHT OF UNCONSCIOUS THEORY

Shidong Zhang^a^, Shengping Xie^a^, Hong Song^a^, Shuang Peng^b^

^*a*^ *School of Foreign Languages, Northeast Normal University, Hangchun, China,* ^*b*^ *Literature Department, Northeast Normal University, Changchun, China.*

**Background:** Psychoanalysis is a major school of modern psychology. It is not only the treatment of neurosis, but also the psychological system of subconsciousness. Psychoanalysis is often used as a tool to study psychological reality to understand how the mind works and how the mind deals with the relationship between the external world and the internal world of the mind. The main task of psychoanalysis is to analyze the mechanism of the subconscious and make the subconscious conscious. The essence of metaphor is to understand another kind of things or experiences through one kind of things or experiences, and elaborate these unfamiliar fields through the combination of familiar concepts and unfamiliar fields. New Coronavirus pneumonia has triggered a subconscious defense response. The danger in reality may lead people to adopt pathological defense, symbolically repeating the trauma of the past.

**Subjects and Methods:** This study aims to encode negative metaphors such as “war, disease, devil, test and pain” and tries to demonstrate the reporter’s application of unconscious negative structural metaphors, apart from the consciously positive construction in the COVID-19 news texts in People’s Daily, Xinhua Daily Telegraph and CCTV News by the reporter, the readers will have negative concept after reading the news text unconsciously. In light of Freud’s theory of unconsciousness, this paper takes COVID-19 news texts in People’s Daily, Xinhua Daily Telegraph and CCTV News as corpus, with the aid of the data analysis software NVivo11, to analyze the frequency and types of unconscious use of metaphors, so as to reveal the unconscious adoption of negative metaphors.

**Results:** The data analysis of the metaphor construction reveals that two-tier structure exists: the surface structure that exposes negative metaphors (epidemic situation is war, disease, devil, crisis, pain, disaster, casualties) and the deep structure that sustain the negative metaphors (ideological structure - natural and man-made disasters). It is found that in official media reports, the reporters’ unconscious choice of negative metaphors has a negative impact on social reality, thus forming deep negative metaphors, which are ultimately expressed in the form of stream of consciousness. When the audiences or readers interpret the metaphors, the surface construction activates the deep construction. The latter, with its potential negative intention, exposes the hidden facts of the society, causing the public panic, worsening the negative emotions of the public towards the epidemic situation, and increasing some stream of consciousness of public distrust.

**Conclusions:** The analysis of the data suggests that the stream of consciousness influences the formation of communicative intention and the activation of deep structure when the reporter uses too much negative metaphors unconsciously, especially war metaphors. The danger in reality may lead people to adopt pathological defense, symbolically repeating the trauma of the past. In these cases, the study of negative metaphor based on psychoanalytic principles is helpful to prevent negative effects and formulate different intervention measures.

**Acknowledgments**

This research was supported by the National Social Science Foundation of China: Construction and Research of National Language Safety Big Data Platform (Grant/Award Number: 21&ZD287).

## LHMM056 INVESTORS’ TRADING STRATEGIES: FROM THE PERSPECTIVE OF INVESTMENT MENTAL HEALTH

Mingxu Peng^a,b^, Renshui Wu^a,b^, Song Chen^a,b^

^*a*^ *Business School, Taizhou University, Taizhou, Zhejiang 310000, China,* ^*b*^ *Zhejiang (Taizhou) Small-Micro Finance Institute, Taizhou, Zhejiang 310000, China*

**Background:** Since 2006, investment psychology has become an independent research direction in the financial field. This is related to household investment decision-making and household asset allocation. These achievements let us understand the behavior and constraints of investment psychological asset selection. Among them, mental health plays an important role in the process of asset selection affecting investment. This paper believes that mental health, as one of the factors of psychological stability, may be the key factor affecting investment decisions. Based on this idea, this paper constructs an empirical model for research. There are few studies on inventory in the research of limited price order market, especially in the analysis of trading strategy. Many studies on information are mainly based on the first-order original point distance rather than second-order distance. So, it is meaningful to construct a model which comprehensively combine the inventory and information in limit order book market. For the value of assets, investors have a different assessment from the perspective of pyschology, which leads to different valuations and different trading strategies. It is of great significance to analyze these trading strategies through models.

**Subjects and Methods:** After sorting out the relevant literature, this paper summarizes the possible impact mechanism of mental health on investment decision-making on the basis of existing research results. Among them, perhaps limited to the availability of data, “cognitive ability,” as an important part of the impact mechanism, is rarely studied in depth in the literature. Medical research shows that the deterioration of mental health will affect cognitive ability. In this context, the ability of decision-makers to receive, process and store information will be limited, which will affect investors’ optimization of their investment behavior and ultimately affect their enthusiasm to participate in the stock market. Therefore, this paper starts with the intermediary variable of “cognitive ability” to study the impact mechanism. This paper uses the data of the British Financial Investment Longitudinal Study from 2012 to 2022, takes the mental health risk as the core explanatory variable, and takes whether the family participates in the stock market, the depth of stock investment and the amount of stock investment as the three explanatory variables, respectively establishes Probit, Tobit and OLS models and carries out regression analysis. The trading strategy is studied by introducing the inventory and the information to construct a model in the limit order book market, where investors have different psychological judgement on asset. In general, investors are risk-averse from a psychological perspective. Therefore, a risk averse utility model with negative exponential utility is used to discuss the strategic interaction between investors, which determine the bid/offer price and the bid-offer spread in equilibrium, and make a comparative static analysis of the bid/ask price and the bid-ask spread in the limit order book market. Also, we study the impact of transaction probability, inventory of asset and precision of information on trading strategy.

**Results:** First, mental health is an important factor affecting investment decisions, and there is a significant positive relationship between them. When the mental health of the decision-maker deteriorates, the probability of participating in the stock market will be reduced, and the proportion of stock assets held by the investment units participating in the stock market will also be reduced, and the corresponding stock investment amount will also be reduced. Second, cognitive ability plays a part of intermediary role in the above influence process. Thirdly, after grouping the investment units with different income levels, we know that for low-income investment units, the positive impact of psychological health on their stock investment decisions is more obvious than that of high-income investment units. After grouping investors with different working conditions, we learned that investors are more likely to adopt a conservative stock investment strategy when facing the impact of deteriorating mental health. The above conclusions are still robust after replacing variables, delaying core explanatory variables by one period, and deleting unreliable samples. The execution probability, the inventory of risky asset held by investors, the risk-averse coefficient, the asset value and the precision of information, have an impact on the trading strategy, bid/offer price and the bid-offer spread. Among them, the inventory of risky asset will reduce the bid/ask price in the limit price order market, but its impact on spread is relatively complex, which involve in various factors.

**Conclusions:** From the perspectvie of psychology, a limit order book market model is builded by combining inventory model and information model. The inventory held by investors will reduce the bid/ask price; the inventory held by bidder increases the bid-ask spread while the inventory held by offeror reduces the bid-ask spread. The risk-averse attitude is negatively correlated with the the bid-ask spread. However, the impact of information precision on the bid/ask price and the bid-ask spread depends on the execution probability and the inventory. In addition, the price improvement is related to the execution probability, the inventory of risky asset, the risk-aversion coefficient, the private information and its precision. Tick size is also affected by the factors mentioned above.

**Acknowledgements**

This work was supported by a project grant from Jiangxi Social Science Planning Project (No.17YJ09) and Zhejiang Colleges and University Scientific Research Program No.Y202249734).

## LHMM057 ANALYSIS OF IDEOLOGICAL AND POLITICAL EDUCATION BASED ON STUDENTS’ PSYCHOLOGICAL HEALTH AND EMOTIONAL STABILITY

Yaozu Wang^a^, Shuguang Huang^b^, Qingyue Li^a^, Qian Liu^a^, Min Zhang^c^

^*a*^ *College of Education, Huaibei Normal University, China,* ^*b.*^ *Department of Education of Faculty of Education, East China Normal University, China,* ^*c*^ *Huaibei municipal Party School, China.*

**Background:** Emotional stability refers to the situation that people’s emotional state fluctuates with the change of external (or internal) conditions. Some people with relatively stable emotions are not easy to cause strong emotional reactions or slow emotional reactions for general situations. Emotional stability is one of the indicators that psychology judges a person’s personality traits. The most widely used Eysenck emotional stability test can be used to determine whether there is inferiority complex, depression, anxiety, obsessive-compulsive disorder, dependence, hypochondriasis and guilt. Under the continuous influence of the COVID-19 epidemic, contemporary college students often have various bad behaviors, such as lack of ideals and beliefs, confusion of ideas, declining moral consciousness, weak willpower, low mood and irritability. In the long run, it will have a very adverse impact on their learning and growth. Excellent traditional culture is the root and soul of a nation, the ideological cornerstone of a strong and prosperous country, and also an important rich resource and effective means to carry out education on ideals and beliefs, moral concepts, family and national feelings for college students.

**Subjects and Methods:** The EFT, EPQ and RSPM-CR scales were used to measure 156 science college students. The EFT test has three parts: the first part has nine questions for practice. The second and third parts each have 10 formal test questions, and the number of the simple figure required to be found is marked below each question. There are 9 simple graphics in total. The subjects were asked to quickly find a hidden simple figure from the complex figure. EFT reliability coefficient is 0.90. According to the requirements of the norm, the time limit for each part of the adult group is 4 minutes, a total of 12 minutes. The test score is scored according to the total number of simple figures correctly drawn in the second and third parts. 1 point for each question, with a full score of 20 points. In order to avoid the error of the scorer, the scorer is assumed by one person, and the scoring standard is unified. Only when the figure drawn by the subject is completely consistent with the specified simple figure in size and direction can it be considered correct. It is the basic requirement of curriculum ideology and politics to organically combine moral education with intellectual education, deeply explore and excavate the educating elements contained in professional courses, and promote the all-round development of students, as well as the important objectives of traditional cultural education and teaching. Based on the perspective of ideological history, this paper uses historical literature analysis, hermeneutics and comparative methods to study the core of Chinese traditional educational thought, excavate the educational elements of educational history course, and explore the ways and methods of classroom education.

**Results:** The graph reasoning scores of the subjects in the low and high emotional stability groups were tested. The t values of the scores and total reasoning scores of the two groups of subjects in groups A, B, C, D and E are: tA (50)=1.220, p>0.05; tB(50)= 1.983,p> 0.05; tC(50)= 0.421,p> 0.05; tD(50)= 2.543,p< 0.05; tE(50)= 1.751,p> 0.05; TTotal (50)=2.675, p<0.01. These results showed that the subjects with high emotional stability were higher than those with low emotional stability. Among them, the subjects with high emotional stability had a significant advantage in the score of group D and the total score of reasoning. Further investigation found that the difference in group D was mainly manifested in three questions: D8 (t (50)=2.132, p<0.05), D9 (t (50)=2.440, p<0.05) and D10 (t (50)=2.739, p<0.01). Although there was no significant difference between the two groups on the other questions (p>0.05), they also showed the advantage of subjects with high emotional stability, indicating that the degree of emotional stability had a certain impact on the effect of graphic reasoning. The history of Chinese education is rooted in humanistic thought, the ideal personality of sages, and the value orientation of emphasizing righteousness over profit, which is consistent with the main concept admired by the curriculum ideology and politics. Moreover, the history of Chinese education is the

main channel for students to systematically learn traditional culture, the foundation for teachers to establish morality and develop career, and

the spiritual resources for college students to be determined and persevere. Thus, it is not only the fundamental requirement of professional curriculum’s ideological and political education, but also the essential work of History of Chinese Education teaching.

**Conclusions:** The research shows that emotional stability also has a certain impact on the effect of ideological and political education for college students. The reasoning performance of those with high emotional stability is better than those with low emotional stability. Due to the implementation of time-limited test and the emphasis on the speed of reasoning process in this study, people with high emotional stability can quickly enter the stress state. This may lead to the formation of a psychological state suitable for completing this cognitive task. Positive and active emotional state is conducive to the smooth reasoning process. However, those with low emotional stability have low level of psychological arousal and slow response, and can not quickly use material information and create more psychological models. This will lead to slightly poor reasoning effect. Although the influence of introversion and extroversion on the effect of ideological and political education of college students shows a good trend of extroversion, the difference between introversion and extroversion is not significant. Generally speaking, extroverts may have broad thinking and high emotional stability, which will play a positive role in solving complex ideological and political education problems of college students. However, introverts tend to have a single mentality and low emotional stability, which may not be conducive to solving more complex ideological problems. Based on this, the article reveals the ideological value and educational process of “establishing morality and cultivating people” in the history of Chinese education from the following aspects: sharpening students’ firm will with the spirit of self-improvement, shaping perfect person with the ideal personality style, cultivating patriotism with the value of righteousness over profit concept, and casting excellent teachers with the atmosphere and tradition of honouring teachers and esteeming truth.

**Acknowledgements**

This work was supported by Key Project of Excellent Young Talents Support Program for Universities (gxyqZD2021013); Anhui Province Quality Project Curriculum Ideology and Politics Project (2020kcszyjxm214).

## LHMM058 CURRENT SITUATION OF LEISURE AGRICULTURE IN HANDAN CITY AND ITS IMPACT ON PUBLIC MENTAL HEALTH

Jiaqi Tian^a^, Rang Meng^a^, Yeda Zhang^a^, Lele Zhao ^a^, Haoran Zheng^a^

^*a*^ *School of Landscape and Ecological Engineering, Hebei University of Engineering, Handan city 056038, China.*

**Background:** Public mental health and mental health work is a systematic project, which needs to be strengthened from the whole process of public cognition, basic education, social psychology, patient treatment, community rehabilitation, service management, rescue and security, to adapt to the rapid growth of people’s mental health and mental health needs. The social cognitive level of mental health and mental health needs to be improved. With the rapid development of China’s economy and society, the pace of life has accelerated significantly, psychological stress factors are increasing, common mental disorders and psychological behavior problems such as anxiety and depression are increasing year by year, psychological stress events occur occasionally, and diseases of specific groups are widely concerned by all sectors of society. Most of the mental health and mental health problems can be alleviated through self-adjustment, family support and other ways. Some need psychosocial support, counseling and other help, and serious need professional treatment. In recent years, with the rapid development of social economy, people’s life quality is improving day by day, at the same time, the acceleration of urbanization process and survival the increase of cost makes people’s life pressure also increases, more and more. More city dwellers tend to get away from the hustle and bustle of the city during the weekend, come to the suburban countryside to open their own “diffuse life,” experience the Eastern Jin Dynasty poets Tao Yuanming, “picking chrysanthemums under the east hedge, leisurely see the South mountain” comfortable and comfortable. In this under the background, leisure tourism agriculture ushered in a good development opportunity, is become the new engine of rural economic development. As a new form of agricultural industry, leisure tourism agriculture is increasingly becoming the key to adjusting rural industrial structure and realizing rural revitalization an important way. Leisure tourism agriculture is based on agriculture, rural areas and farmers as the background, make full use of agricultural resources, agricultural landscape and rural environment, agriculture, forestry, pastoral and fishery production and rural cultural life as the support, leisure farm as the carrier to provide tourists with a new industrial form with the functions of viewing, entertainment, science popularization, experience and so on. Leisure tourism agriculture has risen since the late 1980s in Shenzhen Litchi Festival, its production and development into traditional agriculture new vitality Handan City is located in Lixia River water town, natural resources and cultural landscape is rich and diverse.

**Subjects and Methods:** This study analyzed the development status of leisure agriculture in Handan through SWOT analysis and concluded that Handan has advantages and opportunities to develop leisure agriculture.

**Results:** In addition to the dimensions of mental health (F=1.926, P>0.5) and psychological performance (F=1.748, P>0.5), leisure agriculture has significant differences in the presentation of psychological coordination (F=71.247, P<0.000), psychological activity (F=34.697, P<0.000), psychological depression (F=8.734, P<0.000), psychological regulation (F=33.467, P<0.000), overall psychology (F=3.977, P<0.000), psychological strength (F=31.847, P<0.000), and self-esteem (F=6.389, P<0.000). There was no significant difference in mental flexibility (F=3.553, P<0.05) and mental endurance (F=3.587, P<0.05). In terms of gender factors, there is no significant difference in the influence of different leisure agriculture modes on self-concept. There was no gender difference except for psychological coordination (F=31.781, P<0.000). However, there are problems of unsound public service system and low quality of rural human resources in the development process. It also faces challenges of homogeneous competition of leisure agriculture models in Hebei and diversified competition of leisure agriculture in the Beijing-Tianjin-Hebei region.

**Conclusions:** Leisure agriculture can improve the psychological self-concept, mood and mental health of the participants. This can promote the development of mental health in a positive direction and help promote the development of teenagers’ mental health quality. Different gender groups adopt different leisure agricultural activities, and their mental health benefits are also different. This shows the differences in style and gender and ultimately significantly promotes the generation of overall mental health benefits. We suggest scientific planning, enhancing competitiveness, focusing on strategic positioning and characteristic development, building a public service system, improving ecological and environmental awareness, strengthening professional training, and improving human resource quality.

## LHMM059 RESEARCH ON THE PROMOTION OF FOOTBALL MATCH TO TEENAGERS’ MENTAL HEALTH

Miao Shen^a^, Junyu Tang^a^, Zhijian Liang^b^, Pei Li^b^

^*a*^ *Guangdong University of Technology, Guangdong 510006, China,* ^*b*^ *South China Normal University, Guangdong 510631, China.*

**Background:** With the revitalization and advancement of China’s national economy, people’s living guidelines are incredibly progressed and the life pace gets to be quicker and speedier, which moreover brings numerous weights and mental burdens to individuals. Survey data in recent years show that people’s mental health is increasingly threatened, and adolescents’ are not exceptional. The tracking results of the physical and mental health of adolescents in China are not optimistic. As the main place for the development of youth sports activities, schools should shoulder the basic objectives of promoting students’ physical and mental health, improving students’ physical health, and cultivating students’ healthy personalities.

**Subjects and Methods:** There are 270 teenagers (151 boys and 119 girls), with an average age of 19.2 years, from four universities. The measuring instruments were adolescent health assessment questionnaire, adolescent physical self-concept questionnaire and mood questionnaire. This experiment adopts the natural intervention experiment of sports. Four groups of subjects were trained with football for 18 weeks. Different football training methods are 4 times a week, about 1 hour each time. About 1.5h on Saturday. Before and after the experiment, the subjects were tested for the same indicators. All data are processed on SPSS 11.0. The purpose of the study is to improve the overall mental health of adolescents through football activities, so that they can have a comprehensive development. The literature survey method and experimental method are employed, and the samples are randomly selected to investigate and analyze the promotion of football activities on student’s mental health.

**Results:** In addition to cognitive health (F=0.856, P>0.5), football training methods showed significant differences in physical function health (F=14.146, P<0.000), sports physical fitness (F=60.675, P<0.000), personality harmony and integrity (F=6.639, P<0.000), social contact integration (F=221.903, P<0.000) and other dimensions. There are differences in the dimension of emotional health (F=5.595, P<0.01). In general, participating in different football exercise activities can effectively improve the mental health of teenagers, and different exercise methods have different exercise effects. In terms of gender factors, the evaluation of mental health benefits of the same exercise mode and different gender groups is also different. There were significant gender differences in physical functional health (F=11.887, P<0.01) and cognitive health (F=4.252, P<0.5). However, there is no gender difference in sports physical health (F=0.303, P>0.5), personality harmony and integrity (F=0.061, P>0.5), emotional health (F=1.172, P>0.5), social contact integration (F=0.177, P>0.5) and other dimensions. The results show that the mental health of teenagers, who participate in football activities and competitions based on psychological training, is significantly promoted and improved, and their interpersonal quality and ability are also strengthened. Also, they show stronger independence and adventure spirit in daily learning and life, as well as stronger willpower and responsibility in the process of achieving their own goals, making themselves easier to accept the people and things around.

**Conclusions:** Different sports activities have different effects on mental health. Different ways of physical exercise can promote the development of various aspects of physical self-concept. Football activities have two-way adjustment function to mood. Football training can produce good mood balance benefits. Football activities can really improve people’s bad mood and improve the overall psychological health benefits of emotion. Therefore, football activities have a positive impact on adolescents’ mental health. The study provides a reference for the research on promoting people’s mental health in the future.

## LHMM060 THREE BODY INTEGRATION OF COLLEGE STUDENTS’ LIFE HEALTH EDUCATION FROM THE PERSPECTIVE OF MENTAL HEALTH AND EMOTIONAL STABILITY

Hengwei Xue^a^, Songchi Lin^b^

^*a*^ *WENZHOU VOCATIONAL COLLEGE OF SCIENCE & TECHNOLOGY, Wenzhou, Zhejiang 325000, China,* ^*b*^ *WENZHOU POLYTECHNIC, Wenzhou, Zhejiang 32500, China*

**Background:** The emotional stability of college students has an important impact on their mental health. The way of emotional situation induction can be analyzed by inducing the emotional response of the subjects through the step-up Sternberg dual task and examining the relationship between individual task performance and emotional stability and heart rate variability (HRV) in different difficulty tests. As college students are facing severe physical and mental challenges and their psychological characteristics are constantly changing under the background of the global epidemic of COVID-19, it is necessary to continuously carry out life health education to ensure their physical and mental harmony.

**Subjects and Methods:** A total of 807 students in grade 2019 from two schools of Wenzhou Vocational College of Science and Technology and Wenzhou Polytechnic were taken as the experimental subjects to investigate their emotional anxiety and psychological burden brought by the COVID-19 outbreak by means of questionnaire in March 2020 in order to enable students to actively learn about the concept of life, rational treatment of life in the confusion. In May 2020, three expected goals of students’ life health education were extracted, which were to guide students to form correct “three views” with students as the main body, construct a life theme system with teachers as the leading role, and promote physical and mental health harmony with life and health curriculum as the foundation. Through the establishment of life courses, the elements of education system will be perfected, and life education will start the cultivation of precise life consciousness elements, and reconstruct the life health education system of college students. During the life health education and teaching activities, the “three bodies” of teaching subjects (students), education conductors (teachers) and teaching mediators (courses) are integrated.

**Results:** There was no significant correlation between cognitive style and introversion and emotional stability (p>0.05), indicating that these three personality traits were highly independent. This result also supports the view that there is a qualitative difference between field-dependent and independent cognitive style and introversion and introversion. The two are not significantly related and can be considered as two different characteristics (dimensions) of personality. Cognitive style, emotional stability and the total score of graphic reasoning were significantly correlated, and both were positively correlated (r=0.437, p<0.001; r=0.152, p<0.05). Through the effective integration of teachers, students and courses, the expected goal of life health education in colleges and universities can be achieved by conciseness in many ways, optimization of media, multi-channel linkage and activation of teachers’ original motivation. In June 2022, statistics were conducted on a sample of 807 students from two schools. A follow-up study of the “three-body” integration of life health education conducted over two years showed that the “three-body” integration of life health education helped college students cultivate themselves in the “critical period,” and precision education promoted full-scale talent cultivation, with significant results.

**Conclusions:** Due to the implementation of time-limited test and the emphasis on the speed of reasoning process in this study, people with high emotional stability can quickly enter the stress state. This may lead to the formation of a psychological state suitable for completing this cognitive task. Positive and active emotional state is conducive to the smooth reasoning process. However, those with low emotional stability have low level of psychological arousal and slow response, and can not quickly use material information and create more psychological models. This will lead to slightly poor reasoning effect. Active exploration of scientific and effective “three-body” integration mode of life health education under the COVID-19 epidemic situation can help college students establish the correct three views, ensure the healthy growth of body and mind, and promote the all-round development of students.

**Acknowledgements:** This paper is a phased achievement of the 2019 Zhejiang Philosophy and Social Science Planning Project “Research on Cultivating the Sense of Attainment of Ideological and Political Theory Course for College Students in the New Era” (19GXSZ58YB) and the Zhejiang Provincial Ideological and Political Demonstration Course-Tax Planning Course.

## LHMM061 RESEARCH ON INFORMATION CONSTRUCTION OF NATIONAL TRADITIONAL SPORTS IN HIGHER VOCATIONAL COLLEGES FROM THE PERSPECTIVE OF PUBLIC HEALTH AND HEALTH

Hongyan Yao^a^, Gangqiang Dong^b^

^*a*^ *School of P.E and Sports, Hebi Polytechnic, Henan 458030, China,* ^*b*^ *College of Physical Education, Soochow University, Suzhou 215021, China*

**Background:** Psychological health, also known as mental health, is a psychological principle and method for protecting and enhancing people’s mental health. Mental health can not only prevent the occurrence of mental diseases, but also cultivate people’s character, cultivate people’s sentiment and promote people’s mental health. The content of mental health is very extensive. Different age groups have different psychological characteristics, and the content of mental health is also different. The COVID-19 outbreak in 2019 has posed a severe test to the health of people all over the world, as well as a major challenge and development opportunity for the education and teaching of the education system. Moreover, the breakthrough of scientific and technological problems and the wide application of internet plus have also set up a convenient platform for the education and teaching of the education system. National traditional sports, as the carrier of inheriting the fine traditional culture of the Chinese nation, pays more attention to the inheritance and development of patriotism, solidarity and cooperation while improving the physical fitness and sports skills of the whole people.

**Subjects and Methods:** In this study, college students from a school in a city were tested. The average age was 19.96 ± 0.85 years old. According to the scores of “cognitive reappraisal” and “expression inhibition” strategies, the subjects were divided into high and low groups, and the subjects in the experimental group who had significant differences in the scores of the two strategies were selected. According to the scores of “cognitive reappraisal” and “expression inhibition,” the subjects in the control group were 50% of the middle group. Finally, there were 100 effective subjects, including 50 in the positive emotion group and 50 in the negative emotion group. Ninety-eight online open courses related to sports in China’s MOOC (massive open online course) platform were taken as the research objects, which are divided into courses in sports colleges, normal colleges, junior colleges, higher vocational colleges and other undergraduate colleges according to the types of schools, so as to analyze and study the current situation and existing problems of the online open courses created by them.

**Results:** This study first revised the Emotional Questionnaire prepared by Dong Yan and Yu Guoliang (2007) according to the basic characteristics of the environment, and then used the revised scale to measure the subjects’ emotions. Among them, positive emotion (30 items in total) includes two dimensions of positive high arousal emotion and positive low arousal emotion, and negative emotion (42 items in total) includes two dimensions of negative high arousal emotion and negative low arousal emotion. The questionnaire adopts Likert’s 5-level continuous evaluation, “1” means “completely inconsistent,” and “5” means “completely consistent.” Statistics showed that the internal consistency reliability of the two subscales of positive emotion and negative emotion was 0.75 ~ 0.89, the four-factor structure validity is good, and the fitting index is: x2=245 21, x2/ df = 1. 96, CFI = 0. 94, TLI = 0.91, RMSEA = 0.050, SRMR = 0.067. The comparison of the overall construction of the course shows that the course covers a wide range of projects, and is mainly focused on special and characteristic projects, but rarely on projects that are easy to learn and more conducive to the health of the whole people, indicating an obvious imbalance. The comparison of course resources reveals that the construction of basic resources is relatively complete but with a single form of course resources construction, and there is a big difference between the expansion of resources construction, and there are obvious differences in the number and quality of course videos, the interaction between teachers and students and the assignment arrangement.

**Conclusions:** In the era of COVID-19 epidemic, based on the new economic development pattern of internet plus, solutions were proposed from the aspects of changing ideas, improving the level of information technology, and strengthening management, aiming to improve the national health level and the development level of national traditional sports information technology, and promote the great rejuvenation of national traditional culture.

## LHMM062 ANALYSIS OF THE IMPACT OF SOCIAL MEDIA USE ON COLLEGE STUDENTS’ MENTAL HEALTH

Chao Yang^a^

^*a*^ *State Key Laboratory of Media Convergence and Communication, Communication University of China, Beijing, China*

**Background:** College students’ mental health is that college students’ psychology has many characteristics of middle youth. As a special group, college students cannot be completely equal to the youth in society. Mental health is generally measured by the scale, and its standard is not fixed. Mental health standards change with the changes of times and cultural background. In this study, college students from a school in a city were tested. During the COVID-19 pandemic, students were often isolated in their homes, real-life social activities were reduced, and the restriction of physical activity space could have a negative impact on students’ psychology. The impact of social media on students’ mental health was magnified during the pandemic, as students relied on online social media for social interaction activities during the isolation period. This paper explored the impact of online social media use on the mental health of college students during the pandemic.

**Subjects and Methods:** The subjects of this study were 400 college students enrolled in five universities in Beijing. A multi-stage sampling of the study subjects was conducted through a questionnaire. The basic issues of online social media use (duration, platform, causes, effects), interpersonal interaction-related issues (frequency, initiative), and psychological feelings during use (social comparison perception, mimetic environment perception) were selected in the questionnaire, and the three dimensions of depression, interpersonal sensitivity, and anxiety in mental health were measured. The entire variable was constrained using satisfaction with social media use. The questionnaires were collected for data entry using Epidata software, and the resulting data were analyzed using SPSS 25.0.

**Results:** After data analysis, it was found that depression levels of school students are influenced by specific types of online social media use, specific social objects, upward social comparison, and satisfaction with use. Specific types of online social media use, specific social objects, and upward social comparison are among the reasons for their interpersonal sensitivity. Specific social objects, active posting, and upward social comparison all affect the anxiety level of college students in school and their life satisfaction is affected by upward social comparison and satisfaction with using online social media. The results of ANOVA of repeated measurements showed that after watching the negative emotion elicited fragments, the scores of negative emotion words (sadness, pain, fear, etc.) were significantly higher than those of the pretest, and the scores of positive emotion words (happiness, interest) were significantly lower. This shows that the negative emotion inducing fragments effectively induce the negative emotion of the subjects, and the selection of negative emotion inducing materials is reasonable. After watching the positive emotion inducing segment, the score of positive emotion words (happiness, interest) was significantly higher than the pre-test score, and the score of negative emotion words (surprise, fear) was significantly lower. There is no difference between the negative emotions (anger, disgust, sadness, contempt, embarrassment, pain) caused by the two clips. This shows that the positive emotion elicitation fragments can effectively induce the positive emotion of the subjects, and the selection of positive emotion experimental materials is reasonable. The level of optimism has significant inhibition and influence on the level of self depression, at 0.01 level.

**Conclusion:** Multiple aspects of online social media use were significantly associated with mental health, and the frequency of certain types of online social media use, upward social comparison and other dimensions will damage mental health to different degrees. The use of online social media has an important impact on the mental health of college students. This study can provide a basis for judging the mental health level of college students and subsequent treatment.

## LHMM063 ANALYSIS OF GENERATION RIGHTS AND CARBON EMISSION RIGHTS TRADE BETWEEN SIMILAR POWER SOURCES BASED ON PROFIT DISTRIBUTION MODEL FROM THE PSYCHOLOGICAL PERSPECTIVE OF EMOTIONAL BEHAVIOR CHANGE

Wanru Zhao^a^, Yuwei Duan^a^, Jinghua Wang^a^, Shan Gao^a^, Shuting Pan^a^, Juan Zhang^a^, Min Wu^b^

^*a*^ *State Grid Shanghai Municipal Electric Power Company, Shanghai, China,* ^*b*^ *East China Branch of State Grid Corporation of China, Shanghai, China*

**Background:** Situational correction is an effort and strategy to change emotions by changing and correcting a certain aspect and characteristic of the situation that induces emotions. For example, in the face of a quarrelling neighbor, there can be three solutions: leave, endure and stop. If you take the way of stop, go ahead and ask for noise reduction, it is the adjustment strategy of situational correction. The choice of situation and the correction of situation require individuals to change their environment. However, it is also possible to regulate emotions without changing the situation, because each situation has different aspects and different meanings, so that individuals can change the process of emotion generation by adjusting their attention and understanding. Climate change, caused by emissions of greenhouse gases such as carbon dioxide, is considered the biggest health threat of the 21st century, and carbon emissions themselves pollute the air, which in turn affects human health. On the basis of fair and reasonable initial distribution of power generation rights and carbon emissions, this paper introduces two independent markets, power generation rights trading and carbon emissions trading, into the free trading market at the same time.

**Subjects and Methods:** In order to study the impact of carbon emissions trading on people’s emotional behavior, especially the factors of investor anxiety. The QSC scale revised by the Chinese Academy of Sciences was used for reliability and validity analysis. The total correlation coefficient of risk investment decision, emotional anxiety, work stress and job satisfaction revision items is greater than 0.5. The scale in this paper has high reliability. In order to analyze the effectiveness, this paper uses the scale used in each structure of this paper. This paper takes emotional labor as an independent variable and work stress as a dependent variable to explore the impact of emotional labor on work stress. F value is 10.229, R2 is 0.05, P value is 0.002<0.001, β The value is negative, F value is 28.667, R2 is 0.129, P value is 0.000<0.001, and β A positive value indicates that the level of psychological status has a positive and significant impact on drug investment. This paper aims to establish a new combination trading model involving similar power sources with the goal of maximizing the revenue of power generation enterprises. From the perspective of economic benefits, this model converts the interest difference generated by units with different installed capacity and the carbon emission reduction generated by carbon emission trading into the revenue of power generation enterprises, so as to quantify the economic and environmental benefits. Through the example analysis, the rationality of the constructed model and method is verified. Finally, according to the contribution degree of each participant, the profit is reasonably distributed by Shapely value method.

**Results:** There is multicollinearity in the model using all seven factors. With the help of principal component analysis and factor analysis, the information of all factors can be reflected through the information of several indicators. This will be discussed in depth in the next document. In particular, as a global public health emergency, the severity of the novel coronavirus has caused great panic worldwide, shaken the hearts of investors and triggered fluctuations in the stock market. The pandemic has had varying degrees of impact on the return on drug stocks in the most affected countries in the world. However, with the control of Internet public opinion and the stabilization of investor sentiment, the impact is significant in the short term. This will help China improve the stock market emergency mechanism and formulate phased and differentiated economic incentive policies. This provides a theoretical basis for investors to respond reasonably to the the Belt and Road investment in emergencies. In the combination trading mode, the smaller unit transfers the installed capacity to reduce carbon emissions and buys the carbon emission right in the market, while the larger unit buys the installed capacity to play the advantage of energy conservation and emission reduction. After the transaction, the total profits of enterprises can be increased, and the economic benefits are significant. Can reduce carbon emissions, reduce the effect of obvious, significant environmental benefits.

**Conclusions:** After trading in the portfolio trading mode, the economic and environmental benefits are significant. Carbon price is an external factor that affects portfolio trading. Increasing the number of enterprises participating in the transaction and expanding the scope of the transaction can optimize the allocation of resources. Profit distribution mode can realize the adjustment of power generation structure through market means.

## LHMM064 THE POWER GENERATION EFFICIENCY EVALUATION CONSIDERING GREEN CONSTRAINT UNDER MARKET MECHANISM OF CHINA BASED ON SUPER-EFFICIENCY DEA

Naitian Gao^a^, Weijie Mao^a^, Jiaoqian Gao^b^

^*a*^ *Yancheng Power Supply Company, State Grid Jiangsu Electric Power Company, Yancheng, Jiangsu, China,* ^*b*^ *Qingpu Power Supply Company, State Grid Shanghai Electric Power Company, Qingpu, Shanghai, China.*

**Background:** The composition of emotion includes three levels. Many emotion researchers mostly study and define emotion from three aspects: subjective experience on the cognitive level, physiological arousal on the physiological level, and external behavior on the expression level. When emotions are generated, these three levels work together to form a complete emotional experience process. The subjective experience of emotion is a kind of self-awareness of people, that is, a feeling state of the brain. People have many subjective feelings, such as happiness, anger, sadness, love, fear and hate. People’s attitudes towards different things will have different feelings. China is the second largest power producer in the world, but its proportion of highly polluting power generation is relatively high, affecting the environment and public health. For this reason, China has adopted an incremental reform model to adjust the factors in the power field, but little has been achieved.

**Subjects and Methods:** This scale is used to measure the reliability of the subject’s assessment of the behavior, commitment or (oral and written) statement of others. There are 25 projects in total, involving interpersonal trust in various situations and different social roles. Most projects are related to the credibility of social roles, but some projects are related to the degree of optimism about the future society. The five-point symmetrical scoring method is adopted, with 1 point for complete agreement and 5 points for complete disagreement. Therefore, the total score of the scale ranges from 25 points (the lowest degree of trust) to 125 points (the highest degree of trust), with a median of 75 points. The test time is about 10-15 minutes. The author used this scale to test 4605 groups in the 1960s. From the perspective of reliability and validity, the reliability of this scale is 0.76. Among them, 0.77 for men and 0.750 for women, the retest reliability with an average interval of 7 months was 0.56 (P (0.01, n=24), while the retest reliability with an interval of 3 months was 0.68 (n=42). Research on structural validity suggests that the score of this scale reflects the differences in family background and social class, but unlike the philosophical scale of human nature, there is no gender difference in the score of this scale. Since items that are significantly related to other scales are avoided when selecting items, the differentiated validity of this scale is guaranteed. This paper uses the super efficiency DEA model to scientifically evaluate the panel data power total factor productivity of 29 provinces in China from 2012 to 2021. On this basis, this paper analyzed the dynamic evolution characteristics of power total factor productivity of China by the Malmqusit index decomposition.

**Results:** FNE score is moderately correlated with other scales related to social anxiety, such as social avoidance (SAD) scale (r=0.51) and interaction anxiety scale (r=0.32). Compared with those with low scores, those with high FNE scores are more anxious when being evaluated, more likely to agree with the “irrational belief” that it is important to be liked, and more concerned about making a good impression. Similarly, people with high FNE scores experience higher anxiety and worry more about possible negative evaluation when being evaluated. The original FNE scale was low correlated with the Marlowe-Crowne Social Expectation Scale (r=-0.25). The power total factor productivity of China was relatively low, and it had improved slightly after 2010, and there were significant provincial differences; The value of power total factor productivity considering carbon emissions was relatively high, showing a certain “Potter effect”; China’s power total factor productivity growth was mainly due to the improvement of technological progress.

**Conclusions:** The growth of power total factor productivity of China electricity mainly comes from technological progress, and the overall power total factor productivity still needs to be improved.

## LHMM065 INSPIRATION OF SSI SAFETY MODEL TO SAFETY WORK OF LARGE-SCALE ACTIVITIES FROM THE PERSPECTIVE OF MENTAL HEALTH

Danyang Li^a^, Tuo Shi^a^, Wenjing Song^b^

^*a*^ *Department of Public Security Administration, Beijing Police College, Beijing, 102202, China,* ^*b*^ *Beijing Municipal Public Security Bureau, Beijing, 100000, China*

**Background:** Mental health refers to that all aspects of psychology and activities are in a good or normal state. The ideal state of mental health is to maintain a state of good character, normal intelligence, correct cognition, proper emotion, reasonable will, positive attitude, proper behavior and good adaptation. Mental health is affected by both heredity and environment, especially the upbringing style of the original family in childhood, which has a great impact on the development of mental health. Mental health highlights the ability to maintain good communication or cooperation with other people in social, production and life, and to deal with various situations in life. The normalization of epidemic prevention and control in COVID-19 marks China’s entry into the “post-pandemic era,” but its volatility, uncertainty, complexity and fuzziness bring new challenges to the security work of mega events. In view of the epidemic situation that may break out at any time, all aspects of the security work of mega events need to be continuously improved and upgraded to enhance the social prevention and control ability under the epidemic situation and the ability to cope with various risks.

**Subjects and Methods:** This paper also uses the multi-dimensional health status locus of control scale. From the perspective of the source of psychological control, MHLC assessed the view of health in three areas: the internality of Levenson, the influence of influential others and the role of opportunity. MHLC has two parallel versions, each of which is divided into three parts, and each part has six entries, all of which are self-evaluation scales. The scores of each subscale range from 6 to 36. MHLC samples include volunteers and performers participating in large-scale activities. In this paper, the SSI (Security, Safety & Integrity) model proposed by the International Centre for Sport Security was selected, and the security tasks under the COVID-19 were analyzed in combination with the 2022 Beijing Winter Olympics.

**Results:** Normal model data are in IHLC M-26 (SD=5), CHLC M=15 (SD-6), PHLC M=20 (SD=5.5). The internal consistency study shows that IHLC Cronbach a=0.61-0.80, CHLC) 0.55-0.83, PHLC 0.56-0.75, and the correlation of each component between the two parallel versions is IHLC0.48-0.77, CHLC0.38-0.65, and PHLC0.46-0.53. After an interval of 4 to 6 months, the three subscales of retest reliability correlation values were IHLCO.66 CHLC0.73 and PHLC0.71 respectively. Combining the security work with the life cycle of project management, the International Centre for Sport Security connected the management functions in series in chronological order, and defined security as eight steps, namely, due diligence, strategy, planning, implementation, testing, execution, close out and legacy, which provides a brand-new theoretical perspective for the security work of mega events under the epidemic situation.

**Conclusions:** This study found that the psychological quality of participants in large-scale activities was significantly positively correlated with cognitive reappraisal, and significantly negatively correlated with expression inhibition. Researchers believe that the development of metacognition is the basis for the development of emotion regulation strategies of participants in large-scale activities, while psychological quality consists of cognitive quality, personality quality and adaptability, and the core of cognitive quality is metacognition. In addition, the adaptability of psychological quality also includes emotional adaptation, which is closely related to emotional adjustment strategies. Therefore, psychological quality is closely related to emotion regulation strategies. This study also verified that cognitive reappraisal was significantly negatively correlated with negative indicators of mental health, while expression inhibition was significantly positively correlated with negative indicators of mental health. In the “post-pandemic” era, SSI model inspires us to improve the security of large-scale events from three time nodes: pre-event top-level design, in-event operation management and post-event legacy feedback. Specifically, in the event preparation stage, attention should be paid to the comprehensiveness of the due diligence findings and strategic guidance, and special plans should be refined continuously; During the operation of the event, the strategic planning should be scientifically implemented, and the feasibility, applicability and effectiveness of the diagnostic planning should be constantly tested; After the event closes out, the event should be summarized to form a security legacy.

**Acknowledgements**

This work was supported by the Key Project of School-Bureau Cooperation in Beijing Police College “Research on the Integrated Operational Command System of Security for Mega Events in the Capital —— A Case Study of 2022 Winter Olympics” (2022KXJ17) and the Beijing Social Science Fund Project “Research on the Optimization of Capital Public Security Police Command System under the Background of Integration of Intelligence, Command, Service and Public Opinion” (22JCC099).

## LHMM066 THE PRACTICAL PREDICAMENT AND COUNTERMEASURES OF MENTAL HEALTH EDUCATION IN JUNIOR MIDDLE SCHOOLS IN CHINA

Yu Wang^a^, Kai Jiang^b^

^*a*^ *Northeast Normal University School of Physical Education, Changchun, Jilin 130024, China,* ^*b*^ *No. 1 Secondary Specialized School of Changchun, Changchun, Jilin 130033, China.*

**Background:** Individuals can adapt to the developing environment and have perfect personality characteristics; And their cognition, emotional reaction and will behavior are in a positive state and can maintain normal control ability. In life practice, the basic characteristics of mental health have been achieved by correctly understanding oneself, self-consciously controlling oneself, correctly treating external influences, and thus maintaining psychological balance and coordination. The basic meaning of mental health is that all aspects of psychology and the process of activities are in a good or normal state. The ideal state of mental health is to maintain a state of perfect personality, normal intelligence, correct cognition, proper emotion, reasonable will, positive attitude, proper behavior and good adaptation. Corresponding to mental health are mental sub-health and mental illness. Mental health has different meanings from different perspectives, and its measurement standards are also different. School health education is the foundation of health education for the whole people, and the junior high school stage is the most critical period for students to acquire health knowledge and develop healthy behaviors. Therefore, the current situation of health education in junior high school has become one of the hot topics. Health education through the classroom teaching and health education activities, make children master the common disease prevention and health care knowledge, enhance students’ self-health care consciousness, develop a scientific, civilized and healthy lifestyle and behavior habits, so as to prevent diseases and improve health, improve the health level of students’ individual and group. Subjects and Methods: In this study, junior high school students from a school in a city in the north were tested. They were half male and half female, aged between 13 and 16 years (the average age was 14.96 ± 0.85 years). According to the scores of “cognitive reappraisal” and “expression inhibition” strategies, the subjects were divided into high and low groups, and the subjects in the experimental group who had significant differences in the scores of the two strategies were selected. According to the scores of “cognitive reappraisal” and “expression inhibition,” the subjects in the control group were 50% of the middle group. Finally, there were 100 effective subjects, including 50 in the positive emotion group and 50 in the negative emotion group. This paper adopts literature method, policy analysis and so on to understand that at present, our country`s policy document pays high attention to junior high school health education, but the idea of “health first” in the teaching process is more random, and the evaluation standard is uncertain. Health education has no independent subject status; although the teaching of health education infiltrates in many disciplines, the overall class time is less, the textbooks are not unified, and low-level repetition phenomenon of the health education teaching content of each subject is more common. There is the phenomenon of emphasis on cognition and neglect of practice. Non-systematization of content, non-specialization of teachers and the unification of teaching materials make the junior high school health education into a difficult situation.

**Results:** The results of ANOVA of repeated measurements showed that after watching the negative emotion elicited fragments, the scores of negative emotion words (sadness, pain, fear, etc.) were significantly higher than those of the pretest, and the scores of positive emotion words (happiness, interest) were significantly lower. This shows that the negative emotion inducing fragments effectively induce the negative emotion of the subjects, and the selection of negative emotion inducing materials is reasonable. After watching the positive emotion inducing clip, the score of positive emotion words (happiness, interest) was significantly higher than the pre-test score, and the score of negative emotion words (surprise, fear) was significantly lower, and there was no difference between the negative emotions (anger, disgust, sadness, contempt, embarrassment, pain) caused by the two videos. This shows that the positive emotion elicitation fragments can effectively induce the positive emotion of the subjects, and the selection of positive emotion experimental materials is reasonable. The level of optimism has significant inhibition and influence on the level of self depression, at 0.01 level. To solve the practical difficulties in the development of health education in junior high schools in China, it is necessary to change from subject infiltration to subject independence and perfect the content system of health education. It is also necessary to improve the content system of health education, professional health education teachers and various textbook writing.

**Conclusions:** To promote the work of school health education, to master the process of school health education activities, adjust and determine the

goals of school health education, continuously improve and perfect the school health education, and gradually make the school health education work into the scientific and standardization of the track, in line with the principle of scientific, comprehensive, comparable, feasibility, formulate scientific and reasonable evaluation scheme.

## LHMM067 MENTAL HEALTH AND STABILITY OF GREY PREDICTION MODEL BASED ON MATLAB ANALYSIS OF THE DEVELOPMENT TREND OF CHINESE WOMEN’S THROWING EVENTS AND PREDICTION OF OLYMPIC RESULTS

Yongguo Zhu^a^, Mengfei Liao^b^, Houlin Li^c^, Xiao Ma^b^

^*a*^ *Institute of physical Ice and Snow, Capital University of Physical Education and Sports, Beijing 100191, China,* ^*b*^ *Graduate school, Capital University of Physical Education and Sports, Beijing 100191, China,* ^*c*^ *Institute of physical education and training, Capital University of Physical Education and Sports, Beijing 100191, China.*

**Background:** Mental stability is a state. It means that the environment or mood will not change easily in a certain amount of time. People’s psychological stability has a great impact on athletes’ performance in competitive sports. The CORONAVIRUS pandemic has caused the most significant disruption to the worldwide sporting calendar. Especially, it caused the postpone of 2020 Tokyo Olympic Games. Women’s throwing events are the dominant track and field events in China. In order to deeply understand the development trend of the world women’s throwing events under the Coronavirus pandemic and provide theoretical data for Chinese women athletes to prepare for 33rd Olympic Games.

**Subjects and Methods:** The anxiety scale was developed in the process of developing the self-consciousness scale, which contains 6 items. A score of 0 indicates a low degree of focus deficit, while a score of 24 indicates a high degree of focus deficit. Cheier and Carver (1985) noted that subjects without college education often have difficulties in understanding some items in the original scale. So the scale was revised to make it more understandable. The contents of the items in the social anxiety scale in the new table have not changed, but the text has some minor modifications. At the same time, the score is also changed to a four-level system (0: not like me at all, 3: very like me). The score range of the scale is from. (low anxiety level) to 18 (high anxiety level). The correlation coefficient between the revised scale and the original scale is 0.86. This article used the method of literature research, mathematical statistics, and logical analysis analyzing the 8 best from the 27th to 32nd Olympic Games as well as in the 2019 Doha World Championships and used grey prediction model to predict the top 3 results of women’s throwing events in the 33rd Olympic Games.

**Results:** The average value of 179 male athletes evaluated by the original scale (5-grade scoring system) is 12.5 (SD=4.1). When the revised scale is adopted (4-level scoring system). The average of 213 male athletes was 8.8 (SD=4.3), while the average of 85 female athletes was 8.6 (SD=4 7) Another sample is 396 middle-aged female athletes, whose scores are slightly lower. The average value is 7.3 (SD=3.9). Cronbach of the original scale α Cronbach with coefficient of 0.700 revised scale α the coefficient is 0.79. The reliability of the two-week retest of the original scale was 0.730, and the correlation coefficient of the four-week retest of the revised version was 0.77. The results show that overall development of javelin is not stable and shows a decreased trend, while it is an increased trend of shot put, discus, and hammer. The trend of shot put and discus is stable while it is not of hammer. The overall results in the 2019 Doha World Championships declined affected by the pandemic. Comparing with the performance of foreign athletes, the results of Chinese athletes fluctuated little.

**Conclusions:** The predicted results of women’s javelin throw, shot put, discus throw and hammer throw in the 33rd Olympic Games were 64.4454m, 20.9622m, 70.3636m and 81.9753m respectively. The predicted average scores of the top three were 64.4592m, 20.4406m, 67.8754m and 79.0209m respectively.

**Acknowledgments**

This work was supported by a project grant from the Great Wall Scholar Training Program for the Construction of High-level Teachers in Beijing Municipal Universities (Grant No.CIT&TCD20190335).

## LHMM068 THE SUSTAINABLE DEVELOPMENT OF CROSS-CULTURAL TEACHING IN PRIMARY SCHOOLS BASED ON THE ANALYSIS OF STUDENTS’ MENTAL HEALTH

Lisong Wang^a^, Yutian Fu^b^, Yanyan Bao^c^

^*a*^ *School of Foreign Languages, Tianjin University, Tianjin300350, China,* ^*b*^ *School of Foreign Languages, Tianjin Ren’ai College, Tianjin 301636, China,* ^*c*^ *School of Science, Tianjin University, Tianjin300350, China.*

**Background:** With the development of society and the progress of the times, more and more people pay attention to students’ mental health. Mental health education is a very important part of quality education in China, because mental health can enable students to face life with a positive attitude and play a positive role in their study and life. Mental health education will also have a certain impact on students’ behavior norms, mainly because mental health can promote students’ good behavior and further play a positive role in the healthy growth of students. Given the booming public awareness that education functions to foster the comprehensive development of humans to better serve social development, today’s school system and families begin to attach extra emphasis to converting traditional teaching into an updated mechanism where cross-cultural competence should weigh more than ever. cross-cultural education plays an essential role in the sustainable development of students and society. However, this new type of education has not received much attention in China, where the traditional grade-oriented education has impeded students from acquiring critical and creative thinking and reinforcing self-discipline in learning. When education aims to remold human cognition, its role in probing the relationship between learning and behaviors should go explicit to help improve students’ cross-cultural performance as a prerequisite to allowing better compatibility to the society where cross-cultural competence is desired more today than ever.

**Subjects and Methods:** This study also uses the Trait Anxiety Scale to measure students’ mental health. This group of scales reflects three different components of psychological control, each of which can be regarded as independent, with the purpose of describing some of the subjects’ views on causality. Internal control measures the extent to which people believe they can control their own lives. The influential others (P) scale involves whether subjects believe that others can control events in their lives. The opportunity scale measures a person’s degree of confidence that opportunities can affect his/her life experience and outcome. The IPC subscale includes items in the RotterI-E scale, and some items are specially prepared to evaluate the components of psychological control. Each subscale contains 8 items, and the whole scale has 24 items in total, which are evaluated by the 7-point system. All items are stated in the first person, and the score ranges from - 3 (very disagree) to+3 (very agree). When calculating the total score, 24 points are added to offset the negative score, so the score range of each subscale is 0-48. This paper is a dedicative research attempt to explore the significance of cross-cultural teaching in comparison to its non-intercultural, traditional counterpart. To highlight the difference between the two and necessity to instill in students’ cross-cultural consciousness, the research was based on feedback from a comparative study of two groups of participants using questionnaires to reflect various experiences of primary students in terms of their behavioral performances implying cross-cultural traits. The research cut into the topic from perspectives of students’ thinking and cognitive abilities, competence in innovation, and self-motivation toward learning under the spotlight of theories like the Herrmann Brain Dominance Instrument (HBDI), of statistical means for visualizing objective observation and of behavioral patterns to interpret self-discipline.

**Results:** The concise scale ranges from 12 to 60. The opposite of high FNE is that it does not take advantage of others’ evaluation, and it is not necessarily expected or needs to be positive. The average score of 205 college students in the original table is 15.5 (SD=8.6), and the score is rectangular distribution. The average score of the other sample composed of 128 subjects is 13.6 (SD=7.6) a The average score of the sample (n=150) used to prepare the 12-item concise scale is 35.7 (SD=8.1). The results of the analysis revealed that students with less cross-cultural education have relatively poorer performance in the scope of sustainable development than those who have been exposed to cross-cultural atmospheres, demonstrating greater improvement in students’ psychological maturity, critical thinking, potential in less deviant behaviors and cultivation of their long-term characteristics in a positive way.

**Conclusions:** Coming with this investigative finding is its applicational suggestion to promote cross-cultural teaching in primary education to allow younger students to reap benefits from pedagogical updates and even education reform in which new measures to highlight cross-cultural awareness should be incorporated.

**Acknowledgements**

This work is supported in part by the Tianjin Philosophy and Social Sciences Research Program Funding Project under Grant TJWW18-004.

## LHMM069 RESEARCH ON THE CONSTRUCTION SCHEME OF CIVIL ENGINEERING TEACHERS IN CHARACTERISTIC APPLICATION-ORIENTED UNIVERSITIES AND TEACHERS’ MENTAL HEALTH BASED ON PROFESSIONAL CERTIFICATION

Rui Pan^a^, Jixiang Liao^a^, Shaonan Han^a^, Dayong Wang^a^, Jingbo Yu^b^, Peifeng Cheng^c^

^*a*^ *Harbin University, Heilongjiang, Harbin, 150086, China,* ^*b*^ *Heilongjiang University of Engineering, Heilongjiang, Harbin, 150050, China,* ^*c*^ *Northeast Forestry University, Heilongjiang, Harbin, 150040, China.*

**Background:** There are three main factors in cognitive reconstruction: distraction refocusing, cognitive reappraisal and constructive refocusing. Distraction and refocusing refers to individuals thinking about other things, preferably positive things, instead of keeping in touch with emotional stimuli or events. Cognitive reappraisal refers to changing emotions by changing the evaluation of events or situations that trigger individual emotions; Constructive refocusing refers to trying to change, redefine or explain the factual characteristics of events. For example, what behavior do we choose? What has happened? What can we learn from it? Rather than explaining the situation of the event. As one of the three factors of cognitive reconstruction, cognitive reappraisal has the characteristics of creative reconstruction. Engineering education professional certification is a specialized certification, which is implemented by professional certification institutions for all kinds of engineering majors in higher education.

**Subjects and Methods:** Based on professional certification, this paper analyzes the requirements of civil engineering talents training for civil engineering in application-oriented colleges and universities, analyzes the current situation and existing problems of civil engineering teachers in such colleges and universities, and construct a teaching staff construction plan to comprehensively improve the comprehensive quality of teachers in characteristic application-oriented colleges and universities.

**Results:** The results showed that through the investigation of the impact of emotional behavior, the guidance role of teachers was significantly positively correlated with their emotional regulation ability (P<0.01), indicating that the higher the degree of guidance, the stronger the emotional regulation ability of teachers. Emotional regulation. At the same time, the study found that the process of cognitive reappraisal is similar to that of insight problem solving, both of which require students to understand the problem situation again to better explain the event. An epiphany means that when a problem cannot be solved completely, a new idea and new heart appear suddenly due to a certain opportunity, so that the problem can be solved immediately. The theory of representation transformation of insight holds that the solution of insight requires individuals to change the inappropriate initial representation of the problem, and establish new and appropriate representation of the problem through the reconstruction of the problem, so as to solve the problem. This paper concludes that it is an effective talent policy for characteristic application-oriented colleges to strengthen the introduction and training of senior talents and play the backbone role of senior talents. Improving teachers’ teaching ability and optimizing their educational structure are effective ways to strengthen teachers’ educational literacy. Strengthening the joint training of school and enterprise and promoting teaching by scientific research are all good countermeasures to cultivate double -qualified teachers. It is feasible to pay attention to teachers’ career training, construct the evaluation system of teaching and scientific research, strengthen the evaluation of teachers and form the incentive mechanism of talents.

**Conclusions:** To build a satisfactory teaching staff with rich theoretical knowledge, excellent scientific research and high competitiveness, to ensure that the training of civil engineering professionals can meet the certification standards of engineering education, meet the needs of social development and adapt to the development of the industry.

**Acknowledgments**

The key project of higher education reform in Heilongjiang province, “Research and practice on the innovation of training mode for civil engineering professionals with characteristics and application based on professional certification” Project No: SJGZ20180032.

## LHMM070 RESEARCH ON SATISFACTION AND MENTAL HEALTH OF CIVIL SERVANTS’ COMPREHENSIVE MANAGEMENT PROFESSIONAL COMPETENCE IN WESTERN CHINA BASED ON THE INTEGRATION OF GOVERNMENT, INDUSTRY, EDUCATION AND RESEARCH

Ying Guo^a^, Cong Wang^a^, Ya Pan^a^, Yugang Jian^a^

^*a*^ *Department of Human Resources Management, Chongqing Institute of Engineering, Chongqing400056, China.*

**Background:** Facing the current situation of mental health of civil servants, in order to fully understand the current situation and needs of mental health of civil servants, and to improve the effectiveness of mental health assessment of civil servants, a data-driven mental health assessment method is proposed. In this method, the improved fuzzy clustering algorithm is used to make the elements in the sample belong to the same cluster by introducing uncertain membership degree. This method makes use of the memory storage function of uncertain membership degree to effectively reduce the number of calculation cycles. The 21st century is an era of urgent transformation of government functions. The concept of “human resource is the first resource” has been recognized by more and more people in government organizations. The training needs of government organizations for civil servants have gradually changed from the traditional cadre and personnel management system to the modern human resource management. It is an urgent task for government departments at all levels to cultivate a professional, efficient and pragmatic civil service team. The number of civil servants in general management is now the largest among the civil servants, and the jobs involved are also the largest. Their professional competence in work directly affects the ability of the public to feel the governance of government services, and also affects the public image of government organizations.

**Subjects and Methods:** At present, China’s civil service management system is basically still in the traditional cadre personnel management system, and has been accustomed to the traditional personnel management system. This can reasonably and accurately obtain relevant evaluation indicators and establish an evaluation indicator system. Combined with the fuzzy comprehensive evaluation model, the influence of fuzziness is fully considered, and the fuzzy weight is determined by the normalization method according to the different importance of each index. This can synthesize all fuzzy weights to get a comprehensive evaluation model, and realize the combination of qualitative evaluation and quantitative evaluation. The implementation of modern and scientific human resources management mode to manage civil servants is a difficult task, and the change of the idea that civil servants are also regarded as human resources is still in the stage of ideas and slogans. However, in the process of the existing civil servants serving the masses, it is found that the traditional personnel management system increasingly restricts the growth and long-term development of the civil servants. More and more government organizations are beginning to realize that only through high-quality civil servants can they gain social recognition and win people’s satisfaction. Because there are objective realities in the western region of China, such as complex management environment, difficult management, and less advanced management concepts, the importance of the professional competence and mental health of comprehensive management civil servants in the functional departments of governments at all levels in the western region cannot be ignored. By building the satisfaction index system of comprehensive management civil servants’ professional competence, this paper investigates the comprehensive management civil servants in western China.

**Results:** The concise scale ranges from 12 to 60. The opposite of high FNE is that it does not take advantage of others’ evaluation, and it is not necessarily expected or needs to be positive. The average score of 205 college students in the original table is 15.5 (SD=8.6), and the score is rectangular distribution. The average score of the other sample composed of 128 subjects is 13.6 (SD=7.6) The average score of the sample (n=150) used to prepare the 12-item concise scale is 35.7 (SD=8.1). This article finally proposes specific strategies to improve the professional competence and mental health status of comprehensive management civil servants in western China from the multiple perspectives of politics, industry, education and research. Through the analysis, it can study the satisfaction with their professional competence and mental health status.

**Conclusions:** Through this study, in order to provide reference for the development of civil servants’ human resources in other regions.

## LHMM071 RESEARCH ON THE DEVELOPMENT MECHANISM OF PHARMACEUTICAL CROSS BORDER E-COMMERCE AND ITS IMPACT ON THE HEALTH OF THE WHOLE PEOPLE

Hongxiang Ge^a^

^*a*^ *Huzhou Vocational & Technical College, Huzhou, Zhejiang 313099, China.*

**Background:** Mental health refers to that all aspects of psychology and activities are in a good or normal state. The ideal state of mental health is to maintain a state of good character, normal intelligence, correct cognition, proper emotion, reasonable will, positive attitude, proper behavior and good adaptation. The basic meaning of mental health is that all aspects of psychology and the process of activities are in a good or normal state. The ideal state of mental health is to maintain a state of perfect personality, normal intelligence, correct cognition, proper emotion, reasonable will, positive attitude, proper behavior and good adaptation. Corresponding to mental health are mental sub-health and mental illness. Mental health has different meanings from different perspectives, and its measurement standards are also different. With the improvement of health awareness, consumers have a strong demand for imported pharmaceuticals with new technologies and new ingredients. Cross-border e-commerce platforms can join forces with overseas brands, integrate upstream supply chain resources, and allow imported new pharmaceuticals to benefit consumers more quickly. With the liberalization of the prescription drug online sales policy, the future pharmaceutical e-commerce platform, as an important terminal for drug sales, will usher in considerable development. However, drugs are related to the life, health and safety of the people, and at the same time, the whole process of circulation must be achieved. traceable. Based on this, this paper sorts out the current development status of my country’s pharmaceutical e-commerce logistics, and systematically analyzes the special needs of pharmaceutical cross-border e-commerce distribution that are different from conventional logistics distribution according to the special attributes of drugs. On the basis of fully learning from the mature pharmaceutical logistics and distribution experience in developed countries such as the United States and Japan, the framework of the pharmaceutical distribution system based on the pharmaceutical e-commerce platform is proposed, and the operation process and supervision mechanism of the pharmaceutical logistics distribution system on this framework are explored.

**Subjects and Methods:** The purpose of this study is to understand the driving factors, policy supervision, market demand and other issues of the construction of cross-border pharmaceutical e-commerce platforms, and then put forward suggestions on how to resolve the challenges faced by the development of cross-border e-commerce, so as to promote cross-border pharmaceutical e-commerce The platform develops healthily. The demand forecast of the pharmaceutical e-commerce platform involves the properties of the drug itself and various promotional activities launched by the e-commerce platform. Based on the factors of patients’ demand for drugs in the large health environment, policy analysis methods, literature analysis methods, case studies and time series-Machine learning combination model, etc., to study the problems and challenges faced by the development of pharmaceutical e-commerce platforms. In the specific research, this paper takes a drug on the cross-border e-commerce platform of medicine as an example to carry out a case study. According to the regular sales of drugs in the promotion stage, according to the time-series data of the regular sales of each drug and the taking cycle, the influencing factors of the regular sales of drugs are analyzed to provide data reference for the design of the e-commerce platform. This paper also uses the multi-dimensional health status locus of control scale. From the perspective of the source of psychological control, MHLC assessed the view of health in three areas: the internality of Levenson, the influence of influential others and the role of opportunity. MHLC has two parallel versions, each of which is divided into three parts, and each part has six entries, all of which are self-evaluation scales. The score of each subscale ranges from 6-36. MHLC samples include healthy people such as students, secretaries, nurses, doctors, etc. Normal model data are in IHLC M-26 (SD=5), CHLC M=15 (SD-6), PHLC M=20 (SD=5.5). The internal consistency study shows that IHLC Cronbach a=0.61-0.80, CHLC) 0.55-0.83, PHLC 0.56-0.75, and the correlation of each component between the two parallel versions is IHLC0.48-0.77, CHLC0.38-0.65, and PHLC0.46-0.53. After an interval of 4 to 6 months, the three subscales of retest reliability correlation values were IHLCO.66 CHLC0.73 and PHLC 0.71 respectively.

**Results:** This study found that residents’ psychological quality was significantly positively correlated with cognitive reappraisal, and significantly negatively correlated with expression inhibition; The researchers believe that the development of metacognition is the basis of the development of residents’ emotion regulation strategies, while psychological quality consists of cognitive quality, personality quality and adaptability, and the core of cognitive quality is metacognition. In addition, the adaptability of psychological quality also includes emotional adaptation, which is closely related to emotional adjustment strategies. Therefore, psychological quality is closely related to emotion regulation strategies. This study also verified that cognitive reappraisal was significantly negatively correlated with negative indicators of mental health, while expression inhibition was significantly positively correlated with negative indicators of mental health. This paper draws the following results from the practical investigation and theoretical analysis: From the perspective of the development of cross-border pharmaceutical e-commerce, the problems faced are: consumers lack trust in pharmaceutical e-commerce platforms, worry about damage to physical and mental health, pharmaceutical logistics Uncertainty in distribution, lack of e-commerce talents in pharmaceutical companies, and inability of pharmaceutical companies to control prices. From the perspective of pharmaceutical cross-border e-commerce supervision, the existing problems are: difficult to grasp the boundaries of responsibilities, difficult to confirm the main responsibility, difficult to investigate illegal facts, and difficult to intervene in commodity supervision.

**Conclusions:** By analyzing the main elements of the pharmaceutical e-commerce service system and the relationship between the elements, discussing the construction of the pharmaceutical e-commerce service system, and putting forward countermeasures and suggestions to ensure the smooth operation of the pharmaceutical e-commerce service system: It is necessary to carry out full-cycle management of chronic diseases, promote The development of the pharmaceutical cold chain logistics industry, the establishment of a pharmaceutical e-commerce business system, the improvement of the pharmaceutical logistics distribution system, and the strengthening of the supervision of pharmaceutical e-commerce platforms, etc., will promote the standardized development of Internet+pharmaceutical e-commerce.

## LHMM072 RESEARCH ON THE REFORM OF BLENDED LEARNING OF APPLIED UNDERGRADUATE PUBLIC BASIC COURSES UNDER THE INFORMATION CONDITION BASED ON THE PERSPECTIVE OF STUDENTS’ MENTAL HEALTH

Haijun Cao^a^, Li Li^a^

^*a*^ *Shandong Jiaotong University, Jinan, 250023, China.*

**Background:** Mental health refers to a continuous and positive psychological state, in which the subject can make good adaptation and give full play to its physical and mental potential. Mental health education is an important part of the “new health education.” It aims to cultivate healthy social citizens in both body and mind. Through the use of health management methods, it focuses on the improvement of the campus environment and functional environment, and cooperates with the improvement of the humanistic environment, and provides scientific, healthy and professional guidance with teachers and students as the main body. Faced with the challenges in the teaching implementation and teaching management of the public basic courses in colleges and universities under normal epidemic prevention in China, this paper analyzes the specific learning situations of students in application-oriented undergraduate colleges and universities, such as their psychological state and learning ability, the reality that the public basic courses are given priority to large-class instruction, and the needs of multi-scene teaching under the normal situation of epidemic prevention and control, in view of the implementation plan put forward by China in the education reform to cultivate all-round development of application-oriented talents, comprehensively construct the “golden courses” in the undergraduate teaching, and eliminate the “frivolous courses.”

**Subjects and Methods:** The relevant academic documents from 1990 to 2022 in the database of Science Network are selected as the data base. Based on the scientific measurement method and with the help of information visualization CiteSpace software, the map quantitative analysis of academic papers in the field of mental health is carried out. The two dimensions of the locus of control scale were used as measurement indicators. Based on the case study of Advanced mathematics course as a public basic course, this paper expounds in detail how to make full use of information-based teaching methods in public basic courses: for example, the “light integration” of online and offline SPOC mode of multi-scene integration, the integration of mathematics and professional courses, and the integration of mathematics and ideological and political education with the help of “Rain classroom,” etc., to continuously improve the quality of classroom teaching, to evaluate students’ learning effect in the whole process and in multiple dimensions, and to construct an effective “first-class course.”

**Results:** The preparation of the scale can be seen in the special report of Rotter. The version introduced here contains 23 items and 6 inserted questions. Each item is a group of internal control statements and external control statements. The subjects are required to choose one of them. The external control selection score ranges from 0 (extreme internal control) to 23 (extreme external control). The scale is a self-assessment scale, which is completed within 15 minutes. It is most commonly used for college students and other groups. It can be seen from several studies that the norms of the I-E scale are different. Owens (1966) reported that men are 8 2 (SD=4.0) female=8.5 (SD=3.9) Parkes (1985) reported male (n=146) X=11 6 (SD=3. 3) Female (n=260) X=12.6 (SD=3.7). Based on the teaching mode of Advanced Mathematics course, the implementation strategy of how to carry out online and offline multi-scene blended learning based on students’ psychological characteristics and student-centeredness in the public basic course of large class teaching was illustrated. The information technology tool, “Rain Classroom” is deeply applied in the teaching process of Advanced Mathematics, which has changed the “cramming education” teaching mode with blackboard and multimedia. The SPOC-based online and offline blended teaching mode and the remote students’ integrated live teaching mode are adopted, and the teaching design with pertinence and accuracy and the whole process and multi-dimensional assessment are implemented to be “student-centered.”

**Conclusions:** Since the advantages of the blended learning mode can change the traditional “spoon-feeding” teaching mode, the reform of the blended learning mode should be carried out under the support of the Internet information technology to continuously improve the classroom and better improve the teaching quality.

**Acknowledgements**

Supported by the Shandong Undergraduate Teaching Reform Research Project (No. M2021164), Special topic of theoretical research on the construction of application-oriented undergraduate colleges and universities of Shandong Jiaotong University, (No. YYX20211228).

## LHMM073 COMMENTS ON CHINA’S PUBLIC HEALTH EVENTS BY THE MAINSTREAM ENGLISH MEDIA IN INDIA FROM THE PERSPECTIVE OF PSYCHOLOGICAL HEALTH IN DIPLOMATIC BEHAVIOR

Shan Tang^a^

^*a*^ *School of Foreign Languages& Cultures, Guangdong University of Finance, Guangzhou, Guangdong 510521, China.*

**Background:** With the development of society and the progress of the times, more and more people pay attention to the psychological health of journalists. Mental health education is a very important part of quality education in China, because mental health can enable journalists to face life with a positive attitude and play a positive role in their study and life. Mental health education will also have a certain impact on the norms of behavior of journalists, mainly because mental health can promote the good behavior of journalists and further play a positive role in the healthy growth of journalists. Sino-Indian relations have plunged to an all-time low since the military standoff in Galwan Valley. And the coronavirus pandemic has further acted as a “game changer” as it accelerated growing mistrust and feeling of a threat now persisting between the two superpowers. During this process, the Indian media, especially the mainstream English-language media, has long manipulated public opinion with strong hostility toward China, helping to shape the agenda for India’s foreign policy toward China. The Indian media should be partially responsible for the deteriorating Sino-Indian relations as well as the increasing antagonism toward China among Indian people. Based on an interdisciplinary perspective with the help of corpus-driven critical discourse analysis, this paper explores the portrayal of China in pandemic narratives from the viewpoint of Indian mainstream English-language media.

**Subjects and Methods:** The Social Support Scale (PSSS) was used in this study. Social support is regarded as one of the important mediating factors that determine the relationship between psychological stress and health. Social support refers to the spiritual and material help and support given to individuals from various aspects of society, including families, relatives, friends, colleagues, partners, party and league organizations, trade unions and other organizations, reflecting the close degree and quality of a person’s relationship with society. However, various researchers have different understanding of this from their respective theories and research purposes, and their classification also has diversity. At present, it can be roughly divided into two categories, one is objective, practical or visible support, including material direct assistance and social network, and the other is subjective experience of support, which refers to the emotional experience and/or satisfaction of individuals feeling respected, supported and understood in society. Many authors emphasize that the effect of social support is consistent with the perceived level of support. The Perceived Social Support Scale (PSSS) introduced by Blumenthal and compiled by Zimet et al. is a social support scale that emphasizes individual self-understanding and self-perception. It measures the degree of support that individuals perceive from various sources of social support, such as family, friends and others, and reflects the total degree of social support that individuals feel with the total score. Data for this study was gathered from scratch utilizing the Factiva massive news database. A total of 941 news articles on coronavirus situation in China from three Indian English-language newspapers, namely The Times of India, Hindustan Times and The Hindu respectively, with dates ranging from January 1st to August 24th 2022, were extracted by setting “China and coronavirus,” “China and virus” and “China and pandemic” as the keywords. Given that it is impossible to process all of these texts by one person, a valid corpus consisting of 141 news items was created after manually checking the data’s relevance and excluding duplicated materials.

**Results:** There was a significant positive correlation between news diplomatic behavior and emotional regulation ability (P<0.01). Network analysis showed that the relationship between state anxiety and adolescent trait anxiety (r=0.63), state anxiety and trait anxiety (r=0.56), and state anxiety and trait anxiety (r=0.53) was the strongest. At the same time, the emotional anxiety of news reports is positively correlated with the state anxiety of journalists (r=0.28), the trait anxiety of news reports is positively correlated with the trait anxiety of journalists (r=0.16), and the emotional anxiety of news reports is positively correlated with the state anxiety (r=0.09). Trait anxiety (r=0.05) and state anxiety (r=0.06) were also positively correlated with emotional anxiety in news reports. It was found that there is a greater tendency among Indian media to construct China as a “cold and ruthless authoritarian state” during the pandemic and took the moral high ground against China to question the efficacy of China’s pandemic control strategy filled with misinformation and conspiracy theories.

**Conclusions:** The frequently distorted or exaggerated facts used by some Indian media to tarnish China’s efforts in pandemic to incite public hostility toward China should raise wider concern in Chinese society. These negative factors can kidnap Indian public opinions and government decision-making and greatly undermine the deeply held international solidarity and trust among nations while fostering hatred and stigmatization, if without proper counterattack and refutation. Consequently, both Chinese and Indian media have the responsibility to present a genuine image of bilateral relations and give the right perspective to reveal the true status quo of pandemic in China to the outside world.

**Acknowledgments**

This work was supported by project grants from the 2020 Guangdong Province Philosophy and Social Science Planning Project “Indians and Hong Kong Society: Economic Impact and Cultural Characteristics” (Grant No. GD20YTQ02) and 2022 Guangzhou City philosophy and Social Science development Annual Project “The Impact of Language Issues on the National Identity of Hong Kong People” (Grant No. 2022GZGJ235).

## LHMM074 EFFICIENCY EVALUATION AND SPATIAL DIFFERENCE OF INFRASTRUCTURE INVESTMENT: ANALYSIS OF UNEXPECTED FACTORS AND EMOTIONAL PSYCHOLOGICAL FACTORS BASED ON SBM

Qin Zhang^a^, Guanglong Yue^a^, Xinping Yu^a^, Chongyu He^a,b^, Yinzi Bao^a^, Chen Chen^c^

^*a*^ *Business School, Ningbo University, Ningbo 315211, China,* ^*b*^ *YangMing School, Ningbo University, Ningbo 315211, China,* ^*c*^ *The School of Management, Huazhong University of Science and Technology, Wuhan 430074, China.*

**Background:** Emotion is different from cognition. It is related to the personal needs and subjective attitudes of individuals. From this connection, we can derive two special forms of emotion, one is the internal state or experience, and the other is the explicit expression, which is a feature that the cognitive process does not have. Therefore, emotion and cognition are causal and concomitant. Emotions can initiate, interfere, organize or be destroyed in cognitive processes and behaviors. Cognitive evaluation of things can initiate, transfer or change emotional reactions and experiences. Infrastructure investment is an important measure used by the government to stabilize the economy. Good infrastructure can not only reduce social production costs, but also improve social production efficiency and improve people’s quality of life. In recent years, due to self-attribution bias, infrastructure investment has been unbalanced and uncoordinated. How to break away from the development mode of high investment and high consumption of infrastructure in the past and transform it to high-quality sustainable development is a problem that needs to be solved urgently. The key to solving this problem is to improve the utilization rate through efficiency governance. To assess the investment benefits of infrastructure scientific and effective, carry out spatial analysis, and thus improve the efficiency of investment and construction, mitigate efficiency deviation and make investors more rational through continuous learning and improvement.

**Subjects and Methods:** Using the revised lin shangping’s “Organization’s emotional labor burden scale” And “Minnesota satisfaction scale (msq), the study distributed 207 questionnaires to the construction project manager of a contractor undertaking a project, and 207 were recovered. The sample structure distribution was that male accounted for 51.7% and the age was 25 years old (inclusive) the following proportion is the lowest, accounting for 2.5%, and the proportion over 36 years old is the highest, accounting for 62.6%. As for education level, the proportion of universities (colleges and universities) is the highest, accounting for 64.0%. In terms of service years, the proportion of 1-3 years is the lowest, accounting for 4.4%, and the proportion of more than 7 years is the highest, accounting for 84.2%. The total reliability of items in the questionnaire and the total inconsistency of items in the questionnaire are analyzed by means of correction, so as to improve the total reliability of items in the questionnaire. Regression analysis was used to verify the three hypotheses. Taking ecological benefits outputs into the composition of index system, given the impact of infrastructure investment on the environment, the ecological benefit output is included in the indicator system, and the unexpected SBM model is used to evaluate the infrastructure investment efficiency of 31 provinces (municipalities, autonomous regions) in China to guide investors to make rational infrastructure investment decisions. Based on the efficiency value of infrastructure investment in China, the efficiency of infrastructure investment is studied from the aspects of global spatial autocorrelation and local spatial autocorrelation. The spatial correlation and evolution of its distribution are further systematically analyzed by using spatial autocorrelation methods such as ArcGIS and Geoda, and the spatial distribution characteristics of infrastructure investment efficiency in China are deeply explored.

**Results:** This paper takes emotional regulation as the independent variable and investment pressure as the dependent variable to explore the impact of emotional regulation on investment pressure. This paper takes emotional regulation as the independent variable and investment satisfaction as the dependent variable to explore the impact of emotional regulation on investment satisfaction. According to the measurement results, f value is 28.667, r2 is 0.129, p value is 0.000<0.001, β A positive value indicates that emotional regulation has a positive and significant impact on investment satisfaction. This is consistent with the hypothesis of this study, and based on this, we can establish an investment sentiment regulation model. Firstly, in 2017-2021, China’s infrastructure investment efficiency is generally at a low level, which needs to be improved to a large extent, and there is a large gap between the eastern and central-western infrastructure investment efficiency. The efficiency of infrastructure investment shows a decreasing trend in the east, middle and west. Secondly, from the overall Moran’s I index, there is a significant positive spatial autocorrelation of infrastructure investment efficiency in each region, and the spatial correlation of infrastructure investment benefits in each province is still very low. Thirdly, from the LISA agglomeration plot and significance level plot, the spatial distribution of infrastructure investment efficiency in China has been relatively stable, and there has been no transition from one quadrant to another. Most provinces have high spatial stability in infrastructure investment efficiency.

**Conclusions:** To increase the efficiency of infrastructure investment, firstly, we should continuously increase the investment in infrastructure and reasonably allocate all kinds of resources. Secondly, interregional cooperation and coordinated development should be strengthened. Third, we should strengthen the infrastructure construction and technological innovation of enterprises.

**Acknowledgements**

This work was supported by the Longyuan Construction Finance Institute of Ningbo University Project “Research on Mode, Efficiency and Risk Prevention of Private Capital Participating in Infrastructure Investment” under Grant [LYZDA2004].

## LHMM075 AN INFLUENCE OF PERSONALITY TRAITS ON MENTAL HEALTH OF ONLY-CHILD COLLEGE STUDENTS: THE MEDIATING ROLE OF FAMILY SUPPORT AND THE MODERATING ROLE OF REGULATORY FOCUS

Yanhong Wang^a^, Yixia Cong^b^

^*a*^ *Psychological Counseling Center, YANG-EN University, Quanzhou, Fujian 362014, China,* ^*b*^ *Foreign Language School, Xiamen Nanyang Vocational College, Xiamen, Fujian 361101, China.*

**Objective:** The outbreak of COVID-19 has changed the way college students study and live, putting them under great psychological pressure and causing more anxiety, depression, insomnia, and other adverse states, which threaten their mental health. To explore the mediating role of family support in the relationship between personality traits and mental health of only-child college students and the moderating role of regulatory focus.

**Methods:** A survey was used with the Short and extra-short forms of the big five Inventory–2, family support questionnaire, regulatory focus questionnaire, and a five-item version of the mental health inventory among 600 only-child in Quanzhou. 572 questionnaires were effectively collected with an effective recovery rate of 95.33%. SPSS22.0 was used for the hierarchical regression test and PROCESS model 14 was used for the bootstrap test.

**Results:** (1) The five dimensions of extraversion, agreeableness, conscientiousness, openness and neuroticism had significant predictive effects on family support (β=0.39, SE=0.08, P <0.001; β=0.59, SE=0.08, P <0.001; β=0.57, SE=0.08, P <0.001; β=0.31, SE=0.08, P <0.001); β=-0.49, SE=0.07, P <0.001), for regulatory focus promotion dimension, family support positively predictive effects on mental health (β=0.50, SE=0.09, P <0.001; β=0.43, SE=0.08, P <0.001; β=0.45, SE=0.08, P <0.001; β=0.49, SE=0.09, P <0.001; β=0.39, SE=0.08, P <0.001); for the regulatory focus prevention dimension, family support positively predictive effects on mental health (β=0.47, SE=0.08, P <0.001; β=0.39, SE=0.08, P <0.001; β=0.39, SE=0.08, P <0.001; β=0.45, SE=0.09, P <0.001; β=0.32, SE=0.07, P <0.001). That is, the big five personality indirectly affects mental health through the mediating role of family support; (2) The interaction terms of regulatory focus promotion and family support negatively predicted mental health, respectively (β=-0.06, SE=0.02, P <0.01; β=-0.05, SE=0.02, P <0.01; β=-0.05, SE=0.02, P <0.01; β=-0.05, SE=0.02, P <0.01; β=-0.05, SE=0.02, P <0.01); The interaction terms of regulatory focus prevention and family support negatively predicted mental health, respectively(β=-0.05, SE=0.02, P <0.01; β=-0.04, SE=0.02, P <0.01; β=-0.04, SE=0.02, P <0.01; β=-0.05, SE=0.02, P <0.01; β=-0.05, SE=0.02, P >0.05).That is, the moderating effect of regulatory focus on the relationship between family support and mental health was significant.

**Conclusion:** In this study, we built a moderated mediation model to investigate the interaction mechanism and boundary conditions between big Five personality and only-child mental health, and to explore the mediating role of family support in this process and the moderating role of regulatory focus in this mediation path. The research results are helpful to improve the mental health level of only-child college students from multiple perspectives and levels, and also provide scientific reference for the prevention and intervention of the only child psychological crisis under the normalization of the epidemic.

**Acknowledgements**

The national social science fund pedagogy project “The Generation Mechanism and intervention countermeasure research of Vocational College Students’ academic burnout under the background of higher education popularization” (Project No. BIA220072).

## LHMM076 A STUDY ON THE INFLUENCE OF SALESMAN’S FACE TRUST ON CONSUMERS’ PURCHASE INTENTION AND BEHAVIORAL PSYCHOLOGY

Anbo Wu^a,b,c^, Yuqin He^a,b^

^*a*^ *School of Management, Xi’an University of Science and Technology, Xi’an 710054, Shaanxi, China,* ^*b*^ *Energy Economic Research Center, Xi’an University of Science and Technology, Xi’an 710054, Shaanxi, China,* ^*c*^ *Human Factors and Management Ergonomics Research Center, Xi’an University of Science and Technology, Xi’an 710054, Shaanxi, China.*

**Background:** In an age of “face recognition,” consumers tend to attach greater importance to the face of the salesperson when purchasing goods, namely their preference for the face of the salesperson may influence their final purchase intention in manner of psychology. Whether a salesperson is a guide in an offline physical shop or a streamer for online sales, the face is commonly presented to the consumer along with the product. As an important evaluation of faces, face trust can psychologically arouse consumers’ emotions and thus induce them to purchase. From the perspective of complex system evolution, combined with the relevant theories of micro behavioral psychology, this paper further discusses the impact of four typical complex network structure evolution on sales transfer behavior and knowledge level of knowledge subject, as well as the change process of the impact on emotional behavior.

**Subjects and Methods:** By reviewing the domestic and international researches on face trust, gender, expression, face shape, facial features and face typicality are selected as 5 dimensions of face trust to investigate the specific effects on purchase intention and psychology. Risk perception is also introduced as a mediator variable while the product type is introduced as a moderator variable to examine the role of these two variables in the overall study. The study is conducted in the form of an online+offline questionnaire. After collecting the questionnaires, SPSS is used to statistically analyse the information of the sample. And the data collected are analyzed to test the hypotheses proposed: (1)the trust in a salesperson’s face has a significant positive effect on consumers’ purchase intentions and psychology; (2)risk perception mediates the relationship between trust in the faces of salespeople and consumers’ willingness to purchase; (3)product type can significantly moderate the effect of a salesperson’s gender on consumer risk perception. This paper designs a simulation research process based on complex knowledge network model. The calculation index mainly includes three aspects: network value, node coupling cost and the weight of Relationship Edge. Node coupling cost consists of three dimensions: psychological distance cost between network nodes, cooperation value difference between network nodes and connectivity difference between network nodes. Through programming, four programs are used to simulate and analyze the evolution of complex network structure based on knowledge subject coupling cost, psychological expectation and behavior change. At the same time, in order to verify the impact of spatial layout on consumers’ emotion, this study uses relevant scales to investigate. (1) Positive emotion scale. The Panas emotion scale developed by Wason and others is widely used to measure emotion. The scale includes two dimensions of positive emotion and negative emotion as measurement indicators. There are 6 questions in this dimension, which are scored by Likert 5 points (1 means “very inconsistent,” 5 means “very consistent,” the same below). In this study, the clonbach coefficient of the questionnaire is 0.90. (2) Motivation scale. The topic of measuring motivation mainly refers to the entrepreneurial motivation scale compiled by Phan, which has 8 questions and adopts Likert’s 5-point scoring. (3) Social support scale. The scale is adapted from the social support scale prepared by Ye Yuemei and others. It has 8 questions and is scored by Likert 5 points. Clone Bach of the scale α The coefficient is 0.87. (4) Behavioral propensity scale. Entrepreneurial orientation dimension in intention measurement. The scale has 6 questions and is scored by Likert 5 points. The clonbach coefficient of the scale is 0.95.

**Results:** The results indicate that: (1) face trust has a significant positive effect on purchase intention and psychology; (2) risk perception partially mediates the effect of salesperson face trust on consumer purchase intention and psychology; and (3) product type serves as a significant moderator in the effect of face trust on consumer risk perception.

**Conclusions:** Face trust has a direct impact on purchase intention and psychology. Salespersons can increase consumers’ purchase intention by reducing their level of risk perception. And product type can moderate the effect of most dimensions of face trust on purchase intentions. By placing the most effective salespeople in the most appropriate positions, it is possible to take advantage of the consumer’s “face-concerned consumption” mentality to reduce their risk perception level, thereby increasing their trust in the salesperson and the merchant, enhancing their purchasing loyalty and further improving the merchant’s profitability.

**Acknowledgements**

This research was supported by the Special Scientific Research Plan of the Education Department of Shaanxi Provincial Government -- Humanities and Social Science (Grant No. 20JK0232).

## LHMM077 TRANSFORMATION OF CHINESE SPORTS TOURISM CONSUMPTION FORM FROM THE PERSPECTIVE OF BEHAVIORAL MENTAL HEALTH

Wenxing Liu^a,b^, Yangbo Guo^b^

^*a*^ *Institute of Sport, Yanching Institute of Technology, Hebei 065200, China,* ^*b*^ *Harbin Sport University, Harbin 150001, China.*

**Background:** The perception behavior of knowledge subject can get some feedback and inspiration from the trend change of knowledge level. The change of knowledge subject’s psychological expectation and behavior is of great significance to the evolution and utilization of knowledge network structure in complex systems. At present, the research on inter-organizational knowledge flow and knowledge diffusion based on network structure is relatively mature. From the perspective of complex system evolution, combined with the relevant theories of micro behavioral psychology, this paper further discusses the impact of four typical complex network structure evolution on the knowledge transfer behavior and knowledge level of knowledge subjects, as well as the change process of network structure. Influence of emotional behavior. The global spread of the coronavirus has dealt an unprecedented blow to multiple industries, and the sports industry, with its typical aggregation characteristics, has been affected inevitably. Sports tourism as a form of sports industry, not only can drive the development of sports industry, but also has the effect of promoting the local traditional culture. The Chinese government has gradually introduced various sports tourism policies since 2015 to accelerate the economic development of industries in various regions and to promote the development of traditional sports in China.

**Subjects and Methods:** The purpose of using the “life event scale” in this study is to conduct a qualitative and quantitative investigation of the perceived degree of events. The theoretical assumption of SRRS is that any form of life change requires individuals to mobilize the stress resources of the body for new adaptation, thus causing tension. The calculation method of SRRS is to make a weighted score on the basis of the cumulative number of life events, that is, give different scores to different life events, and then accumulate their total value. SRRS weighting is based on a norm of 5000 people. The respondents took the event as the standard and evaluated the scores of other life events according to their direct or indirect experience. Then calculate the average value of each event (5000 people), divide the average value by 10, and take its integer as the standardized score of the event. The research methods of literature, fieldwork, questionnaires, and mathematical statistics were used to study the changes in the form of sports tourism and changes in consumer behavior in China from 2019-2022, and to explore the changes in the form of sports tourism consumption in China and the changes in consumer behavior affected by the epidemic.

**Results:** Carrying out correlation analysis on the inspection results, and the correlation coefficient is 0.828. The results showed that BAI was significantly positively correlated with the total score of SAS objective evaluation. To prove the effectiveness of BAI in the application of psychological assessment. The results of sensitivity and specificity are as follows: BAI≥40 and ≥45 were taken as positive, and the false positive rate, false negative rate, sensitivity and specificity were obtained to evaluate the evaluation effect of BAI on anxiety. The results showed that the sensitivity (91.66%) and specificity (91.25%) of BSI were relatively balanced when the BSI was ≥ 45. Since the beginning of the epidemic, numerous changes have occurred in the consumption behavior of Chinese sports tourism consumers, firstly, there has been a contradiction between rising consumption intentions and actual lower consumption, secondly, consumption motives have become stronger, furthermore, the long period of silent management in some areas has led to blind consumption by sports tourism consumers in that area, and finally, the form of consumption has changed from the previous offline consumption to online virtual consumption. Due to the impact of the epidemic, there has been an unprecedented shift in the form of sports tourism in China, with some tourist attractions launching VR virtual reality tourism programs, others officially using various short video software and live streaming software to promote them, and some even launching online on-demand measures for attractions or sports programs.

**Conclusions:** In the face of the sudden epidemic, China has responded more effectively in terms of sports tourism, transforming the traditional forms of sports tourism and gradually accepting a change in consumer behavior. However, some new contradictions have emerged, including the contradiction between consumption and experience, between policy promotion and actual development, between consumer desire and consumption, and between the cost of innovative forms of sports tourism and consumer satisfaction.

## LHMM078 INNOVATIVE RESEARCH ON THE CULTIVATION MODE OF CIVIL ENGINEERING PROFESSIONALS IN CHARACTERISTIC APPLICATION-ORIENTED UNIVERSITIES AND ITS INFLUENCE ON STUDENTS’ EMOTIONAL REGULATION BASED ON PROFESSIONAL CERTIFICATION

Rui Pan^a^, Jixiang Liao^a^, Shaonan Han^a^, Dayong Wang^a^, Jingbo Yu^b^, Peifeng Cheng^c^

^*a*^ *Harbin University, Heilongjiang, Harbin, 150086, China,* ^*b*^ *Heilongjiang University of Engineering, Heilongjiang, Harbin, 150050, China,* ^*c*^ *Northeast Forestry University, Heilongjiang, Harbin, 150040, China.*

**Background:** Professional certification provides convenient conditions for the optimal allocation of various innovative elements in university innovation activities. At the same time, the application of professional certification also plays an important role in improving the efficiency of innovation activities and emotional regulation. The professional certification of engineering cultivation is a kind of specialized certification in the field of higher cultivation, which aims to improve the cultivation quality of engineering talents in colleges. Adhering to the three basic concepts of “student-centered, output-oriented and continuous improvement,” it mainly evaluates the training objectives, quality, teaching staff, curriculum, experimental equipment, teaching management and teaching documents of major students.

**Subjects and Methods:** In order to verify the impact of college students’ innovation on emotion, this study used the relevant scale and the positive emotion scale to investigate. The Panas Emotion Scale developed by Watson et al. is widely used to measure emotions. The scale includes two dimensions: positive emotion and negative emotion. The samples are from full-time master’s and undergraduate students in a university. 210 students (1 graduate class, 4 undergraduate classes) were randomly divided into 46 teams with an average size of 4.57. The age of the subjects ranged from 19 to 25 years old, with an average of 21.45 years old. Women accounted for 76.1% of the total sample, and men accounted for 23.9%. All the subjects were required to complete a campus recruitment project in the form of a team simulating the employer in about two months, which was designed according to the requirements of the course “Talent Evaluation.” Before the formal implementation of the project, the subjects were randomly divided into groups. The implementation cycle of the whole project is 9-11 weeks (two classes per week, 90 minutes in total), including three main links: project preparation, project implementation and project summary. Among them, the preparation phase lasts 2-3 weeks, mainly completing the tasks of formulating the overall recruitment plan, determining the recruitment target position, setting the post evaluation model, designing the evaluation technical scheme, preparing the test questions and evaluation forms. The implementation phase lasts for 3-4 weeks, mainly completing the tasks of publishing recruitment information, collecting and screening resumes, informing interviews, organizing interviews, and grading examiners. The summary phase lasts 3-4 weeks, mainly completing the discussion and summary of candidates’ evaluation results, issuance of evaluation reports, project reports, and submission of final reports. During the whole project implementation process, in addition to completing the corresponding project tasks in class, the project team also needs to make sufficient preparations and carry out more interaction and discussion after class. Based on professional certification and aiming at civil engineering majors in characteristic application-oriented universities, this paper analyzes the existing problems and causes of talent training, and puts forward the innovative mode of talent training.

**Results:** In the aspect of emotion regulation ability, in order to test the structural validity of the main variables in the study, the model comparison was conducted first. Using Mplus6.11 software, we have done confirmatory factor analysis on 12 dimensional models (5 personality dimensions, 4 emotional intelligence dimensions, team trust perception, task performance, organizational citizenship behavior, 48 items in total), 8 dimensional models (5 personality dimensions combined), and 9 dimensional models (4 emotional intelligence dimensions combined) at the item level. The analysis results show that the main fitting index values of the 12 dimensional models are: x2 (1014)=1584.10, x2/df=1.56, RMSEA=0,05, CFI=0.89, which is better than the 8 dimensional model (x2 (1052)=2383.22, X2/df=2.27, RMSEA=0.08, CFI=0.74) and the 9 dimensional model (x2 (1044)=2620.93, 2/df=2.51, RMSEA=0.09, CFI=0.70). Secondly, the factor load of each item in the optimal model is investigated. From the Mplus6.11 analysis results, the factor load is 0.23-0.94 (the factor load of the lowest is 0.23, and the factor load of the other 47 items is greater than 0.40), and all are significant at the level of 0,01. The above results show that the measurement variables in this study have good structural validity. The reliability of each measurement tool basically meets the requirements of psychometrics. From the results of reliability and validity test, the data collected by each measurement tool in this study has strong reliability. Making clear the relationship between major positioning and talent training objectives, build talent training programs and practical teaching systems, enhance students’ core competence, improve undergraduates’ awareness of innovation, explore the integration of curriculum ideology and engineering education certification, construct the teaching quality monitoring and guarantee system. All of these are effective methods to improve the training level of civil engineering specialty in characteristic application-oriented undergraduate.

**Conclusions:** civil engineering majors in characteristic application-oriented universities should combine their own conditions, grasp the core content of professional certification, build an innovative mode of talent training, constantly improve the training quality of application-oriented talents, meet the needs of national social and educational development, and adapt to the development of industries and workplace needs.

**Acknowledgments**

The key project of higher education reform in Heilongjiang province, “Research and practice on the innovation of training mode for civil engineering professionals with characteristics and application based on professional certification” Project No: SJGZ20180032.

## LHMM079 EXPLORING THE CHANGE OF STUDENTS’ PSYCHOLOGICAL MOOD AND TEACHING CONFIGURATION IN THE ONLINE AND OFFLINE HYBRID INTRODUCTORY COURSE: TAKING URBAN PLANNING AS AN EXAMPLE

Ming Sun^a^, Peng Cui ^a^, Haihong Song ^a^

^*a*^ *School of Landscape, Northeast Forestry University, Harbin, 150040, China.*

**Background:** The teaching courses based on educational psychology should not only pay attention to traditional off-line classroom teaching, strive to create a strong atmosphere for off-line teaching, but also enrich the on-line platform using various modern network technologies. During the epidemic situation, both teachers and students are under the stress of epidemic situation. It is urgent to explore the teaching methods of introductory courses in Colleges and universities.

**Methods:** 200 students from Northeast Forestry University were selected as the research subjects. Study the correlation between anxiety, self-efficacy and urban planning curriculum performance. Final exam scores are used to divide participants into pass and fail groups. Both gender and major are considered as variables of achievement. This study includes four elements of self-efficacy: learning ability, courage to overcome challenges, efforts and overcoming setbacks. Through t-test, univariate and multivariate analysis of variance and regression analysis, we investigated the relationship between anxiety, self-efficacy and academic performance of students of different majors and genders. We used the introduction lesson on urban-rural planning of Northeast forestry university in China as a case example and explores the teaching way around the graduation requirement. Through online and offline mixed teaching perspectives, the outline of the inquiry course, changed the teaching objectives, improved the course outline, reversed teaching, and flipped classroom methods.

**Result:** In learning, expression inhibition has a significant positive predictive effect on implicit positive emotion, and the interaction between interpersonal emotion regulation and regulation difficulty has a significant negative predictive effect on implicit positive emotion, indicating that expression inhibition plays a part of intermediary role in the influence of interpersonal emotion regulation on implicit positive emotion. The results of simple slope test showed that the mediating effect of expression inhibition was significant when M-1SD was selected for mood regulation difficulties. The 95% confidence interval of Bootstrap test is [0.01, 0.04], excluding 0. When M+1SD was taken, the mediating effect of expression inhibition was significant. The 95% confidence interval of Bootstrap test is [0.01, 0.05], excluding 0. The expression inhibition plays a significant mediating role in regulating the high and low levels of difficulty. The introductory course should adapt to the psychological changes of students and adopt the mixed teaching mode, improve the teaching outline and update the mixed teaching mode around the graduation requirements and teaching objectives. Firstly, there is a significant correlation between the improvement of the outline of the introduction course and the graduation requirements. Each class has a lecture session, which is conducive to clearly showing the indicators of the graduation requirements involved. Secondly, based on students’ learning psychology, increasing or decreasing the allocation of teaching time in and after class is an effective mixed teaching method. Increasing classroom activities, teaching online after class, and reducing classroom teaching practices and homework can promote online and offline hybrid teaching. Thirdly, the paper verifies the teaching effect of teaching and learning-centered introduction course from eight dimensions. Course data take new students teaching in urban and rural planning as practice objects. Finally, the paper puts forward new improvement plans for the teaching problems, which may be more conducive to promoting the achievement of the objectives of the graduation requirements in the Introduction Course.

**Conclusion:** The analysis of the direct effects of different levels of emotional regulation difficulties and the intermediary effects of expression inhibition shows that. The direct effect of interpersonal emotion regulation decreases with the increase of regulation difficulty. That is, as the level of difficulty in regulation decreases, interpersonal emotion regulation is more likely to directly affect implicit positive emotion. The amount of mediating effect of expression inhibition increased with the increase of regulation difficulty. That is, as the level of regulation difficulty increases, learning emotion regulation is easier to predict implicit positive indirectly through expression inhibition. From the students’ psychological state, course framework, teaching design, teaching effect and improvement plan, the paper comes to the conclusion. This provides a reference for the professional introduction course.

**Acknowledgments**

This work was supported by Educational and Teaching Research Project of Northeast Forestry University teaching (DGY2022-32); The 2021 Key Project of Education Science Planning, Heilongjiang, China (GJB1421230).

## LHMM080 EXPERIMENTAL STUDY ON THE EFFECT OF BFR LOW INTENSITY TRAINING ON THE STRENGTH QUALITY OF CUBA ATHLETES

Lancang Wang

^*a*^ *Beijing University of Chemical Technology, Beijing, China.*

**Background:** Strength training is an essential part of basketball training, but high-intensity strength training is not only easy to cause injuries to athletes, but also affects the high-quality completion of other training. The full name of BFR training is Blood Flow Restriction Training, which restricts blood flow by binding a compression band at the root of the limbs with a compression device, and then combines with general strength training. This paper aims to study the effect of improving CUBA (Chinese University Basketball Association) players’ strength training through BFR training under the condition of reducing training intensity and injury risk.

**Subjects and Methods:** This paper takes 20 men’s basketball players of CUBA (Chinese University Basketball Association) of Beijing University of Chemical Technology as research objects, and studies the application of BFR training in the strength training of basketball players through literature, expert interview, mathematical statistics, teaching experiment and other research methods. The experiment was divided into two groups, the control group used traditional strength training methods, and the experimental group used BFR training. Before and after the experiment, measure and record all indicators of the subjects, and select two indicators for strength quality: muscle endurance (maximum number of single self weight squat, pull up, and push- up) and maximum strength (maximum weight of single squat, bench press hard drawing).

**Results:** The study showed that the indexes of muscle endurance and maximum strength in the control group were significantly improved before and after the experiment, with extremely significant differences (P<0.01); The indexes of muscle endurance and maximum strength in the experimental group were significantly improved before and after the experiment, with extremely significant differences (P<0.01). After the comparison between groups, there were significant differences in three indexes of muscle endurance between the control group and the experimental group (P<0.05), and there was no significant difference in three indexes of maximum strength (P>0.05).

**Conclusions:** The effect of BFR training on improving muscle endurance is better than that of traditional strength training, but whether the maximum strength improvement is more obvious than that of traditional quantity training may vary from person to person. BFR training equipment is simple and easy to operate in practical application. It has the characteristics of low intensity and low injury risk in strength training, and can be an important supplement to traditional strength training.

## LHMM081 RESEARCH ON THE DEVELOPMENT OF INTEGRATING IDEOLOGICAL, POLITICAL AND PSYCHOLOGICAL HEALTH EDUCATION INTO “FILM AND TV CULTURE COMMUNICATION” COURSE

Yuan Zhang^a^, Li Zhang^b^

^*a*^ *School of Culture and Media, Xi’an Eurasia University, Xi’an, 710065 China,* ^*b*^ *School of Humanities Management, Shaanxi University of Chinese Medicine, Xianyang, 712099 China.*

**Background:** The education of contemporary college students should proceed from the strategic height of national ideology in combination with many practical problems. Besides, with the development of science and technology, college students’ psychological health education has received more and more attention from the society in a complicated environment.

**Subjects and Methods:** The research and practice based on college students’ psychological health education needs to speed up the exploration and construction of the effective model of college students’ psychological health education on the basis of curriculum construction, and effectively improve the effectiveness of college students’ psychological health education and teaching. The Ideological, political and psychological health education should run through the whole process of online and offline teaching in the course education and teaching, and the mission of imparting knowledge and educating people should be implemented from orally to the practically in the teaching environment.

**Results:** Ideological, political and psychological health education is not only about the mainstream values, but also about the comprehensive development of students’ morality, intelligence, physical fitness and labor from the perspective of college students and the actual needs of social development. The re-analysis and summary from theory to practice and then from practice to theory combine the explicit goal of knowledge theory dissemination with the implicit goal of cultivating contemporary college students’ rational values. Based on the existing teaching resources of the course Film and Television Cultural Communication, it closely follows the key points of curriculum reform and innovation, and realizes the innovative design mode of curriculum system reconstruction in the new media era. The development status of ideological, political and psychological health education in the key course of Film and TV Culture Communication is analyzed to explore the shortcomings and identify the crux of the problem. The specific path of integrating the course of Film and TV Culture Communication aims into Ideological, political and psychological health education is explored from different dimensions.

**Conclusions:** Course content teaching is organically integrated with ideological and political theories teaching as well as psychological health education, and the paths for realizing the common growth of knowledge and skill level and ideological value system are summarized and analyzed. Finally, a reference model is set up for radio and TV editing major in colleges based on the research on the development path of integrating the ideological, political and psychological health education into the course of Film and TV Culture Communication as a case study.

**Acknowledgements**

This work was supported by the Research Project of Humanities and Social Sciences of Ministry of Education (20XJCZH015); Key Scientific Research Project of Shaanxi Provincial Department of Education (20JZ037); Annual Project of Xi’an Social Science Fund (21WT13).

## LHMM082 RESEARCH ON COUNTERMEASURES FOR THE INTEGRATED DEVELOPMENT OF BIOMEDICAL INDUSTRY CHAIN AND INNOVATION CHAIN IN THE YANGTZE RIVER DELTA REGION

Feng Li^a^

^*a*^ *Department of Science Technology and Industry, Yancheng Teachers University, Yancheng, 224007, China.*

**Background:** In the context of the implementation of the Health China strategy, the scale of China’s biomedical industry has been growing. The biomedical industry is an innovation-driven industry, and how to effectively carry out innovation and transfer and transform the innovation results is the key to promote the development of the industry. With the accelerated development of Yangtze River Delta integrated and coordinated development strategy, Yangtze River Delta region has become an important innovation center and production base for biomedicine in China, and the two-way integration of industrial chain and innovation chain has become the key to enhance the core competitiveness of Yangtze River Delta biomedical industry.

**Subjects and Methods:** The author takes Shanghai, Jiangsu, Zhejiang and Anhui in the Yangtze River Delta region as the research object. She uses a combination of quantitative analysis and qualitative research to carry out the study. Then, she comprehensively analyzes the characteristics and trends of the development of biomedical industry in the four regions as well as the problems that exist, and lays the foundation for finding the path of integrated development.

**Results:** The author puts forward countermeasures and suggestions for the integration and development of industrial chain and innovation chain in the biomedical field of Yangtze River Delta around the industrial chain pulling the innovation chain and the innovation chain empowering the industrial chain. For example, consolidate the power of basic research, improve the mechanism of transformation of scientific and technological achievements, improve the mechanism of mutual integration and interconnection, and create an industrial innovation ecology.

**Conclusions:** The innovative development of biomedical industry in the Yangtze River Delta region should closely follow the two key words of “integration” and “high quality,” continuously optimize the regional industrial distribution and create a world-type industrial cluster. We should actively explore the establishment of the Yangtze River Delta biopharmaceutical collaborative innovation mechanism, seek feasible ways for high-efficiency and high-quality development, establish collaborative innovation alliances covering different industries such as government, enterprises, universities, research institutions, hospitals and financial institutions, and efficiently connect the “first kilometer” and “last kilometer” of scientific and technological innovation and technology transformation. It will cover all the innovation elements required for the development of biomedical industry, thus promoting the further optimization and improvement of the pharmaceutical innovation ecology in Yangtze River Delta and even in China.

**Acknowledgments**

The Jiangsu Provincial University Philosophy and Social Science Research Project (2021SJA1877) financially supported this work.

## LHMM083 THE CONTEMPORARY CONSTRUCTION OF ORIENTAL TRADITION AND CHINESE FILM AESTHETICS: AN ANALYTICAL PERSPECTIVE BASED ON NATIONAL AESTHETIC PSYCHOLOGY

Yanfa Wu^a^, Changyuan Zhu^b^, Pei Xue^c^

^*a*^ *Anhui University of Finance and Economics, Anhui 233030, China,* ^*b*^ *Anhui University of Finance and Economics, Anhui 233030, China,* ^*c*^ *National University of Malaysia, 43600 UKM Bangi Selangor, Malaysia.*

**Background:** Aesthetic psychology is a discipline that studies the psychological activities and laws of people in aesthetic appreciation activities. It is a branch of psychology and an interdisciplinary subject of aesthetics and psychology. Aesthetics is an appreciation activity of “appreciating the beauty of things or works of art.” Psychology refers to the process of human brain reflecting objective reality, such as feeling, perception, thinking, emotion, etc. Psychology is to study the laws of human psychological process. As the name implies, aesthetic psychology can be defined as a discipline that studies the psychological process and laws of people in aesthetic appreciation activities. It not only explains the generation of people’s aesthetic experience and the process and laws of knowledge, emotion and meaning, but also the aesthetic relationship between people and art, nature and society. As an important paradigm of film and television culture, Chinese film has long formed the rich and unique aesthetic interest and cultural resources of the Oriental nation because of the influence of the aesthetic psychology and geocultural tradition of the Oriental nation, especially its own long history and humanistic spirit, deep aesthetic psychology and artistic tradition.

**Subjects and Methods:** The process of aesthetic psychology is the track of various aesthetic psychological activities in aesthetic appreciation activities. Human’s psychological process is the combination of cognitive process, emotional process and will process, namely, knowledge, emotion and will. Human’s aesthetic psychological process also includes aesthetic cognition, aesthetic emotion and aesthetic will. But the particularity of aesthetic psychological process is that “in this process, the emotional process is particularly active and strong, and the cognitive process and will process are contained in it.” The aesthetic psychological activity is a series of dynamic changes, and the aesthetic psychological process is the description of the unfolding law of the aesthetic psychological activity. This paper aims to discuss the cultural context of the rise of Chinese films and the uniqueness of film aesthetics from the perspective of the mutual influence of Asian geocultures, the aesthetic psychology of Oriental nations and the integration of poetic aesthetics in Oriental culture, then deeply analyzes and explains the relationship between the deep influence of Chinese traditional culture, national aesthetic psychology, literary and art concepts and the construction of Chinese contemporary films.

**Results:** Generally, the process of individual aesthetic psychology begins with the establishment of aesthetic attitude. Then, the aesthetic object has a series of aesthetic psychological factors: form, representation, performance, symbol, meaning, which act on human senses and trigger human aesthetic psychological activities. Through the comprehensive effect of aesthetic perception, aesthetic imagination, aesthetic understanding, and aesthetic emotion, they jointly promote the generation and accumulation of aesthetic experience, and ultimately develop human aesthetic psychological structure. This paper mainly concentrates and discusses on three theme about “geoculture and Oriental poetic aesthetics” and “roots of tradition and the genes of Chinese film aesthetics” and “breaking through the encirclement of tradition and the current Chinese film aesthetics,” Combined with contemporary Chinese filmmakers how to inherit the advantages of traditional aesthetics, realism and poetic aesthetic culture, at the same time, how to break through the national aesthetic psychology, national cultural tradition, and appropriately absorb and learn from the excellent achievements of western film ideological trend and aesthetic theory, so as to build the system and ideas of Chinese contemporary film aesthetics. And demonstrate the film works with Chinese national aesthetic style to the world, communicate and dialogue with other western film and television aesthetic culture.

**Conclusion:** Aesthetic experience and aesthetic psychological structure promote each other, that is, the generation and accumulation of aesthetic experience can broaden the individual’s aesthetic psychological structure, and those who enrich the aesthetic psychological structure have high efficiency in all aesthetic psychological activities. Facing new aesthetic objects and acquiring new types of aesthetic experience, they can quickly accept, assimilate, adapt and balance. To sum up, the enrichment of aesthetic psychological structure means that the aesthetic appreciation ability is strong. Aesthetic appreciation activities directly promote the generation and accumulation of aesthetic experience, and then bring about the development of aesthetic psychological structure. Under the influence of aesthetic psychological factors in the aesthetic object, individual aesthetic psychological activities continue to occur, promote the generation and accumulation of aesthetic experience, and ultimately promote the continuous adjustment and construction of aesthetic psychological structure. Chinese national aesthetic psychology and national traditional aesthetic elements have always played a decisive and intrinsic role as the heavy cultural gene in Chinese film creation. With the increasingly active and frequent cross-cultural exchanges between China and foreign countries, the aesthetic taste and artistic experience of foreign nations have been reasonably absorbed through the creation of

the national aesthetic psychology, which has promoted the renewal of the aesthetic psychological mechanism of Chinese nations, and the national aesthetic psychology has developed continuously and healthily on the track of nationalization and modernization, and Oriental aesthetic psychology, Oriental aesthetics and traditional culture are playing a more and more subtle effect in Chinese film production, it also makes the context of Oriental aesthetics and modern national aesthetic psychological elements injected more into domestic film production.

## LHMM084 RESEARCH ON THE DEVELOPMENT STRATEGY OF INFORMATION LITERACY EDUCATION FOR UNIVERSITY GRADUATE STUDENTS IN THE NEW ERA BASED ON PSYCHOLOGICAL EMOTIONAL VALUE

Yang Shen^a,b^, Niran Sutheeniran^a^, Jittawisut Wimuttipanya^a^, Kulsirin Aphiratvoradej^a^, Xiang Wang^a^

^*a*^ *Bansomdejchaopraya Rajabhat University, Bangkok, 10600, Thailand,* ^*b*^ *Zunyi Medical University, Zunyi, Guizhou 563003, China*

**Background:** Emotional psychology is a branch of psychology that studies human emotions. It is an important perspective of talent training and modern education development in the new century. Postgraduate education is the main way to cultivate high-level talents, and it shoulders the dual mission of cultivating top-notch innovative talents and realizing a strong country in science and technology. At present, information literacy has been listed as one of the seven core qualities in the world, and for postgraduates, it is also an important basis to meet their scientific research activities and innovative development. From the perspective of personal development, information literacy is also an important guarantee for the all-round physical and mental development of young people. Up to now, few scholars have paid attention to information literacy education for postgraduates, and the theoretical research and practical development are not ideal. Therefore, the formulation of development strategies has become an important issue that educators need to solve jointly.

**Subjects and Methods:** According to the theoretical hypothesis, we use Amos to construct the emotional psychological model of college graduate students, and use the maximum likelihood estimation (MLE) to estimate the parameters of the model. The dimension of experience value perception includes three sub-dimensions: college graduate students’ emotional value perception, postgraduates’ emotional value perception and college graduate students’ emotional environment perception, learning needs and research abilities are also very different. According to the science of education management, pay attention to the role of psychological and emotional value in personnel training and education development, through the methods of literature research, case analysis and comparative research, this paper re-examines the connotation of postgraduate information literacy education, and fully draws on the practical experience of postgraduate information literacy education at home and abroad, to lay a foundation for formulating the development strategy of information literacy education for postgraduates.

**Results:** This volume adopts postgraduates emotion scale. The original scale consists of 32 items. Helmreich and Stapp (1974) modified the scale and divided it into two independent 16-item scales to shorten the test time. The composition of the two scales follows the following criteria: the correlation between the subscale and the total scale is equivalent, the average score between the scales and between different genders is equal, the distribution of the scores is equal, and the corresponding factor structure. The correlation coefficient between the two subscales and the 32-item version of the total scale is 0.97, and the correlation coefficient between them is 0.87. Many researchers who use TSBI only use one subscale. The 32 versions of TSBI factor analysis have produced a large factor item and four theoretically related factor items: confidence, dominance, social ability, social withdrawal or relationship with authority. The subjects answered these statements with a 5-level score, with a total score range of 0-64. The study found that information literacy education for postgraduates is fundamentally different from undergraduate, focusing on helping students form a comprehensive ability to retrieve information, evaluate information, distinguish information, and innovate information. Meanwhile, through statistical analysis of questionnaire survey, it can be found that there are four factors influencing the development of information literacy education for postgraduates in Guizhou Province, the average level is extremely high (‾X=4.76).

**Conclusions:** From the perspective of planning and education management, resource input guarantee, education content construction, evaluation and optimization, the researchers formulated four effective development strategies, including strengthening top-level design, leveraging the advantages of digital technology, exerting the synergistic effect of multiple subjects, and focusing on the postgraduate education standard, which provides the development strategy of information literacy education for postgraduates in the new era based on psychological emotional value, to improve the management level of postgraduate education and the innovation and development of information literacy education.

**Acknowledgements**

1. Humanities and Medicine Research Center project in university in Guizhou Province “Research on the Optimization Path of Discipline Services in Medical Universities from the Perspective of Digital Humanities”. 2. The key project of postgraduate education and teaching reform in Guizhou province “Research on the development system of information literacy education for postgraduates in the new era” (No. YJSCXJH2018020).

## LHMM085 INTERNET USE, SOCIAL INTERACTION AND SOCIAL STATUS: FROM THE PERSPECTIVE OF EMOTIONAL REGULATION AND MENTAL HEALTH

Jianyu Chi^a^, Haotian Liu^a^, Yanfeng Lei^a^

^*a*^ *School of Economics and Management, Communication University of China, Beijing 100024, China.*

**Background:** With the rapid growth of China’s economy, people’s material living standards have increased and they have begun to focus not only on the absolute level of income, but also on their relative status in society. The Internet has been integrated into every aspect of people’s lives in recent years and may influence people’s perceptions of their social status. Based on this, we investigate the effect of Internet use on subjective social status and status identification bias, and explore the potential paths of influence. Compared with the public opinion in the era of traditional media, microblog public opinion shows different characteristics, among which the strong expression of public emotion and behavior is important and has practical impact.

**Subjects and Methods:** The data source for this paper is China General Social Survey 2017 (CGSS2017). We measure subjective social status (SSS) through the scale therein. We use factor analysis to combine income, party membership (CPC), and education to derive objective social status (OSS). And status identification bias (SIB) is obtained by subtracting the objective social status from the subjective social status. We analyze the effect of Internet use frequency on them with ordered probit regressions and verify the mediating role of online and offline sociation frequency, respectively. We conduct robustness checks by replacing the independent and mediating variables, and use the average frequency of Internet use in the individual’s province as an instrumental variable to issue endogeneity with conditional mixed process (CMP) and bivariate ordered probit regression (bioprobit). In addition, we analyze the heterogeneity across regions, gender, and age groups. This paper takes psychological research as the main orientation, and pays attention to the public emotional factors in microblog public opinion. The main research methods are as follows: 1 literature method, through the collection and analysis of public opinion research, microblog research and public sentiment research, this paper selects the content related to this research and analyzes, arranges, synthesizes and uses it. Based on the above research, this paper puts forward a new dimension of public opinion research, and tries to innovate in research content, research perspective and research methods. 2. Case analysis method: On the basis of combing the context of the event, conduct text analysis and data statistics, register microblog through real name and interact with other users, and observe and collect relevant materials in the process of interaction. Questionnaire survey method in order to catch a glimpse of the leopard. 3. Questionnaire survey, using SCL-90 questionnaire, 208 microblog creators were selected to investigate and analyze the emotional and behavioral factors. Ten factors include somatization, anxiety, compulsion and so on.

**Results:** Because DACL evaluated the transient mood, the retest consistency was not high (Lubin, 1981). After one week, the retest consistency was 0.19, 0.24 and 0.22 in the second component table. In aggregate validity, DACL was moderately correlated with MMPI-D (r=0.25-0.53), and so was BDI (r=0.38-0.66). This table is moderately correlated with the clinical overall depression evaluation (male r=0.52, female r=0.23, total r=0.35) and SDS (r=0.41). However, the correlation between SDS and the second component table is slightly higher (r=0.51-0.64). Internet use increases subjective social status, with frequency of offline sociation playing a mediating role in this process. Internet use leads to status identification downward bias, i.e., subjective social status tends to be lower than objective social status. And frequency of online sociation plays a mediating role in this process. In response to the seemingly contradictory findings that Internet use raises subjective social status but leads to status identification downward bias, we further investigate the effect of Internet use on objective social status and find that Internet use also raises objective social status. And the positive effect of Internet use on objective social status is greater, which is the intuitive cause of the status identification downward bias. Heterogeneity analysis shows that Internet use has a relatively weak effect on the subjective social status of the central region group, the female group, and the middle-aged and elderly group.

**Conclusions:** Internet use does increase individuals’ subjective social status, and it not only enhances offline connections among individuals, but also increases objective social status, which is the basis of subjective social status, and thus increases subjective social status. Internet use leads to status identification downward bias. Based on the reference group theory, we suggest that there may be three reasons: “survivorship bias” makes information in the Internet more about individuals in the middle and upper social status; the interaction of the pursuit of higher status by individuals and the “information cocoon”; other individuals’ exaggeration of objective social status constituents such as income and education in the Internet due to vanity. These raise the objective social status of the individual’s reference group and cause the status identification downward bias.

**Acknowledgements**

This work was supported by a project grant from the Asia Media Research Centre, Communication University of China (Grant No. AMRC2021-1).

## LHMM086 A STUDY OF CHINESE COLLEGE ENGLISH TEACHERS’ WELL-BEING FROM THE PERSPECTIVE OF MENTAL HEALTH

Tingting Wang^a^

^*a*^ *Department of Foreign Languages, Bengbu University, Bengbu, Anhui 233030, China*

**Background:** Happiness is the highest wish of all humankind. More and more people have begun to pay attention to well-being, and the pursuit of happiness has become the trend of the times. If the happiness index, like the gross domestic product (GDP), becomes the measurement standard of a country’s development level, then in the education system, the happiness index of teachers and students will also become the measurement standard of a college’s development level. However, research shows that in recent years, teachers’ well-being has declined precipitously. There is not much research on the well-being of college teachers in China, and it is not systematic. College English in China is undergoing an all-around teaching reform. As a particular group of college English teachers who undertaking college English teaching for English majors and non-English majors, it is an essential medium for teaching reform. From an ecological perspective, college teachers’ professional well-being is explored, and teachers’ professional development is promoted; it is the internal requirement of curriculum reform, related to the process of reform, and related to the development of colleges.

**Subjects and Methods:** Among all NRPS outcome variables, income is the most critical one, because all other outcome variables are determined by income. Therefore, this study uses national representative data in 2013 to focus on the impact of NRP on the income of English teachers. Then, in the regression discontinuity design (RDD), we use quantile regression to show the income impact of NRP on different income groups, especially low-income teachers, in order to answer this question from the perspective of the heterogeneous income impact of NRP. At the same time, we analyzed the correlation between emotional regulation ability and healthy behavior. The research selected 200 college English teachers from several comprehensive undergraduate universities in China as the research objects, and conducted a questionnaire survey by combining qualitative and quantitative research methods to maximize the reliability and validity of the research conclusions. The research explored the well-being of college English teachers from an ecological perspective.

**Results:** In the process of the influence of life events on anxiety, emotional response plays a mediating role, and psychological resilience plays a regulating role. Life events are regulated by psychological elasticity through the mediation of emotional response to anxiety. That is to say, the higher the psychological elasticity, the lower the anxiety by adjusting the impact of life events through coping styles; On the contrary, the lower the level of resilience, the more the impact of life events on anxiety can be adjusted and increased through coping styles. The well-being of college English teachers comes from the satisfaction of needs, and the satisfaction of needs will bring three different kinds of happiness: material happiness, interpersonal happiness and spiritual happiness. The sum of these three kinds of happiness constitutes the overall well-being of college English teachers. The material happiness and spiritual happiness of college English teachers are an inseparable combination of happiness. College English teachers’ well-being is also a dialectical unity of individuality and sociality. As shown in the survey, the overall well-being of college English teachers in China is not high.

**Conclusions:** The results showed that society, colleges and teachers should work together, through internal incentives and external drivers, to help teachers experience more happiness in their career development.

## LHMM087 A MATHEMATICAL MODEL FOR THE DESIGN OF LIFE NECESSITIES SUPPLY SCHEME BASED ON SEMANTIC MENTAL HEALTH

Qingyan Dong^a^, Xuejun Zhou^a^, Lei Ye^a^, Maodong Tian^a^, Jiating Qin^a^

^*a*^ *College of Mathematics and Statistics, Huanggang Normal University, Hubei, Huanggang 438000, China.*

**Background:** Mental health refers to that all aspects of psychology and activities are in a good or normal state. The ideal state of mental health is to maintain a state of good character, normal intelligence, correct cognition, proper emotion, reasonable will, positive attitude, proper behavior and good adaptation. Mental health is affected by both heredity and environment, especially the upbringing style of the original family in childhood, which has a great impact on the development of mental health. Mental health highlights the ability to maintain good communication or cooperation with other people in social, production and life, and to deal with various situations in life. A number of large-scale COVID-19 outbreaks have emerged across China, seriously affecting the public health. In response, the Chinese government has implemented a strict epidemic prevention and control policy, that is, in the event of a local epidemic, a partial closure of the affected areas to stop the chain of transmission. Subsequently, the scientific management of people’s living essentials during the COVID-19 lockdown becomes an issue worth studying.

**Subjects and Methods:** The prototype of FNE scale (Watson and Friend, 1969) contains 30 “yes and no” items, of which the positive and negative scores are roughly the same. The revised Concise Scale (Leary, 1983) contains 12 items in the original scale and is graded according to 5 levels (1=completely inconsistent with me; 5=extremely consistent with me). The score range of the original FNE scale is from. (minimum FNE) to 30 (maximum FNE). The concise scale ranges from 12 to 60. The opposite of high FNE is that it does not take advantage of others’ evaluation, and it is not necessarily expected or needs to be positive. The average score of 205 groups in the original table is 15.5 (SD=8.6), and the score is rectangular distribution. This paper takes Changchun City in March 2022 as a case. We employ mathematical modelling to design the living essentials supply scheme during the COVID-19 lockdown based on the data. Specifically, we use regression analysis and K-means clustering analysis methods to model and determine the number and location of the delivery points of living essentials. Using the demand for vegetable packs, the total number of docking and self-picking, the distribution time series and the number of residents as input indicators and the distribution of vegetable packs as output indicators, we adopt the Data Envelopment Analysis (DEA) method to evaluate the vegetable packet supply scheme.

**Results:** It can be found that the number of roads and segregated populations are closely correlated with the number of drop-off points. The healthy personality, mental health and psychological performance of residents are above the middle level. The healthy personality of residents has significant difference in whether they are first-team players or not, and their mental health has significant difference in different sexes; There are significant differences in psychological performance at different ages. There is no significant difference in healthy personality between different sexes, ages, and whether or not they are only children. There was no significant difference in mental health in whether the only child was born or not. There is no significant difference in psychological performance between different genders, whether only child or not, and age. There is a significant positive correlation between healthy personality, mental health and psychological performance of residents. The healthy personality of residents has a significant positive predictive effect on mental health. Sound personality has a significant positive predictive effect on psychological performance. Mental health has a significant positive predictive effect on mental performance. Sound personality can positively predict the level of mental health and psychological performance of residents. Healthy psychology can improve residents’ psychological skills and abilities, thus promoting the play of psychological performance. Residents’ mental health plays a partial intermediary role between sound personality and psychological performance. We also find four drop-off points, i.e., Green Park, Erdao District, Economic Development Zone and Automobile Development Zone, and get six optimum locations for stockpiles and nine potential alternative sites. Moreover, the optimized supply scheme can be determined.

**Conclusions:** To verify the results of the model, we compare them with the actual data. Error analysis shows that the maximum relative error between the two is no more than 12%. It can indicate that the proposed models are objectively and reliability. Our study solves the problem of scientific management of living essentials during the COVID-19 lockdown. While comprehensively improving the efficiency of epidemic prevention and control, we strive to save the personnel input and expenditure of relevant departments, and lay a solid theoretical and practical foundation for future emergency management programs.

**Acknowledgements**

This work was supported by a project grant from Science and Technology Foundation of Hubei Provincial Department of Education of China (Grant No. B2021232), and Graduate Workstation of Huanggang Normal University (Grant No. 5032022014).

## LHMM088 CONSTRUCTION OF FINANCIAL RESTATEMENT RISK PREDICTION MODEL OF LISTED COMPANIES BASED ON INVESTOR SENTIMENT THEORY AND XGBOOST ALGORITHM

Pengxiang Hu^a^, Shuqi Li^a,b^

^*a*^ *Dongfang College, Zhejiang University of Finance & Economics, Haining 314400, China,* ^*b*^ *School of Finance and Economics, Jiangsu University, Zhenjiang 212013, China.*

**Background:** Emotional stability refers to the situation that people’s emotional state fluctuates with the change of external (or internal) conditions. Some people with relatively stable emotions are not easy to cause strong emotional reactions or slow emotional reactions for general situations. For example, it is easier to control your emotions when you encounter major life events such as career success or failure. People with unstable emotions are prone to emotional reactions to the occurrence of events, and trivial things in life can also lead to strong emotional changes. Emotional stability is one of the indicators that psychology judges a person’s personality traits. The most widely used Eysenck emotional stability test can be used to determine whether there is inferiority complex, depression, anxiety, obsessive-compulsive disorder, dependence, hypochondriasis and guilt. Eisenck pointed out that people with emotional (neurotic) instability are moody and easily excited; People with emotional (neurotic) stability react slowly and slightly, and can easily recover calm. He further pointed out that emotion (neuroticism) is related to the function of vegetative nervous system, especially sympathetic nervous system. As an important evaluation of enterprise development status, enterprise financial report directly affects the scientificity of stakeholders’ investment decisions based on its information quality, and financial restatement caused by misstatement will seriously hinder the future development of enterprises. In the meantime, with the rise of behavioral finance, the impact of irrational behaviors such as investor sentiment formed by psychological and emotional factors of investors on the financial market and the enterprise level has gradually attracted the attention of researchers. To a certain extent, the earnings management motivation of the management is further strengthened to cater to the investors’ sentiment, which may increase the probability of financial restatement.

**Subjects and methods:** Sixteen personality factors scale is one of the personality measurement tools, compiled by R.B. Carter, professor of Illinois State University in 1949. It includes 16 personality traits: sociality or enthusiasm, intelligence or intelligence, stability or emotional stability, bullying or dominance, excitement or liveliness, persistence or rule consciousness, daring or social boldness, sensitivity, suspicion or vigilance, fantasy, sophistication or privacy, anxiety, experimental or change openness, independence or autonomy, self-discipline or perfection, and tension. The correlation coefficient of these personality traits is very small. Each question is in the form of a declarative sentence. There are 187 questions in total. The answers are divided into “yes,” “no” and “between the two.” The subjects with reading ability above senior high school should complete it within 45 to 60 minutes. The scale is widely used in psychological counseling, academic and employment guidance. This paper uses the scale to conduct a questionnaire survey on 45 testers. This article explores how to use machine learning to predict the financial restatement of listed companies. The paper takes the restated and non-restated Chinese listed companies from 2017 to 2021 as samples to obtain 4535 groups of restated data and 4535 groups of non- restated data, and adds the identification of investors’ psychological factors to the traditional analysis when selecting features, a financial restatement prediction model of listed companies based on XGboost algorithm was proposed to predict the financial restatement probability of listed companies. By filtering out the low importance variables and supplementing the missing data, the data is input into six models, including the XGboost model. Then it analyzes and compares the quality of financial restatement behavior of listed companies.

**Results:** First, we tested the effect of emotional attention on emotional consistency. In the first experiment, it was found that attention to emotion eliminated the influence of consistency effect on the subject’s impression. Experiment 2 further explored the effect of emotional stability on emotional consistency under the condition of emotional attention. The experimental results showed that only the highly stable individuals eliminated the effect of consistency effect after emotional attention, while the low stable individuals’ impression of faces was still consistent with their emotional state. This shows that the adjustment of consistency effect after paying attention to emotion is still restricted by personality factors. In addition, “positive bias” was found in high emotional stability. Combined with the analysis of investor sentiment features, this paper compares the prediction ability of XGBoost, KNN, Adaboost, MLP, Light GBM and SVM algorithms on financial restatement. It is found that XGBoost performs better than other algorithms in accuracy, recall, precision, F1 score and AUC and has considerable early warning effect.

**Conclusions:** By introducing XGBoost integration method into financial restatement prediction research, stakeholders can use this model to help judge whether listed companies have financial restatement behavior. This model will help promote the cross research of machine learning and accounting, and improve the quality of financial information disclosure of listed companies. In addition, the investor sentiment features increase the prediction effect of the model, which to some extent prove the significance of behavioral finance on enterprise research.

## LHMM089 RESEARCH ON THE POLICY IMPROVEMENT OF IMPROVING THE GOVERNMENT’S SELF-DEVELOPMENT ABILITY IN MINORITY AREAS FROM THE PERSPECTIVE OF ETHNOPSYCHOLOGY: AN EMPIRICAL ANALYSIS BASED ON EIGHT ETHNIC PROVINCES AND AUTONOMOUS REGIONS

Zhaoping Tian^a^, Dan Hu^a,b^, Tao Xiao^a,c^, Yongjun Han^a^

^*a*^ *Institute of Industrial Development and Governance Innovation, Zhejiang Guangsha Vocational and Technical University of Construction, Jinhua 322103, Zhejiang, China,* ^*b*^ *Graduate School of Business, Universiti Tun Abdul Razak, Kuala Lumpur 50400, Kuala Lumpur, Malaysia,* ^*c*^ *School of Education and Humanities, Universiti Tun Abdul Razak, Kuala Lumpur 50400, Kuala Lumpur, Malaysia*

**Background:** Ethnic psychology should be a comprehensive and cross disciplinary subject integrating ethnology and psychology. The psychology of the poverty-stricken groups in ethnic minority areas is the root of endogenous motivation and intellectual support, while the psychological problems of the poverty-stricken groups are the root cause of economic poverty and the direct constraint factor affecting the effectiveness of poverty alleviation. If the Relative Poverty in ethnic areas is not redressed, the relative development gap will continue to widen, and perpetuate psychological imbalance, not to mention imbalance in ethnic and regional relations. Emphasizing the construction of the Chinese national community is to promote the full development of ethnic minority areas, enhance their self-development ability, sustainable development and healthy development ability, and promote the coordination and balance of development between ethnic minority areas and developed areas. A long-term solution to resolve the relative poverty in China’s ethnic areas can be found through improving the self-development ability of the governments in ethnic regions.

**Subjects and Methods:** National psychology is a science that studies national psychological phenomena. National psychology is a combination of psychological processes such as cognition, emotion and will of a particular nation and individual psychological characteristics such as ability, temperament and personality. The elimination of poverty, the realization of the strategic goal of a moderately prosperous society in all respects on schedule, and the steady and lasting income growth of the people of all ethnic groups can greatly boost their confidence in development, lay a solid economic foundation for the follow-up development of the people of all ethnic groups, provide the necessary conditions for full development, and stimulate their endogenous momentum to move forward. The empirical analysis of the eight ethnic provinces showed that the self-development capabilities of the governments in ethnic regions were relatively weak; though the gap between economic regulation and public service had narrowed, the gap between social security and environmental protection had widened.

**Results:** The subjects were tested with BAI and SAS scales and the results were analyzed. The correlation coefficient is 0.828. The result shows that BAI is significantly positively correlated with the total score of SAS objective evaluation. To prove the effectiveness of BAI in the application of mental health. The sensitivity and specificity of BAI were positive with BAI ≥ 40 and ≥ 45, respectively. The false positive rate, false negative rate, sensitivity and specificity were obtained to evaluate the evaluation effect of BAI on anxiety. The results showed that the sensitivity (91.66%) and specificity (91.25%) of BSI were relatively balanced when the BSI was ≥ 45. The self-development capacity of the government in ethnic areas is in dire need of improvement. China’s national strategy should place emphasis on helping minority areas improve their self-development capability.

**Conclusions:** We observed that the state’s differentiated policies to improve the government’s self-development ability in ethnic areas are targeted at fiscal transfer payment and mutual interest cooperation. Intensified measures on these two policies will build a solid foundation in constructing a united Chinese national identity while promoting sustainable development in the ethnic regions.

**Acknowledgements**

This work was supported by a project grant from NSSFC “Research on Long-term Mechanism for Solving Relative Poverty in Ethnic Minority Areas” (Grant No.20BMZ14).

## LHMM090 INCENTIVIZING THE ENTREPRENEURIAL MOTIVATION AND DEDICATION TO INNOVATION BY GOVERNMENT POLICIES: A MODERATING ROLE OF POLICY IMPLEMENTATION AND PSYCHOLOGICAL CONTRACT FULFILLMENT

Zhuo Peng^a^, Xianwei Zhang^a^

^*a*^ *School of Economics, Shenzhen Polytechnic, Shenzhen, 51800, China.*

**Background:** The psychological contract is a kind of cooperation between the individual’s contribution and the organization’s desire, and the organization will provide for the individual’s expectation. Although it is not a tangible contract, it does play the role of a tangible contract. His meaning can be described as a state in which the conditions for the growth of enterprises and the development of employees are not specified in a contract, and it is impossible to specify them because they are dynamic, but enterprises and employees can still find their own “focus” of decision-making, which is regulated by a contract. Entrepreneurs are motivated by governments worldwide to embrace and enforce technological innovation for businesses to have a competitive edge in the global arena. This paper aims to explore whether High-Tech enterprises are motivated by government policies incentivizing scientific and technological innovation and what is the psychological impact on entrepreneurs from the benefits of such policies.

**Subjects and Methods:** 323 entrepreneurs of High-Tech companies in Shenzhen, China, were surveyed between January and June 2022 on their appraisal of government policies supporting technological innovation administered by the local government. SPSS 27 and PLS-SEM 4.0 are applied for the analysis of the data generated in the survey.

**Results:** The empirical findings indicate: a) Government policies have a significant positive impact on entrepreneurial drive in technological innovation; b) An idealized policy design, efficiency in policy implementation and policy environmental factors exert a moderating effect on incentivizing the entrepreneurial efforts in technological innovation via public polices; c) Transactional psychological contract fulfillment perceived by the enterprises is a key moderator in the relationship in-between government as the public policy maker and the enterprises, the public policy beneficiaries. The main body of psychological contract is the psychological state of employees in the enterprise, and the three basic concepts used to measure the psychological state of employees in the enterprise are job satisfaction, job participation and organizational commitment. In organizations such as enterprises that focus on economic activities, employee job satisfaction is the focus and key of enterprise psychological contract management. The purpose of psychological contract management is to achieve employees’ job satisfaction through human resource management, and then realize employees’ strong sense of belonging to the organization and high investment in work. Therefore, in order to achieve the most effective allocation of human resources, enterprises must fully participate in the EAR cycle of psychological contract, and achieve the expectations of employees by influencing the EAR cycle. The so-called EAR cycle refers to the process of psychological contract establishment (Phase E), adjustment (Phase A) and realization (Phase R).

**Conclusion:** The psychological contract is based on the prediction of the future of the enterprise. When the reality deviates from the prediction, the adjustment is inevitable. Enterprises should communicate with employees in a timely manner to find out what is new, so they are expected to make adjustments. Especially when the situation of the enterprise has changed significantly, which causes the psychological fluctuation of employees, timely communication at the top can reduce the psychological burden of employees and reduce the negative impact. Grounded in the empirical research, this paper proposes that entrepreneurial motivation and dedication to technological innovation can be incentivized by government policies, which can be achieved by clearing the informational barriers among implementing organizations in the administration, improving the efficiency in the government policy implementation process, creating an open and transparent public policy environment with a legal oversight and facilitating a strong psychological contract fulfillment.

**Acknowledgements:** Xianwei Zhang is the Corresponding author (zxw5460@szpt.edu.cn). This work was supported by a project grant from Guangdong Audit Office (Grant No.22GDSJZC011101: Research on Evaluating the Performance of the Guangdong Special Educational Fund for “Chong Bu Qiang” Higher Education Development Plan), a project grant from Characteristic Innovation Project of Colleges and Universities in Guangdong Province in 2020 (Grant No. 2020WTSCX239): Research on The Operation and Optimization Strategy of Government Cooperation Mechanism From The Perspective of Guangdong, Hong Kong and Macao, and a grant for “Constructing a Legalization Platform in Finance and Taxation in the Guangdong-Hongkong-Macao Greater Bay Area.”

## LHMM091 RESEARCH THE QUALITY OF EVALUATION AND IMPROVEMENT PATHS TO ECONOMIC DEVELOPMENT IN WULING MOUNTAINS FROM THE PERSPECTIVE OF NATIONAL COMMUNITY PSYCHOLOGY AND PSYCHOLOGICAL PERSONALITY

Dan Hu^a,b^, Tao Xiao^a,c^, Zhaoping Tian^a^, Yongjun Han^a^

^*a*^ *Institute of Industrial Development and Governance Innovation, Zhejiang Guangsha Vocational and Technical University of Construction, Jinhua 322103, Zhejiang, China,* ^*b*^ *Graduate School of Business, Universiti Tun Abdul Razak, Kuala Lumpur 50400, Kuala Lumpur, Malaysia,* ^*c*^ *School of Education and Humanities, Universiti Tun Abdul Razak, Kuala Lumpur 50400, Kuala Lumpur, Malaysia.*

**Background:** From the perspective of psychology, the connotation of the consciousness of the Chinese nation community constructed according to the category logic of object, subject and content refers to the psychological state process between unconscious and conscious of the members of various nationalities for the “Chinese nation community.” To strengthen the economic foundation for the Chinese nation’s sense of community, let the people of all ethnic groups share in the fruits of development, and constantly meet their aspirations for a better life will help strengthen the cohesion and centripetal force of the Chinese nation.The issue of “Agriculture Rural areas and Farmers”are fundamental issues concerning the national economy and people’s livelihood, and seriously restrict people’s all-round development. The unbalanced and inadequate development of our country is particularly prominent in the countryside of ethnic minority areas. Personality is the sum of the actual behavior models of individuals. Eisenck has carried out extensive research on personality through various methods such as psychiatric diagnosis, questionnaire, movement test experiment, body measurement, etc., and put forward the theory of personality hierarchy structure, and believed that the theory of type and the theory of trait have some consistency.

**Subjects and methods:** Through the factor analysis of a large number of people’s characteristic data, this paper uses the personality dimension theory to analyze. The first dimension is tropism, that is, introversion and extroversion. The second dimension is emotional stability. The third dimension is psychopathic tendencies. On this basis, this paper revised and adopted the Eysenck Personality Scale. The scale is divided into two types: adult and juvenile, each including four subscales: E is the internal and external subscale; N is neurotic (emotional) subscale; P is the subscale of psychoticism (also known as stubbornness and practicality); L is the lie or escape subscale. The first three represent three independent dimensions of personality structure. L is a validity scale, but it can also measure the naive and naive level. The scale has high validity and reliability and is widely used. The Wuling Mountains is a mountainous range, located in Central China, which situates the Enshi Prefecture and Xiangxi Prefecture and home to many ethnic groups. Researchers who attempt to seek and improve the current situation and characteristics of economic development in Wuling Mountains, first, need to examine the perspectives of economic activity, national income, government income, consumer spending, and quality of life. Therefore, the aim of this research is to explore the economic development of Wuling Mountains by studying family perspectives, business enterprises and government institutions.

**Results:** The healthy personality, mental health and psychological performance of residents are above the middle level. The healthy personality of residents has significant difference in whether they are first-team players or not, and their mental health has significant difference in different sexes; There are significant differences in psychological performance at different ages. There is no significant difference in healthy personality between different sexes, ages, and whether or not they are only children. There was no significant difference in mental health in whether the only child was born or not. There is no significant difference in psychological performance between different genders, whether only child or not, and age. There is a significant positive correlation between healthy personality, mental health and psychological performance of residents. The healthy personality of residents has a significant positive predictive effect on mental health. Sound personality has a significant positive predictive effect on psychological performance. Mental health has a significant positive predictive effect on mental performance. Sound personality can positively predict the level of mental health and psychological performance of residents. Healthy psychology can improve residents’ psychological skills and abilities, thus promoting the play of psychological performance. Residents’ mental health plays a partial intermediary role between sound personality and psychological performance. An empirical analysis was conducted, which revealed there is a relative discontinuation in the quality of development, which created a barrier to the economic development of Wuling Mountains. For purpose of bridging the gap and promoting high-quality ethical economic regions, by constructing characteristic villages, a winning revitalization strategy was implemented. To end extreme poverty and to revitalize Wuling Mountains, one can embrace the “characteristics of a model village” by building and improving the “five dimensional rural integration” development policy of Wuling Mountains.

**Conclusions:** To achieve the goals of rural revitalization strategy, and industrial affluence, the key performance indicator is industrial support especially for cultivating characteristic industries. Fostering the concept of green growth development, ecological efficiency is regarded as a means to promote the many approaches of ecological civilization, and eventually realize there exists a multiple dimensional, multiple space and multiple levels of ecological livability and peaceful coexistence for new beautiful and characteristic villages. There should also be an in-depth promotion should there be changes in traditional customs and dissemination of rural customs and civilization in order to effectively construct characteristic villages into spiritual homes, humanistic homes and harmonious home for the peasants. It is recommended to use a combination of autonomy, morality, and law to govern the rural areas to achieve effective rural governance. Having prosperity in life is not only a sign of improvement of income, or the improvement of clothing, food, housing and transportation, but also the psychological, physiological, and spiritual well-being for the elderly.

**Acknowledgements**

This work was supported by a project grant from NSSFC “Research on Long-term Mechanism for Solving Relative Poverty in Ethnic Minority Areas” (Grant No.20BMZ14).

## LHMM092 PERFORMANCE EVALUATION OF AGRICULTURAL MACHINERY COOPERATIVES IN TRANSFERRING AGRICULTURAL LAND TO ASSIST FARMERS TO INCREASE INCOME BASED ON EMOTIONAL STABILITY

Yuxin Liu^a,b^, Eryang Liu^a^, Jinwu Wang^a^

^*a*^ *Northeast Agricultural University, China,* ^*b*^ *Party School Heilongjiang Provincial Committee of C.P.C (Heilongjiang Academy of Governance), China.*

**Background:** Some people with relatively stable emotions are not easy to cause strong emotional reactions or slow emotional reactions for general situations. For example, it is easier to control your emotions when you encounter major life events such as career success or failure. People with unstable emotions are prone to emotional reactions to the occurrence of events, and trivial things in life can also lead to strong emotional changes. In recent years, with the strong support of national policies, agricultural machinery cooperatives have developed rapidly in Heilongjiang Province and gradually replaced other forms of agricultural machinery operation service organizations, becoming an important organization carrier to realize agricultural scale operation. Heilongjiang Agricultural Machinery Cooperatives, as one of the new agricultural operation subjects, play an important role in promoting agricultural efficiency, increasing farmers’ income and promoting labor transfer.

**Subjects and Methods:** Emotional stability is one of the indicators that psychology judges a person’s personality traits. The most widely used Eysenck emotional stability test can be used to determine whether there is inferiority complex, depression, anxiety, obsessive-compulsive disorder, dependence, hypochondriasis and guilt. Eisenck pointed out that people with emotional (neurotic) instability are moody and easily excited; People with emotional (neurotic) stability react slowly and slightly, and can easily recover calm. He further pointed out that emotion (neuroticism) is related to the function of vegetative nervous system, especially sympathetic nervous system. This paper uses the did model and selects Heilongjiang Standardize Agricultural Machinery Cooperatives and relevant farmers as the research objects, and evaluates the performance of Agricultural Machinery Cooperat

**Results:** The cognitive style of field independence has obvious advantages over field dependence in terms of graphic system changes, series relations and abstract reasoning. The overall effect of graphic reasoning of high emotional stability is significantly better than that of low emotional stability. There was no significant difference in the effect of graphic reasoning between introverts and extroverts. Cognitive style, emotional stability and the total score of graphic reasoning have significant positive correlation, and the positive predictive power of cognitive style ranks first. The model estimation results show that: compared with the farmers who transfer land to the Agricultural Machinery Cooperatives, the total income, wage income, property income and transfer income of farmers are significantly increased, and the operating income is significantly reduced. In the introduced control variables, age is negatively related to the total income of farmers and wage income, and positively related to operational income. The education level is positively correlated with farmers’ total income, wage income and property income. The total family population is positively related to the total income of farmers, operating income and wage income. The value of household assets is positively correlated with wage income.

**Conclusions:** Therefore, Agricultural Machinery Cooperatives, as a new type of agricultural operation subject, have achieved remarkable results in assisting farmers to increase their income. The increase of farmers’ income will directly improve their living standards and effectively promote their mental health. Based on the analysis of the performance of agricultural machinery cooperatives in transferring agricultural land to help farmers increase their income, targeted suggestions are put forward.It will help to promote the healthy development of agricultural machinery cooperatives in Heilongjiang Province and increase farmers’ income.

**Acknowledgements**

Supported by National Social Science Fund Youth Project “Empirical Analysis and Policy Research on Spillover Effect of Farmer Professional Cooperatives under Rural Revitalization Strategy” (Project No.: 19CJY039) as a staged achievement.

## LHMM093 EXPLORATION AND PRACTICE OF COLLEGE TEACHING DURING THE COVID-19 EPIDEMIC BASED ON ROSENTHAL EFFECT

Cuoling Zhang^a^, Deqing Zhang^a^, Yingying Mei^a^

^*a*^ *School of Computer Engineering, Anhui Salian University, Hefei City 230601, China.*

**Background:** Rosenthal effect, also known as “Pygmalion effect” and “interpersonal expectation effect,” is a kind of social psychological effect, which refers to the phenomenon that teachers’ ardent hope for students can dramatically receive the expected effect. It was discovered by American psychologists Rosenthal and L. Jacobson through experiments in 1968. Generally speaking, this effect is mainly because teachers expect different behaviors for high achievers and low achievers, and treat them in different ways, thus maintaining their original behavior mode. With the rapid development of modern information technology and the deep integration of the Internet and higher education, the “Internet plus Education” model has been widely used; In recent years, many Internets public online teaching platforms and curriculum resources have been rapidly built and launched, which has paved the way for online teaching during the COVID-19 epidemic. Teachers’ love, care and expectation for students will play a good role in teaching effect. After obtaining the potential information of students, teachers have expectations, which will be turned into attention and encouragement to students. The teacher’s encouragement will make students strengthen their confidence and thirst for knowledge, and generate a strong desire for progress. Students with poor foundation, lack of self-confidence, and low interest in learning, if only teachers carry out external teaching and transfer knowledge unilaterally, while students have no internal needs and desires, and do not actively accept until consciously acquire knowledge, it is very difficult to improve students’ performance. Accordingly, teachers also inevitably lose confidence in students, lack enthusiasm for education, and teaching and learning fall into a vicious circle that is difficult to extricate.

**Subjects and Methods:** This paper mainly uses the methods of literature, questionnaire and case analysis to conduct in-depth research on the “Rosenthal effect” and its application in the transformation of underachievers in college teaching. The object of this study is 30 students who fail in English in our school. Whether the application of Rosenthal effect in teaching can improve the learning interest and learning achievement of the specific group of underachievers is the main research issue of this paper. During the one-semester experiment, the selected 30 underachievers were given Rosenthal effect teaching intervention. Teachers should fully respect the individual differences of students and have positive expectations for them on the basis of recognizing the differences. In the process of teaching, teachers should be good at finding their shining points, praise and encourage them in time to enhance their confidence in learning. Before and after the experiment, they were investigated with questionnaires to understand their learning interests, and the results of the two collections were used as the pre-test and post-test data of the experiment. The results of the two tests before and after the experiment were also collected as the data of the experiment. This paper expounds the current situation of college teaching under the COVID-19, analyzes many problems existing in online teaching, such as insufficient network bandwidth, difficulties in carrying out experimental teaching, difficulties in monitoring students’ online listening status, and difficulties in assessment, and shares many excellent online teaching tools and free online teaching resources at home and abroad.

**Results:** SPSS19.0 was used to conduct paired sample T test on the above two groups of experimental data. From the results of data analysis, a semester of teaching experiment has proved that the application of Rosenthal effect in teaching has a significant impact on the learning interest of the underachievers and the improvement of their learning achievements. Combined with their own online teaching experience and that of the teachers in the research group, we summarized the closed-loop teaching methods for online teaching implementation, including pre class preparation, specific implementation of teaching in class, integration of ideological and political education in the curriculum, communication and counseling after class, and a reasonable assessment mechanism for online courses. Finally, it summarizes the research of the subject and the advantages of online teaching and puts forward the next research plan.

**Conclusions:** Today, when the epidemic situation is still serious, the article provides a certain reference basis for online classroom teaching and practical teaching of many college teachers and has certain practical value.

**Acknowledgments**

1. Research and Practice of Software Design Curriculum Reform Based on the Integration of Industry and Education (NO: 2020jyxm0556).

2. Course Ideological and Political Demonstration Course - Computer Network (NO: 2021kcszsfkc147).

3. Exploration and Practice of “Network Engineering” Specialty Transformation and Upgrading under the Background of New Engineering Construction (NO: 20zlgc068).

4. Research on teaching mode of python programming curriculum system based on OBE under the background of new engineering (NO: 2021jyxm0444).

## LHMM094 A STUDY ON THE RELATIONSHIP BETWEEN TOURISM INTERACTION AND PSYCHOLOGICAL EMOTION OF CHINESE TOURISTS IN THEIR PRO ENVIRONMENTAL BEHAVIOR

Yue Xiao^a^, Jinguang Liu^a^

^*a*^ *Guilin Tourism University, 541006 Guilin City, Guangxi Province, China.*

**Background:** Situational selection refers to the individual making evasive or close choices about the people and things he will encounter, so as to make certain control over the possible emotions. Situational selection is not a random behavior, it often reflects the individual’s choice of the appropriate environment, which may be conscious or unintentional. When the individual has been in a certain emotion-induced situation, the adjustment of situational response occurs after emotional activation, It refers to the adjustment of emotion through strategies such as strengthening, reducing, extending or shortening the response. Even if certain modifications are made to the emotion, it is still possible to adjust the emotion. The COVID-19 pandemic, is an uncontrollable crisis posing a threat to human health and hygiene. Ecological degradation and environmental pollution exacerbate the threat of the COVID-19 pandemic to public hygiene and health, prompting a reassessment and rethinking of the relationship between man and nature. Although studies have found a positive correlation between air pollution and the spread of emerging infectious diseases (e.g., COVID-19, SARS-COV, HIV), to date, few researchers have considered human behavioural responses to linkages between environmental pollution and the health emergency. This health emergency allows us to analyze to what extent the mechanisms of pro-environmental behaviours can be affected by social interaction. Interpersonal interaction is especially important to Chinese tourists with high collective culture. Therefore, it is of great significance to study the influence mechanism of tourist-to-tourist interaction on Chinese tourists’ pro-environmental behaviours during the COVID-19 pandemic.

**Subjects and Methods:** This study uses Watson and Friend’s (1969) fear of negation scale to measure. The study defines “fear of negation evaluation” (FNE) as being superior to others’ evaluation, being distressed by others’ negative evaluation, and expecting to be negatively evaluated by others. The items in this scale are completely consistent with the above concepts. The prototype of FNE scale (Watson and Friend, 1969) contains 30 “yes and no” items, of which the positive and negative scores are roughly the same. The revised Concise Scale (Leary, 1983) contains 12 items in the original scale and is graded according to 5 levels (1=completely inconsistent with me; 5=extremely consistent with me). The score range of the original FNE scale is from. (minimum FNE) to 30 (maximum FNE). The concise scale ranges from 12 to 60. The opposite of high FNE is that it does not take advantage of others’ evaluation, and it is not necessarily expected or needs to be positive. The average score of 205 groups in the original table is 15.5 (SD=8.6), and the score is rectangular distribution. The average score of the other sample composed of 128 subjects is 13.6 (SD=7.6) a The average score of the sample (n=150) used to prepare the 12-item concise scale is 35.7 (SD=8.1). The subject of this study is to explore the influence of tourist-to-tourist interaction on the processes and outcomes of Chinese tourists’ pro-environmental behaviour in response to the COVID-19 health crisis. This study adopts mixed-method research to comprehend the phenomenon. The quantitative data analysis technique used was Structural Equation Modelling (SEM) using AMOS in SPSS 23.0, whereas the qualitative technique draws from 35 in-depth interviews conducted with tourists living in China to understand the role of tourist-to-tourist interaction in pro-environmental behaviours in the context of the COVID-19 health emergency.

**Results:** The results of evolutionary game in complex networks do not show absolute stability. Obviously, the simulation results are closer to reality. Similarly, the study also found that there was a significant negative correlation between anxiety and evolutionary stability. State anxiety, trait anxiety and environmental anxiety are all positively correlated. The “anxiety” component of personality characteristics plays a great role in the generation of anxiety. The correlation coefficient between LCAS and trait anxiety was 0.408, while the correlation coefficient between LCAS and state anxiety was 0.395. Both reached a significant level (P < 0.01). The qualitative findings confirm that tourist-to-tourist interaction can affect tourists’ pro-environmental behaviour through affection, environmental knowledge, sense of social responsibility and conformity effect. The quantitative findings reveal that there is no direct relationship between environmental knowledge and pro-environmental behaviour. However, this research established a clear link between social responsibility, conformity behaviour and pro-environmental behaviour. It has found that social responsibility and conformity behaviour of the tourist-to-tourist interaction are expected to increase the pro-environmental behaviour of Chinese tourists.

**Conclusions:** This research highlights the influencing path of social interactions among tourists on pro-environmental behaviour in the context of the COVID-19 health emergency. This study is an important complement to previous studies on the interactive antecedents of tourists’ pro-environmental behaviours that paid little attention to the impact of external public health emergencies. The findings can provide useful recommendations for policymakers to identify opportunities behind the COVID-19 health emergency to promote tourists’ pro-environmental behaviours.

## LHMM095 THE IMPACT OF CONSUMER PANIC PSYCHOLOGY ON THE OPERATION STRATEGY OF EMERGENCY SUPPLIES MANUFACTURERS FROM THE PERSPECTIVE OF MANAGEMENT PSYCHOLOGY

Dongci Liu^a^, Yongjiang Chen^a^

^*a*^ *School of Finance, Hunan University of Technology and Business, Changsha 410205, China.*

**Background:** The occurrence of emergency crisis broke the original psychological balance of individuals. The tension in the heart keeps accumulating, and when it accumulates to a certain extent, it forms a panic mentality, which is accompanied by a series of abnormalities, such as high blood pressure, rapid heartbeat, anxiety, tension, and low cognitive ability. There are four indicators to measure panic psychology: physiology, emotion, cognition and behavior. This study starts from two aspects of cognition and behavior, and selects memory and reasoning ability as indicators to measure cognition, and risk-taking behavior and altruistic behavior as indicators to measure behavior. Since entering the 21st century, major public health emergencies such as SARS, Ebola and COVID-19 have broken out frequently around the world, which has generated a large demand for emergency supplies. The general public needs medical masks, disinfectant alcohol, disposable gloves, etc. The effective supply of these emergency supplies is crucial in the early prevention and control of major public health emergencies. Since consumers tend to stockpile large amounts of protective supplies in response to emergencies due to panic, further leading to shortages of emergency supplies and supply disruptions. Therefore, this paper investigates the impact of consumer panic on the operational decisions of supply chain members in the event of a major public health emergency.

**Subjects and Methods:** Different from the previous psychological research methods of self-report scale, this study uses computer software as an experimental tool to design a virtual scene and insert relevant test questions into the scene. In order to increase the authenticity of the virtual scene, according to the urgency of decision-making at different stages of the event development, the time pressure is exerted on the subjects’ decision-making to simulate the emergency crisis to the maximum extent, stimulate the subjects’ panic psychology, and obtain more real panic psychological data. In the formal experiment, many people conduct concurrent experiments and interact with each other to simulate the group interaction of real events. This study attempts to explore the different psychological changes of individuals in the calm period, the development period, the climax period and the ebb period of sudden crisis events. This paper considers the impact of consumer panic on the operational decisions of a manufacturer and a retailer of emergency supplies in the event of a major public health emergency, using a supply chain consisting of a manufacturer and a retailer of emergency supplies. First, by organizing the relevant literature on management psychology and consumer panic psychology, we provide a theoretical basis for the research focus and innovation of this paper; Secondly, under the perspective of management psychology, the consumer panic psychology is introduced to analyze its influence on the decision making of each member of the supply chain and solve for the optimal results under different models. Finally, we use Excel, Maple and other software to simulate the parameters of the constructed model, carry out the analysis of calculation cases, examine the rationality of the psychological game model, and further analyze the impact of changes in parameters such as consumer panic on the results of the psychological game.

**Results:** Panic mood has indirect effect between group panic and emergency behavior. All dimensions of group panic will increase the probability of emergency unsafe behaviors. The influence of individual traits and group factors on unsafe emergency response behavior has two aspects, one is the impact of panic on unsafe emergency response behavior, the other is the direct impact on unsafe emergency response behavior. The stress environment has a direct impact on unsafe emergency behaviors. The objective characteristics of emergency behavior affect unsafe emergency behavior through panic. Male group panic is mainly affected by group behavior, while female group panic is mainly affected by risk information and stress environment. On this basis, from the perspective of passengers and the government, reasonable suggestions are put forward for the control of group panic and behavior during unsafe emergency actions. Under a managerial psychology perspective, it is found that when a major public emergency health event occurs, an increase in consumer panic leads to an increase in the probability of supply disruption, and the psychological game outcome of a financially constrained emergency supplies manufacturer is independent of the degree of consumer panic if it is not financed, and the optimal wholesale price of

the emergency supplies manufacturer is independent of the degree of consumer panic. If the psychological game of the emergency goods manufacturer results in credit financing or pre-sale financing, the optimal wholesale price of the emergency goods product manufacturer will increase and the optimal selling price of the retailer will decrease when the level of consumer panic rises.

**Conclusions:** In a managerial psychology perspective, when a major public health emergency occurs, increased levels of consumer panic can raise the production costs of emergency goods manufacturers, lower the marginal price of products for retailers, and reduce consumer utility.

Therefore, on the one hand, the government’s adoption of relevant policies to reduce consumer panic is conducive to alleviating the problem of shortage of emergency supplies products. On the other hand, manufacturers stockpile spare production raw materials to reduce the loss of emergency supplies manufacturers and maintain the stability of the supply chain.

**Acknowledgements**

This paper is written thanks to the careful guidance of my supervisor and the support and encouragement of my siblings.

